# Supplementary material for: A full formal representation of Arrow’s impossibility theorem
Source: PLoS One. 2026 Feb 24;21(2):e0343069. doi: 10.1371/journal.pone.0343069 (PMC12931778; doi:10.1371/journal.pone.0343069)
Supplement: S1 Appendix — (PDF) [file pone.0343069.s001.pdf]

S1 Appendix: A proof of the impossibility theorem (two individuals and three alternatives)

1 | $H(p) \land H(q) \land p \neq q \land \forall x(H(x) \to (x = p \lor x = q))$ prem.
2 | $A(a) \land A(b) \land A(c) \land a \neq b \land b \neq c \land c \neq a \land \forall x(A(x) \to (x = a \lor x = b \lor x = c))$ prem.
3 | $\boldsymbol{P}(R_{1-1}) \land \ldots \land \boldsymbol{P}(R_{13-13}) \land R_{1-1} \neq R_{1-2} \land \ldots \land R_{13-12} \neq R_{13-13} \land \forall X(\boldsymbol{P}(X) \to (X = R_{1-1} \lor \ldots \lor X = R_{13-13}))$ prem.
4 | $(R_{1-1}(p,a,b) \land \neg R_{1-1}(p,b,a)) \land (R_{1-1}(p,b,c) \land \neg R_{1-1}(p,c,b)) \land (R_{1-1}(p,a,c) \land \neg R_{1-1}(p,c,a)) \land R_{1-1}(p,a,a) \land R_{1-1}(p,b,b) \land R_{1-1}(p,c,c) \land (R_{1-1}(q,a,b) \land \neg R_{1-1}(q,b,a)) \land (R_{1-1}(q,b,c) \land \neg R_{1-1}(q,c,b)) \land (R_{1-1}(q,a,c) \land \neg R_{1-1}(q,c,a)) \land R_{1-1}(q,a,a) \land R_{1-1}(q,b,b) \land R_{1-1}(q,c,c)$ prem.
5 | $(R_{1-2}(p,a,b) \land \neg R_{1-2}(p,b,a)) \land (R_{1-2}(p,b,c) \land \neg R_{1-2}(p,c,b)) \land (R_{1-2}(p,a,c) \land \neg R_{1-2}(p,c,a)) \land R_{1-2}(p,a,a) \land R_{1-2}(p,b,b) \land R_{1-2}(p,c,c) \land (R_{1-2}(q,a,b) \land \neg R_{1-2}(q,b,a)) \land (\neg R_{1-2}(q,b,c) \land R_{1-2}(q,c,b)) \land (R_{1-2}(q,a,c) \land \neg R_{1-2}(q,c,a)) \land R_{1-2}(q,a,a) \land R_{1-2}(q,b,b) \land R_{1-2}(q,c,c)$ prem.
6 | $(R_{1-3}(p,a,b) \land \neg R_{1-3}(p,b,a)) \land (R_{1-3}(p,b,c) \land \neg R_{1-3}(p,c,b)) \land (R_{1-3}(p,a,c) \land \neg R_{1-3}(p,c,a)) \land R_{1-3}(p,a,a) \land R_{1-3}(p,b,b) \land R_{1-3}(p,c,c) \land (\neg R_{1-3}(q,a,b) \land R_{1-3}(q,b,a)) \land (R_{1-3}(q,b,c) \land \neg R_{1-3}(q,c,b)) \land (R_{1-3}(q,a,c) \land \neg R_{1-3}(q,c,a)) \land R_{1-3}(q,a,a) \land R_{1-3}(q,b,b) \land R_{1-3}(q,c,c)$ prem.
7 | $(R_{1-4}(p,a,b) \land \neg R_{1-4}(p,b,a)) \land (R_{1-4}(p,b,c) \land \neg R_{1-4}(p,c,b)) \land (R_{1-4}(p,a,c) \land \neg R_{1-4}(p,c,a)) \land R_{1-4}(p,a,a) \land R_{1-4}(p,b,b) \land R_{1-4}(p,c,c) \land (\neg R_{1-4}(q,a,b) \land R_{1-4}(q,b,a)) \land (R_{1-4}(q,b,c) \land \neg R_{1-4}(q,c,b)) \land (\neg R_{1-4}(q,a,c) \land R_{1-4}(q,c,a)) \land R_{1-4}(q,a,a) \land R_{1-4}(q,b,b) \land R_{1-4}(q,c,c)$ prem.
8 | $(R_{1-5}(p,a,b) \land \neg R_{1-5}(p,b,a)) \land (R_{1-5}(p,b,c) \land \neg R_{1-5}(p,c,b)) \land (R_{1-5}(p,a,c) \land \neg R_{1-5}(p,c,a)) \land R_{1-5}(p,a,a) \land R_{1-5}(p,b,b) \land R_{1-5}(p,c,c) \land (R_{1-5}(q,a,b) \land \neg R_{1-5}(q,b,a)) \land (\neg R_{1-5}(q,b,c) \land R_{1-5}(q,c,b)) \land (\neg R_{1-5}(q,a,c) \land R_{1-5}(q,c,a)) \land R_{1-5}(q,a,a) \land R_{1-5}(q,b,b) \land R_{1-5}(q,c,c)$ prem.
9 | $(R_{1-6}(p,a,b) \land \neg R_{1-6}(p,b,a)) \land (R_{1-6}(p,b,c) \land \neg R_{1-6}(p,c,b)) \land (R_{1-6}(p,a,c) \land \neg R_{1-6}(p,c,a)) \land R_{1-6}(p,a,a) \land R_{1-6}(p,b,b) \land R_{1-6}(p,c,c) \land (\neg R_{1-6}(q,a,b) \land R_{1-6}(q,b,a)) \land (\neg R_{1-6}(q,b,c) \land R_{1-6}(q,c,b)) \land (\neg R_{1-6}(q,a,c) \land R_{1-6}(q,c,a)) \land R_{1-6}(q,a,a) \land R_{1-6}(q,b,b) \land R_{1-6}(q,c,c)$ prem.
10 | $(R_{1-7}(p,a,b) \land \neg R_{1-7}(p,b,a)) \land (R_{1-7}(p,b,c) \land \neg R_{1-7}(p,c,b)) \land (R_{1-7}(p,a,c) \land \neg R_{1-7}(p,c,a)) \land R_{1-7}(p,a,a) \land R_{1-7}(p,b,b) \land R_{1-7}(p,c,c) \land (R_{1-7}(q,a,b) \land R_{1-7}(q,b,a)) \land (R_{1-7}(q,b,c) \land \neg R_{1-7}(q,c,b)) \land (R_{1-7}(q,a,c) \land \neg R_{1-7}(q,c,a)) \land R_{1-7}(q,a,a) \land R_{1-7}(q,b,b) \land R_{1-7}(q,c,c)$ prem.
11 | $(R_{1-8}(p,a,b) \land \neg R_{1-8}(p,b,a)) \land (R_{1-8}(p,b,c) \land \neg R_{1-8}(p,c,b)) \land (R_{1-8}(p,a,c) \land \neg R_{1-8}(p,c,a)) \land R_{1-8}(p,a,a) \land R_{1-8}(p,b,b) \land R_{1-8}(p,c,c) \land (R_{1-8}(q,a,b) \land R_{1-8}(q,b,a)) \land (\neg R_{1-8}(q,b,c) \land R_{1-8}(q,c,b)) \land (\neg R_{1-8}(q,a,c) \land R_{1-8}(q,c,a)) \land R_{1-8}(q,a,a) \land R_{1-8}(q,b,b) \land R_{1-8}(q,c,c)$ prem.
12 | $(R_{1-9}(p,a,b) \land \neg R_{1-9}(p,b,a)) \land (R_{1-9}(p,b,c) \land \neg R_{1-9}(p,c,b)) \land (R_{1-9}(p,a,c) \land \neg R_{1-9}(p,c,a)) \land R_{1-9}(p,a,a) \land R_{1-9}(p,b,b) \land R_{1-9}(p,c,c) \land (\neg R_{1-9}(q,a,b) \land R_{1-9}(q,b,a)) \land (R_{1-9}(q,b,c) \land R_{1-9}(q,c,b)) \land (\neg R_{1-9}(q,a,c) \land R_{1-9}(q,c,a)) \land R_{1-9}(q,a,a) \land R_{1-9}(q,b,b) \land R_{1-9}(q,c,c)$ prem.
13 | $(R_{1-10}(p,a,b) \land \neg R_{1-10}(p,b,a)) \land (R_{1-10}(p,b,c) \land \neg R_{1-10}(p,c,b)) \land (R_{1-10}(p,a,c) \land \neg R_{1-10}(p,c,a)) \land R_{1-10}(p,a,a) \land R_{1-10}(p,b,b) \land R_{1-10}(p,c,c) \land (R_{1-10}(q,a,b) \land \neg R_{1-10}(q,b,a)) \land (R_{1-10}(q,b,c) \land R_{1-10}(q,c,b)) \land (R_{1-10}(q,a,c) \land \neg R_{1-10}(q,c,a)) \land R_{1-10}(q,a,a) \land R_{1-10}(q,b,b) \land R_{1-10}(q,c,c)$ prem.
14 | $(R_{1-11}(p,a,b) \land \neg R_{1-11}(p,b,a)) \land (R_{1-11}(p,b,c) \land \neg R_{1-11}(p,c,b)) \land (R_{1-11}(p,a,c) \land \neg R_{1-11}(p,c,a)) \land R_{1-11}(p,a,a) \land R_{1-11}(p,b,b) \land R_{1-11}(p,c,c) \land (R_{1-11}(q,a,b) \land \neg R_{1-11}(q,b,a)) \land (\neg R_{1-11}(q,b,c) \land R_{1-11}(q,c,b)) \land (R_{1-11}(q,a,c) \land R_{1-11}(q,c,a)) \land R_{1-11}(q,a,a) \land R_{1-11}(q,b,b) \land R_{1-11}(q,c,c)$ prem.
15 | $(R_{1-12}(p,a,b) \land \neg R_{1-12}(p,b,a)) \land (R_{1-12}(p,b,c) \land \neg R_{1-12}(p,c,b)) \land (R_{1-12}(p,a,c) \land \neg R_{1-12}(p,c,a)) \land R_{1-12}(p,a,a) \land R_{1-12}(p,b,b) \land R_{1-12}(p,c,c) \land (\neg R_{1-12}(q,a,b) \land R_{1-12}(q,b,a)) \land (R_{1-12}(q,b,c) \land \neg R_{1-12}(q,c,b)) \land (R_{1-12}(q,a,c) \land R_{1-12}(q,c,a)) \land R_{1-12}(q,a,a) \land R_{1-12}(q,b,b) \land R_{1-12}(q,c,c)$ prem.
16 | $(R_{1-13}(p,a,b) \land \neg R_{1-13}(p,b,a)) \land (R_{1-13}(p,b,c) \land \neg R_{1-13}(p,c,b)) \land (R_{1-13}(p,a,c) \land \neg R_{1-13}(p,c,a)) \land R_{1-13}(p,a,a) \land R_{1-13}(p,b,b) \land R_{1-13}(p,c,c) \land (R_{1-13}(q,a,b) \land R_{1-13}(q,b,a)) \land (R_{1-13}(q,b,c) \land R_{1-13}(q,c,b)) \land (R_{1-13}(q,a,c) \land R_{1-13}(q,c,a)) \land R_{1-13}(q,a,a) \land R_{1-13}(q,b,b) \land R_{1-13}(q,c,c)$ prem.
17 | $(R_{2-1}(p,a,b) \land \neg R_{2-1}(p,b,a)) \land (\neg R_{2-1}(p,b,c) \land R_{2-1}(p,c,b)) \land (R_{2-1}(p,a,c) \land \neg R_{2-1}(p,c,a)) \land R_{2-1}(p,a,a) \land R_{2-1}(p,b,b) \land R_{2-1}(p,c,c) \land (R_{2-1}(q,a,b) \land \neg R_{2-1}(q,b,a)) \land (R_{2-1}(q,b,c) \land \neg R_{2-1}(q,c,b)) \land (R_{2-1}(q,a,c) \land \neg R_{2-1}(q,c,a)) \land R_{2-1}(q,a,a) \land R_{2-1}(q,b,b) \land R_{2-1}(q,c,c)$ prem.
18 | $(R_{2-2}(p,a,b) \land \neg R_{2-2}(p,b,a)) \land (\neg R_{2-2}(p,b,c) \land R_{2-2}(p,c,b)) \land (R_{2-2}(p,a,c) \land \neg R_{2-2}(p,c,a)) \land R_{2-2}(p,a,a) \land R_{2-2}(p,b,b) \land R_{2-2}(p,c,c) \land (R_{2-2}(q,a,b) \land \neg R_{2-2}(q,b,a)) \land (\neg R_{2-2}(q,b,c) \land R_{2-2}(q,c,b)) \land (R_{2-2}(q,a,c) \land \neg R_{2-2}(q,c,a)) \land R_{2-2}(q,a,a) \land R_{2-2}(q,b,b) \land R_{2-2}(q,c,c)$ prem.
19 | $(R_{2-3}(p,a,b) \land \neg R_{2-3}(p,b,a)) \land (\neg R_{2-3}(p,b,c) \land R_{2-3}(p,c,b)) \land (R_{2-3}(p,a,c) \land \neg R_{2-3}(p,c,a)) \land R_{2-3}(p,a,a) \land R_{2-3}(p,b,b) \land R_{2-3}(p,c,c) \land (\neg R_{2-3}(q,a,b) \land R_{2-3}(q,b,a)) \land (R_{2-3}(q,b,c) \land \neg R_{2-3}(q,c,b)) \land (R_{2-3}(q,a,c) \land \neg R_{2-3}(q,c,a)) \land R_{2-3}(q,a,a) \land R_{2-3}(q,b,b) \land R_{2-3}(q,c,c)$ prem.
20 | $(R_{2-4}(p,a,b) \land \neg R_{2-4}(p,b,a)) \land (\neg R_{2-4}(p,b,c) \land R_{2-4}(p,c,b)) \land (R_{2-4}(p,a,c) \land \neg R_{2-4}(p,c,a)) \land R_{2-4}(p,a,a) \land R_{2-4}(p,b,b) \land R_{2-4}(p,c,c) \land (\neg R_{2-4}(q,a,b) \land R_{2-4}(q,b,a)) \land (R_{2-4}(q,b,c) \land \neg R_{2-4}(q,c,b)) \land (\neg R_{2-4}(q,a,c) \land R_{2-4}(q,c,a)) \land R_{2-4}(q,a,a) \land R_{2-4}(q,b,b) \land R_{2-4}(q,c,c)$ prem.
21 | $(R_{2-5}(p,a,b) \land \neg R_{2-5}(p,b,a)) \land (\neg R_{2-5}(p,b,c) \land R_{2-5}(p,c,b)) \land (R_{2-5}(p,a,c) \land \neg R_{2-5}(p,c,a)) \land R_{2-5}(p,a,a) \land R_{2-5}(p,b,b) \land R_{2-5}(p,c,c) \land (R_{2-5}(q,a,b) \land \neg R_{2-5}(q,b,a)) \land (\neg R_{2-5}(q,b,c) \land R_{2-5}(q,c,b)) \land (\neg R_{2-5}(q,a,c) \land R_{2-5}(q,c,a)) \land R_{2-5}(q,a,a) \land R_{2-5}(q,b,b) \land R_{2-5}(q,c,c)$ prem.
22 | $(R_{2-6}(p,a,b) \land \neg R_{2-6}(p,b,a)) \land (\neg R_{2-6}(p,b,c) \land R_{2-6}(p,c,b)) \land (R_{2-6}(p,a,c) \land \neg R_{2-6}(p,c,a)) \land R_{2-6}(p,a,a) \land R_{2-6}(p,b,b) \land R_{2-6}(p,c,c) \land (\neg R_{2-6}(q,a,b) \land R_{2-6}(q,b,a)) \land (\neg R_{2-6}(q,b,c) \land R_{2-6}(q,c,b)) \land (\neg R_{2-6}(q,a,c) \land R_{2-6}(q,c,a)) \land R_{2-6}(q,a,a) \land R_{2-6}(q,b,b) \land R_{2-6}(q,c,c)$ prem.
23 | $(R_{2-7}(p,a,b) \land \neg R_{2-7}(p,b,a)) \land (\neg R_{2-7}(p,b,c) \land R_{2-7}(p,c,b)) \land (R_{2-7}(p,a,c) \land \neg R_{2-7}(p,c,a)) \land R_{2-7}(p,a,a) \land R_{2-7}(p,b,b) \land R_{2-7}(p,c,c) \land (R_{2-7}(q,a,b) \land R_{2-7}(q,b,a)) \land (R_{2-7}(q,b,c) \land \neg R_{2-7}(q,c,b)) \land (R_{2-7}(q,a,c) \land \neg R_{2-7}(q,c,a)) \land R_{2-7}(q,a,a) \land R_{2-7}(q,b,b) \land R_{2-7}(q,c,c)$ prem.
24 | $(R_{2-8}(p,a,b) \land \neg R_{2-8}(p,b,a)) \land (\neg R_{2-8}(p,b,c) \land R_{2-8}(p,c,b)) \land (R_{2-8}(p,a,c) \land \neg R_{2-8}(p,c,a)) \land R_{2-8}(p,a,a) \land R_{2-8}(p,b,b) \land R_{2-8}(p,c,c) \land (R_{2-8}(q,a,b) \land R_{2-8}(q,b,a)) \land (\neg R_{2-8}(q,b,c) \land R_{2-8}(q,c,b)) \land (\neg R_{2-8}(q,a,c) \land R_{2-8}(q,c,a)) \land R_{2-8}(q,a,a) \land R_{2-8}(q,b,b) \land R_{2-8}(q,c,c)$ prem.
25 | $(R_{2-9}(p,a,b) \land \neg R_{2-9}(p,b,a)) \land (\neg R_{2-9}(p,b,c) \land R_{2-9}(p,c,b)) \land (R_{2-9}(p,a,c) \land \neg R_{2-9}(p,c,a)) \land R_{2-9}(p,a,a) \land R_{2-9}(p,b,b) \land R_{2-9}(p,c,c) \land (\neg R_{2-9}(q,a,b) \land R_{2-9}(q,b,a)) \land (R_{2-9}(q,b,c) \land R_{2-9}(q,c,b)) \land (\neg R_{2-9}(q,a,c) \land R_{2-9}(q,c,a)) \land R_{2-9}(q,a,a) \land R_{2-9}(q,b,b) \land R_{2-9}(q,c,c)$ prem.
26 | $(R_{2-10}(p,a,b) \land \neg R_{2-10}(p,b,a)) \land (\neg R_{2-10}(p,b,c) \land R_{2-10}(p,c,b)) \land (R_{2-10}(p,a,c) \land \neg R_{2-10}(p,c,a)) \land R_{2-10}(p,a,a) \land R_{2-10}(p,b,b) \land R_{2-10}(p,c,c) \land (R_{2-10}(q,a,b) \land \neg R_{2-10}(q,b,a)) \land (R_{2-10}(q,b,c) \land R_{2-10}(q,c,b)) \land (R_{2-10}(q,a,c) \land \neg R_{2-10}(q,c,a)) \land R_{2-10}(q,a,a) \land R_{2-10}(q,b,b) \land R_{2-10}(q,c,c)$ prem.
27 | $(R_{2-11}(p,a,b) \land \neg R_{2-11}(p,b,a)) \land (\neg R_{2-11}(p,b,c) \land R_{2-11}(p,c,b)) \land (R_{2-11}(p,a,c) \land \neg R_{2-11}(p,c,a)) \land R_{2-11}(p,a,a) \land R_{2-11}(p,b,b) \land R_{2-11}(p,c,c) \land (R_{2-11}(q,a,b) \land \neg R_{2-11}(q,b,a)) \land (\neg R_{2-11}(q,b,c) \land R_{2-11}(q,c,b)) \land (R_{2-11}(q,a,c) \land R_{2-11}(q,c,a)) \land R_{2-11}(q,a,a) \land R_{2-11}(q,b,b) \land R_{2-11}(q,c,c)$ prem.
28 | $(R_{2-12}(p,a,b) \land \neg R_{2-12}(p,b,a)) \land (\neg R_{2-12}(p,b,c) \land R_{2-12}(p,c,b)) \land (R_{2-12}(p,a,c) \land \neg R_{2-12}(p,c,a)) \land R_{2-12}(p,a,a) \land R_{2-12}(p,b,b) \land R_{2-12}(p,c,c) \land (\neg R_{2-12}(q,a,b) \land R_{2-12}(q,b,a)) \land (R_{2-12}(q,b,c) \land \neg R_{2-12}(q,c,b)) \land (R_{2-12}(q,a,c) \land R_{2-12}(q,c,a)) \land R_{2-12}(q,a,a) \land R_{2-12}(q,b,b) \land R_{2-12}(q,c,c)$ prem.
29 | $(R_{2-13}(p,a,b) \land \neg R_{2-13}(p,b,a)) \land (\neg R_{2-13}(p,b,c) \land R_{2-13}(p,c,b)) \land (R_{2-13}(p,a,c) \land \neg R_{2-13}(p,c,a)) \land R_{2-13}(p,a,a) \land R_{2-13}(p,b,b) \land R_{2-13}(p,c,c) \land (R_{2-13}(q,a,b) \land R_{2-13}(q,b,a)) \land (R_{2-13}(q,b,c) \land R_{2-13}(q,c,b)) \land (R_{2-13}(q,a,c) \land R_{2-13}(q,c,a)) \land R_{2-13}(q,a,a) \land R_{2-13}(q,b,b) \land R_{2-13}(q,c,c)$ prem.
30 | $(\neg R_{3-1}(p,a,b) \land R_{3-1}(p,b,a)) \land (R_{3-1}(p,b,c) \land \neg R_{3-1}(p,c,b)) \land (R_{3-1}(p,a,c) \land \neg R_{3-1}(p,c,a)) \land R_{3-1}(p,a,a) \land R_{3-1}(p,b,b) \land R_{3-1}(p,c,c) \land (R_{3-1}(q,a,b) \land \neg R_{3-1}(q,b,a)) \land (R_{3-1}(q,b,c) \land \neg R_{3-1}(q,c,b)) \land (R_{3-1}(q,a,c) \land \neg R_{3-1}(q,c,a)) \land R_{3-1}(q,a,a) \land R_{3-1}(q,b,b) \land R_{3-1}(q,c,c)$ prem.
31 | $(\neg R_{3-2}(p,a,b) \land R_{3-2}(p,b,a)) \land (R_{3-2}(p,b,c) \land \neg R_{3-2}(p,c,b)) \land (R_{3-2}(p,a,c) \land \neg R_{3-2}(p,c,a)) \land R_{3-2}(p,a,a) \land R_{3-2}(p,b,b) \land R_{3-2}(p,c,c) \land (R_{3-2}(q,a,b) \land \neg R_{3-2}(q,b,a)) \land (\neg R_{3-2}(q,b,c) \land R_{3-2}(q,c,b)) \land (R_{3-2}(q,a,c) \land \neg R_{3-2}(q,c,a)) \land R_{3-2}(q,a,a) \land R_{3-2}(q,b,b) \land R_{3-2}(q,c,c)$ prem.
32 | $(\neg R_{3-3}(p,a,b) \land R_{3-3}(p,b,a)) \land (R_{3-3}(p,b,c) \land \neg R_{3-3}(p,c,b)) \land (R_{3-3}(p,a,c) \land \neg R_{3-3}(p,c,a)) \land R_{3-3}(p,a,a) \land R_{3-3}(p,b,b) \land R_{3-3}(p,c,c) \land (\neg R_{3-3}(q,a,b) \land R_{3-3}(q,b,a)) \land (R_{3-3}(q,b,c) \land \neg R_{3-3}(q,c,b)) \land (R_{3-3}(q,a,c) \land \neg R_{3-3}(q,c,a)) \land R_{3-3}(q,a,a) \land R_{3-3}(q,b,b) \land R_{3-3}(q,c,c)$ prem.
33 | $(\neg R_{3-4}(p,a,b) \land R_{3-4}(p,b,a)) \land (R_{3-4}(p,b,c) \land \neg R_{3-4}(p,c,b)) \land (R_{3-4}(p,a,c) \land \neg R_{3-4}(p,c,a)) \land R_{3-4}(p,a,a) \land R_{3-4}(p,b,b) \land R_{3-4}(p,c,c) \land (\neg R_{3-4}(q,a,b) \land R_{3-4}(q,b,a)) \land (R_{3-4}(q,b,c) \land \neg R_{3-4}(q,c,b)) \land (\neg R_{3-4}(q,a,c) \land R_{3-4}(q,c,a)) \land R_{3-4}(q,a,a) \land R_{3-4}(q,b,b) \land R_{3-4}(q,c,c)$ prem.
34 | $(\neg R_{3-5}(p,a,b) \land R_{3-5}(p,b,a)) \land (R_{3-5}(p,b,c) \land \neg R_{3-5}(p,c,b)) \land (R_{3-5}(p,a,c) \land \neg R_{3-5}(p,c,a)) \land R_{3-5}(p,a,a) \land R_{3-5}(p,b,b) \land R_{3-5}(p,c,c) \land (R_{3-5}(q,a,b) \land \neg R_{3-5}(q,b,a)) \land (\neg R_{3-5}(q,b,c) \land R_{3-5}(q,c,b)) \land (\neg R_{3-5}(q,a,c) \land R_{3-5}(q,c,a)) \land R_{3-5}(q,a,a) \land R_{3-5}(q,b,b) \land R_{3-5}(q,c,c)$ prem.
35 | $(\neg R_{3-6}(p,a,b) \land R_{3-6}(p,b,a)) \land (R_{3-6}(p,b,c) \land \neg R_{3-6}(p,c,b)) \land (R_{3-6}(p,a,c) \land \neg R_{3-6}(p,c,a)) \land R_{3-6}(p,a,a) \land R_{3-6}(p,b,b) \land R_{3-6}(p,c,c) \land (\neg R_{3-6}(q,a,b) \land R_{3-6}(q,b,a)) \land (\neg R_{3-6}(q,b,c) \land R_{3-6}(q,c,b)) \land (\neg R_{3-6}(q,a,c) \land R_{3-6}(q,c,a)) \land R_{3-6}(q,a,a) \land R_{3-6}(q,b,b) \land R_{3-6}(q,c,c)$ prem.
36 | $(\neg R_{3-7}(p,a,b) \land R_{3-7}(p,b,a)) \land (R_{3-7}(p,b,c) \land \neg R_{3-7}(p,c,b)) \land (R_{3-7}(p,a,c) \land \neg R_{3-7}(p,c,a)) \land R_{3-7}(p,a,a) \land R_{3-7}(p,b,b) \land R_{3-7}(p,c,c) \land (R_{3-7}(q,a,b) \land R_{3-7}(q,b,a)) \land (R_{3-7}(q,b,c) \land \neg R_{3-7}(q,c,b)) \land (R_{3-7}(q,a,c) \land \neg R_{3-7}(q,c,a)) \land R_{3-7}(q,a,a) \land R_{3-7}(q,b,b) \land R_{3-7}(q,c,c)$ prem.
37 | $(\neg R_{3-8}(p,a,b) \land R_{3-8}(p,b,a)) \land (R_{3-8}(p,b,c) \land \neg R_{3-8}(p,c,b)) \land (R_{3-8}(p,a,c) \land \neg R_{3-8}(p,c,a)) \land R_{3-8}(p,a,a) \land R_{3-8}(p,b,b) \land R_{3-8}(p,c,c) \land (R_{3-8}(q,a,b) \land R_{3-8}(q,b,a)) \land (\neg R_{3-8}(q,b,c) \land R_{3-8}(q,c,b)) \land (\neg R_{3-8}(q,a,c) \land R_{3-8}(q,c,a)) \land R_{3-8}(q,a,a) \land R_{3-8}(q,b,b) \land R_{3-8}(q,c,c)$ prem.
38 | $(\neg R_{3-9}(p,a,b) \land R_{3-9}(p,b,a)) \land (R_{3-9}(p,b,c) \land \neg R_{3-9}(p,c,b)) \land (R_{3-9}(p,a,c) \land \neg R_{3-9}(p,c,a)) \land R_{3-9}(p,a,a) \land R_{3-9}(p,b,b) \land R_{3-9}(p,c,c) \land (\neg R_{3-9}(q,a,b) \land R_{3-9}(q,b,a)) \land (R_{3-9}(q,b,c) \land R_{3-9}(q,c,b)) \land (\neg R_{3-9}(q,a,c) \land R_{3-9}(q,c,a)) \land R_{3-9}(q,a,a) \land R_{3-9}(q,b,b) \land R_{3-9}(q,c,c)$ prem.
39 | $(\neg R_{3-10}(p,a,b) \land R_{3-10}(p,b,a)) \land (R_{3-10}(p,b,c) \land \neg R_{3-10}(p,c,b)) \land (R_{3-10}(p,a,c) \land \neg R_{3-10}(p,c,a)) \land R_{3-10}(p,a,a) \land R_{3-10}(p,b,b) \land R_{3-10}(p,c,c) \land (R_{3-10}(q,a,b) \land \neg R_{3-10}(q,b,a)) \land (R_{3-10}(q,b,c) \land R_{3-10}(q,c,b)) \land (R_{3-10}(q,a,c) \land \neg R_{3-10}(q,c,a)) \land R_{3-10}(q,a,a) \land R_{3-10}(q,b,b) \land R_{3-10}(q,c,c)$ prem.
40 | $(\neg R_{3-11}(p,a,b) \land R_{3-11}(p,b,a)) \land (R_{3-11}(p,b,c) \land \neg R_{3-11}(p,c,b)) \land (R_{3-11}(p,a,c) \land \neg R_{3-11}(p,c,a)) \land R_{3-11}(p,a,a) \land R_{3-11}(p,b,b) \land R_{3-11}(p,c,c) \land (R_{3-11}(q,a,b) \land \neg R_{3-11}(q,b,a)) \land (\neg R_{3-11}(q,b,c) \land R_{3-11}(q,c,b)) \land (R_{3-11}(q,a,c) \land R_{3-11}(q,c,a)) \land R_{3-11}(q,a,a) \land R_{3-11}(q,b,b) \land R_{3-11}(q,c,c)$ prem.
41 | $(\neg R_{3-12}(p,a,b) \land R_{3-12}(p,b,a)) \land (R_{3-12}(p,b,c) \land \neg R_{3-12}(p,c,b)) \land (R_{3-12}(p,a,c) \land \neg R_{3-12}(p,c,a)) \land R_{3-12}(p,a,a) \land R_{3-12}(p,b,b) \land R_{3-12}(p,c,c) \land (\neg R_{3-12}(q,a,b) \land R_{3-12}(q,b,a)) \land (R_{3-12}(q,b,c) \land \neg R_{3-12}(q,c,b)) \land (R_{3-12}(q,a,c) \land R_{3-12}(q,c,a)) \land R_{3-12}(q,a,a) \land R_{3-12}(q,b,b) \land R_{3-12}(q,c,c)$ prem.
42 | $(\neg R_{3-13}(p,a,b) \land R_{3-13}(p,b,a)) \land (R_{3-13}(p,b,c) \land \neg R_{3-13}(p,c,b)) \land (R_{3-13}(p,a,c) \land \neg R_{3-13}(p,c,a)) \land R_{3-13}(p,a,a) \land R_{3-13}(p,b,b) \land R_{3-13}(p,c,c) \land (R_{3-13}(q,a,b) \land R_{3-13}(q,b,a)) \land (R_{3-13}(q,b,c) \land R_{3-13}(q,c,b)) \land (R_{3-13}(q,a,c) \land R_{3-13}(q,c,a)) \land R_{3-13}(q,a,a) \land R_{3-13}(q,b,b) \land R_{3-13}(q,c,c)$ prem.
43 | $(\neg R_{4-1}(p,a,b) \land R_{4-1}(p,b,a)) \land (R_{4-1}(p,b,c) \land \neg R_{4-1}(p,c,b)) \land (\neg R_{4-1}(p,a,c) \land R_{4-1}(p,c,a)) \land R_{4-1}(p,a,a) \land R_{4-1}(p,b,b) \land R_{4-1}(p,c,c) \land (R_{4-1}(q,a,b) \land \neg R_{4-1}(q,b,a)) \land (R_{4-1}(q,b,c) \land \neg R_{4-1}(q,c,b)) \land (R_{4-1}(q,a,c) \land \neg R_{4-1}(q,c,a)) \land R_{4-1}(q,a,a) \land R_{4-1}(q,b,b) \land R_{4-1}(q,c,c)$ prem.
44 | $(\neg R_{4-2}(p,a,b) \land R_{4-2}(p,b,a)) \land (R_{4-2}(p,b,c) \land \neg R_{4-2}(p,c,b)) \land (\neg R_{4-2}(p,a,c) \land R_{4-2}(p,c,a)) \land R_{4-2}(p,a,a) \land R_{4-2}(p,b,b) \land R_{4-2}(p,c,c) \land (R_{4-2}(q,a,b) \land \neg R_{4-2}(q,b,a)) \land (\neg R_{4-2}(q,b,c) \land R_{4-2}(q,c,b)) \land (R_{4-2}(q,a,c) \land \neg R_{4-2}(q,c,a)) \land R_{4-2}(q,a,a) \land R_{4-2}(q,b,b) \land R_{4-2}(q,c,c)$ prem.
45 | $(\neg R_{4-3}(p,a,b) \land R_{4-3}(p,b,a)) \land (R_{4-3}(p,b,c) \land \neg R_{4-3}(p,c,b)) \land (\neg R_{4-3}(p,a,c) \land R_{4-3}(p,c,a)) \land R_{4-3}(p,a,a) \land R_{4-3}(p,b,b) \land R_{4-3}(p,c,c) \land (\neg R_{4-3}(q,a,b) \land R_{4-3}(q,b,a)) \land (R_{4-3}(q,b,c) \land \neg R_{4-3}(q,c,b)) \land (R_{4-3}(q,a,c) \land \neg R_{4-3}(q,c,a)) \land R_{4-3}(q,a,a) \land R_{4-3}(q,b,b) \land R_{4-3}(q,c,c)$ prem.
46 | $(\neg R_{4-4}(p,a,b) \land R_{4-4}(p,b,a)) \land (R_{4-4}(p,b,c) \land \neg R_{4-4}(p,c,b)) \land (\neg R_{4-4}(p,a,c) \land R_{4-4}(p,c,a)) \land R_{4-4}(p,a,a) \land R_{4-4}(p,b,b) \land R_{4-4}(p,c,c) \land (\neg R_{4-4}(q,a,b) \land R_{4-4}(q,b,a)) \land (R_{4-4}(q,b,c) \land \neg R_{4-4}(q,c,b)) \land (\neg R_{4-4}(q,a,c) \land R_{4-4}(q,c,a)) \land R_{4-4}(q,a,a) \land R_{4-4}(q,b,b) \land R_{4-4}(q,c,c)$ prem.
47 | $(\neg R_{4-5}(p,a,b) \land R_{4-5}(p,b,a)) \land (R_{4-5}(p,b,c) \land \neg R_{4-5}(p,c,b)) \land (\neg R_{4-5}(p,a,c) \land R_{4-5}(p,c,a)) \land R_{4-5}(p,a,a) \land R_{4-5}(p,b,b) \land R_{4-5}(p,c,c) \land (R_{4-5}(q,a,b) \land \neg R_{4-5}(q,b,a)) \land (\neg R_{4-5}(q,b,c) \land R_{4-5}(q,c,b)) \land (\neg R_{4-5}(q,a,c) \land R_{4-5}(q,c,a)) \land R_{4-5}(q,a,a) \land R_{4-5}(q,b,b) \land R_{4-5}(q,c,c)$ prem.
48 | $(\neg R_{4-6}(p,a,b) \land R_{4-6}(p,b,a)) \land (R_{4-6}(p,b,c) \land \neg R_{4-6}(p,c,b)) \land (\neg R_{4-6}(p,a,c) \land R_{4-6}(p,c,a)) \land R_{4-6}(p,a,a) \land R_{4-6}(p,b,b) \land R_{4-6}(p,c,c) \land (\neg R_{4-6}(q,a,b) \land R_{4-6}(q,b,a)) \land (\neg R_{4-6}(q,b,c) \land R_{4-6}(q,c,b)) \land (\neg R_{4-6}(q,a,c) \land R_{4-6}(q,c,a)) \land R_{4-6}(q,a,a) \land R_{4-6}(q,b,b) \land R_{4-6}(q,c,c)$ prem.
49 | $(\neg R_{4-7}(p,a,b) \land R_{4-7}(p,b,a)) \land (R_{4-7}(p,b,c) \land \neg R_{4-7}(p,c,b)) \land (\neg R_{4-7}(p,a,c) \land R_{4-7}(p,c,a)) \land R_{4-7}(p,a,a) \land R_{4-7}(p,b,b) \land R_{4-7}(p,c,c) \land (R_{4-7}(q,a,b) \land R_{4-7}(q,b,a)) \land (R_{4-7}(q,b,c) \land \neg R_{4-7}(q,c,b)) \land (R_{4-7}(q,a,c) \land \neg R_{4-7}(q,c,a)) \land R_{4-7}(q,a,a) \land R_{4-7}(q,b,b) \land R_{4-7}(q,c,c)$ prem.
50 | $(\neg R_{4-8}(p,a,b) \land R_{4-8}(p,b,a)) \land (R_{4-8}(p,b,c) \land \neg R_{4-8}(p,c,b)) \land (\neg R_{4-8}(p,a,c) \land R_{4-8}(p,c,a)) \land R_{4-8}(p,a,a) \land R_{4-8}(p,b,b) \land R_{4-8}(p,c,c) \land (R_{4-8}(q,a,b) \land R_{4-8}(q,b,a)) \land (\neg R_{4-8}(q,b,c) \land R_{4-8}(q,c,b)) \land (\neg R_{4-8}(q,a,c) \land R_{4-8}(q,c,a)) \land R_{4-8}(q,a,a) \land R_{4-8}(q,b,b) \land R_{4-8}(q,c,c)$ prem.
51 | $(\neg R_{4-9}(p,a,b) \land R_{4-9}(p,b,a)) \land (R_{4-9}(p,b,c) \land \neg R_{4-9}(p,c,b)) \land (\neg R_{4-9}(p,a,c) \land R_{4-9}(p,c,a)) \land R_{4-9}(p,a,a) \land R_{4-9}(p,b,b) \land R_{4-9}(p,c,c) \land (\neg R_{4-9}(q,a,b) \land R_{4-9}(q,b,a)) \land (R_{4-9}(q,b,c) \land R_{4-9}(q,c,b)) \land (\neg R_{4-9}(q,a,c) \land R_{4-9}(q,c,a)) \land R_{4-9}(q,a,a) \land R_{4-9}(q,b,b) \land R_{4-9}(q,c,c)$ prem.
52 | $(\neg R_{4-10}(p,a,b) \land R_{4-10}(p,b,a)) \land (R_{4-10}(p,b,c) \land \neg R_{4-10}(p,c,b)) \land (\neg R_{4-10}(p,a,c) \land R_{4-10}(p,c,a)) \land R_{4-10}(p,a,a) \land R_{4-10}(p,b,b) \land R_{4-10}(p,c,c) \land (R_{4-10}(q,a,b) \land \neg R_{4-10}(q,b,a)) \land (R_{4-10}(q,b,c) \land R_{4-10}(q,c,b)) \land (R_{4-10}(q,a,c) \land \neg R_{4-10}(q,c,a)) \land R_{4-10}(q,a,a) \land R_{4-10}(q,b,b) \land R_{4-10}(q,c,c)$ prem.
53 | $(\neg R_{4-11}(p,a,b) \land R_{4-11}(p,b,a)) \land (R_{4-11}(p,b,c) \land \neg R_{4-11}(p,c,b)) \land (\neg R_{4-11}(p,a,c) \land R_{4-11}(p,c,a)) \land R_{4-11}(p,a,a) \land R_{4-11}(p,b,b) \land R_{4-11}(p,c,c) \land (R_{4-11}(q,a,b) \land \neg R_{4-11}(q,b,a)) \land (\neg R_{4-11}(q,b,c) \land R_{4-11}(q,c,b)) \land (R_{4-11}(q,a,c) \land R_{4-11}(q,c,a)) \land R_{4-11}(q,a,a) \land R_{4-11}(q,b,b) \land R_{4-11}(q,c,c)$ prem.
54 | $(\neg R_{4-12}(p,a,b) \land R_{4-12}(p,b,a)) \land (R_{4-12}(p,b,c) \land \neg R_{4-12}(p,c,b)) \land (\neg R_{4-12}(p,a,c) \land R_{4-12}(p,c,a)) \land R_{4-12}(p,a,a) \land R_{4-12}(p,b,b) \land R_{4-12}(p,c,c) \land (\neg R_{4-12}(q,a,b) \land R_{4-12}(q,b,a)) \land (R_{4-12}(q,b,c) \land \neg R_{4-12}(q,c,b)) \land (R_{4-12}(q,a,c) \land R_{4-12}(q,c,a)) \land R_{4-12}(q,a,a) \land R_{4-12}(q,b,b) \land R_{4-12}(q,c,c)$ prem.
55 | $(\neg R_{4-13}(p,a,b) \land R_{4-13}(p,b,a)) \land (R_{4-13}(p,b,c) \land \neg R_{4-13}(p,c,b)) \land (\neg R_{4-13}(p,a,c) \land R_{4-13}(p,c,a)) \land R_{4-13}(p,a,a) \land R_{4-13}(p,b,b) \land R_{4-13}(p,c,c) \land (R_{4-13}(q,a,b) \land R_{4-13}(q,b,a)) \land (R_{4-13}(q,b,c) \land R_{4-13}(q,c,b)) \land (R_{4-13}(q,a,c) \land R_{4-13}(q,c,a)) \land R_{4-13}(q,a,a) \land R_{4-13}(q,b,b) \land R_{4-13}(q,c,c)$ prem.
56 | $(R_{5-1}(p,a,b) \land \neg R_{5-1}(p,b,a)) \land (\neg R_{5-1}(p,b,c) \land R_{5-1}(p,c,b)) \land (\neg R_{5-1}(p,a,c) \land R_{5-1}(p,c,a)) \land R_{5-1}(p,a,a) \land R_{5-1}(p,b,b) \land R_{5-1}(p,c,c) \land (R_{5-1}(q,a,b) \land \neg R_{5-1}(q,b,a)) \land (R_{5-1}(q,b,c) \land \neg R_{5-1}(q,c,b)) \land (R_{5-1}(q,a,c) \land \neg R_{5-1}(q,c,a)) \land R_{5-1}(q,a,a) \land R_{5-1}(q,b,b) \land R_{5-1}(q,c,c)$ prem.
57 | $(R_{5-2}(p,a,b) \land \neg R_{5-2}(p,b,a)) \land (\neg R_{5-2}(p,b,c) \land R_{5-2}(p,c,b)) \land (\neg R_{5-2}(p,a,c) \land R_{5-2}(p,c,a)) \land R_{5-2}(p,a,a) \land R_{5-2}(p,b,b) \land R_{5-2}(p,c,c) \land (R_{5-2}(q,a,b) \land \neg R_{5-2}(q,b,a)) \land (\neg R_{5-2}(q,b,c) \land R_{5-2}(q,c,b)) \land (R_{5-2}(q,a,c) \land \neg R_{5-2}(q,c,a)) \land R_{5-2}(q,a,a) \land R_{5-2}(q,b,b) \land R_{5-2}(q,c,c)$ prem.
58 | $(R_{5-3}(p,a,b) \land \neg R_{5-3}(p,b,a)) \land (\neg R_{5-3}(p,b,c) \land R_{5-3}(p,c,b)) \land (\neg R_{5-3}(p,a,c) \land R_{5-3}(p,c,a)) \land R_{5-3}(p,a,a) \land R_{5-3}(p,b,b) \land R_{5-3}(p,c,c) \land (\neg R_{5-3}(q,a,b) \land R_{5-3}(q,b,a)) \land (R_{5-3}(q,b,c) \land \neg R_{5-3}(q,c,b)) \land (R_{5-3}(q,a,c) \land \neg R_{5-3}(q,c,a)) \land R_{5-3}(q,a,a) \land R_{5-3}(q,b,b) \land R_{5-3}(q,c,c)$ prem.
59 | $(R_{5-4}(p,a,b) \land \neg R_{5-4}(p,b,a)) \land (\neg R_{5-4}(p,b,c) \land R_{5-4}(p,c,b)) \land (\neg R_{5-4}(p,a,c) \land R_{5-4}(p,c,a)) \land R_{5-4}(p,a,a) \land R_{5-4}(p,b,b) \land R_{5-4}(p,c,c) \land (\neg R_{5-4}(q,a,b) \land R_{5-4}(q,b,a)) \land (R_{5-4}(q,b,c) \land \neg R_{5-4}(q,c,b)) \land (\neg R_{5-4}(q,a,c) \land R_{5-4}(q,c,a)) \land R_{5-4}(q,a,a) \land R_{5-4}(q,b,b) \land R_{5-4}(q,c,c)$ prem.
60 | $(R_{5-5}(p,a,b) \land \neg R_{5-5}(p,b,a)) \land (\neg R_{5-5}(p,b,c) \land R_{5-5}(p,c,b)) \land (\neg R_{5-5}(p,a,c) \land R_{5-5}(p,c,a)) \land R_{5-5}(p,a,a) \land R_{5-5}(p,b,b) \land R_{5-5}(p,c,c) \land (R_{5-5}(q,a,b) \land \neg R_{5-5}(q,b,a)) \land (\neg R_{5-5}(q,b,c) \land R_{5-5}(q,c,b)) \land (\neg R_{5-5}(q,a,c) \land R_{5-5}(q,c,a)) \land R_{5-5}(q,a,a) \land R_{5-5}(q,b,b) \land R_{5-5}(q,c,c)$ prem.
61 | $(R_{5-6}(p,a,b) \land \neg R_{5-6}(p,b,a)) \land (\neg R_{5-6}(p,b,c) \land R_{5-6}(p,c,b)) \land (\neg R_{5-6}(p,a,c) \land R_{5-6}(p,c,a)) \land R_{5-6}(p,a,a) \land R_{5-6}(p,b,b) \land R_{5-6}(p,c,c) \land (\neg R_{5-6}(q,a,b) \land R_{5-6}(q,b,a)) \land (\neg R_{5-6}(q,b,c) \land R_{5-6}(q,c,b)) \land (\neg R_{5-6}(q,a,c) \land R_{5-6}(q,c,a)) \land R_{5-6}(q,a,a) \land R_{5-6}(q,b,b) \land R_{5-6}(q,c,c)$ prem.
62 | $(R_{5-7}(p,a,b) \land \neg R_{5-7}(p,b,a)) \land (\neg R_{5-7}(p,b,c) \land R_{5-7}(p,c,b)) \land (\neg R_{5-7}(p,a,c) \land R_{5-7}(p,c,a)) \land R_{5-7}(p,a,a) \land R_{5-7}(p,b,b) \land R_{5-7}(p,c,c) \land (R_{5-7}(q,a,b) \land R_{5-7}(q,b,a)) \land (R_{5-7}(q,b,c) \land \neg R_{5-7}(q,c,b)) \land (R_{5-7}(q,a,c) \land \neg R_{5-7}(q,c,a)) \land R_{5-7}(q,a,a) \land R_{5-7}(q,b,b) \land R_{5-7}(q,c,c)$ prem.
63 | $(R_{5-8}(p,a,b) \land \neg R_{5-8}(p,b,a)) \land (\neg R_{5-8}(p,b,c) \land R_{5-8}(p,c,b)) \land (\neg R_{5-8}(p,a,c) \land R_{5-8}(p,c,a)) \land R_{5-8}(p,a,a) \land R_{5-8}(p,b,b) \land R_{5-8}(p,c,c) \land (R_{5-8}(q,a,b) \land R_{5-8}(q,b,a)) \land (\neg R_{5-8}(q,b,c) \land R_{5-8}(q,c,b)) \land (\neg R_{5-8}(q,a,c) \land R_{5-8}(q,c,a)) \land R_{5-8}(q,a,a) \land R_{5-8}(q,b,b) \land R_{5-8}(q,c,c)$ prem.
64 | $(R_{5-9}(p,a,b) \land \neg R_{5-9}(p,b,a)) \land (\neg R_{5-9}(p,b,c) \land R_{5-9}(p,c,b)) \land (\neg R_{5-9}(p,a,c) \land R_{5-9}(p,c,a)) \land R_{5-9}(p,a,a) \land R_{5-9}(p,b,b) \land R_{5-9}(p,c,c) \land (\neg R_{5-9}(q,a,b) \land R_{5-9}(q,b,a)) \land (R_{5-9}(q,b,c) \land R_{5-9}(q,c,b)) \land (\neg R_{5-9}(q,a,c) \land R_{5-9}(q,c,a)) \land R_{5-9}(q,a,a) \land R_{5-9}(q,b,b) \land R_{5-9}(q,c,c)$ prem.
65 | $(R_{5-10}(p,a,b) \land \neg R_{5-10}(p,b,a)) \land (\neg R_{5-10}(p,b,c) \land R_{5-10}(p,c,b)) \land (\neg R_{5-10}(p,a,c) \land R_{5-10}(p,c,a)) \land R_{5-10}(p,a,a) \land R_{5-10}(p,b,b) \land R_{5-10}(p,c,c) \land (R_{5-10}(q,a,b) \land \neg R_{5-10}(q,b,a)) \land (R_{5-10}(q,b,c) \land R_{5-10}(q,c,b)) \land (R_{5-10}(q,a,c) \land \neg R_{5-10}(q,c,a)) \land R_{5-10}(q,a,a) \land R_{5-10}(q,b,b) \land R_{5-10}(q,c,c)$ prem.
66 | $(R_{5-11}(p,a,b) \land \neg R_{5-11}(p,b,a)) \land (\neg R_{5-11}(p,b,c) \land R_{5-11}(p,c,b)) \land (\neg R_{5-11}(p,a,c) \land R_{5-11}(p,c,a)) \land R_{5-11}(p,a,a) \land R_{5-11}(p,b,b) \land R_{5-11}(p,c,c) \land (R_{5-11}(q,a,b) \land \neg R_{5-11}(q,b,a)) \land (\neg R_{5-11}(q,b,c) \land R_{5-11}(q,c,b)) \land (R_{5-11}(q,a,c) \land R_{5-11}(q,c,a)) \land R_{5-11}(q,a,a) \land R_{5-11}(q,b,b) \land R_{5-11}(q,c,c)$ prem.
67 | $(R_{5-12}(p,a,b) \land \neg R_{5-12}(p,b,a)) \land (\neg R_{5-12}(p,b,c) \land R_{5-12}(p,c,b)) \land (\neg R_{5-12}(p,a,c) \land R_{5-12}(p,c,a)) \land R_{5-12}(p,a,a) \land R_{5-12}(p,b,b) \land R_{5-12}(p,c,c) \land (\neg R_{5-12}(q,a,b) \land R_{5-12}(q,b,a)) \land (R_{5-12}(q,b,c) \land \neg R_{5-12}(q,c,b)) \land (R_{5-12}(q,a,c) \land R_{5-12}(q,c,a)) \land R_{5-12}(q,a,a) \land R_{5-12}(q,b,b) \land R_{5-12}(q,c,c)$ prem.
68 | $(R_{5-13}(p,a,b) \land \neg R_{5-13}(p,b,a)) \land (\neg R_{5-13}(p,b,c) \land R_{5-13}(p,c,b)) \land (\neg R_{5-13}(p,a,c) \land R_{5-13}(p,c,a)) \land R_{5-13}(p,a,a) \land R_{5-13}(p,b,b) \land R_{5-13}(p,c,c) \land (R_{5-13}(q,a,b) \land R_{5-13}(q,b,a)) \land (R_{5-13}(q,b,c) \land R_{5-13}(q,c,b)) \land (R_{5-13}(q,a,c) \land R_{5-13}(q,c,a)) \land R_{5-13}(q,a,a) \land R_{5-13}(q,b,b) \land R_{5-13}(q,c,c)$ prem.
69 | $(\neg R_{6-1}(p,a,b) \land R_{6-1}(p,b,a)) \land (\neg R_{6-1}(p,b,c) \land R_{6-1}(p,c,b)) \land (\neg R_{6-1}(p,a,c) \land R_{6-1}(p,c,a)) \land R_{6-1}(p,a,a) \land R_{6-1}(p,b,b) \land R_{6-1}(p,c,c) \land (R_{6-1}(q,a,b) \land \neg R_{6-1}(q,b,a)) \land (R_{6-1}(q,b,c) \land \neg R_{6-1}(q,c,b)) \land (R_{6-1}(q,a,c) \land \neg R_{6-1}(q,c,a)) \land R_{6-1}(q,a,a) \land R_{6-1}(q,b,b) \land R_{6-1}(q,c,c)$ prem.
70 | $(\neg R_{6-2}(p,a,b) \land R_{6-2}(p,b,a)) \land (\neg R_{6-2}(p,b,c) \land R_{6-2}(p,c,b)) \land (\neg R_{6-2}(p,a,c) \land R_{6-2}(p,c,a)) \land R_{6-2}(p,a,a) \land R_{6-2}(p,b,b) \land R_{6-2}(p,c,c) \land (R_{6-2}(q,a,b) \land \neg R_{6-2}(q,b,a)) \land (\neg R_{6-2}(q,b,c) \land R_{6-2}(q,c,b)) \land (R_{6-2}(q,a,c) \land \neg R_{6-2}(q,c,a)) \land R_{6-2}(q,a,a) \land R_{6-2}(q,b,b) \land R_{6-2}(q,c,c)$ prem.
71 | $(\neg R_{6-3}(p,a,b) \land R_{6-3}(p,b,a)) \land (\neg R_{6-3}(p,b,c) \land R_{6-3}(p,c,b)) \land (\neg R_{6-3}(p,a,c) \land R_{6-3}(p,c,a)) \land R_{6-3}(p,a,a) \land R_{6-3}(p,b,b) \land R_{6-3}(p,c,c) \land (\neg R_{6-3}(q,a,b) \land R_{6-3}(q,b,a)) \land (R_{6-3}(q,b,c) \land \neg R_{6-3}(q,c,b)) \land (R_{6-3}(q,a,c) \land \neg R_{6-3}(q,c,a)) \land R_{6-3}(q,a,a) \land R_{6-3}(q,b,b) \land R_{6-3}(q,c,c)$ prem.
72 | $(\neg R_{6-4}(p,a,b) \land R_{6-4}(p,b,a)) \land (\neg R_{6-4}(p,b,c) \land R_{6-4}(p,c,b)) \land (\neg R_{6-4}(p,a,c) \land R_{6-4}(p,c,a)) \land R_{6-4}(p,a,a) \land R_{6-4}(p,b,b) \land R_{6-4}(p,c,c) \land (\neg R_{6-4}(q,a,b) \land R_{6-4}(q,b,a)) \land (R_{6-4}(q,b,c) \land \neg R_{6-4}(q,c,b)) \land (\neg R_{6-4}(q,a,c) \land R_{6-4}(q,c,a)) \land R_{6-4}(q,a,a) \land R_{6-4}(q,b,b) \land R_{6-4}(q,c,c)$ prem.
73 | $(\neg R_{6-5}(p,a,b) \land R_{6-5}(p,b,a)) \land (\neg R_{6-5}(p,b,c) \land R_{6-5}(p,c,b)) \land (\neg R_{6-5}(p,a,c) \land R_{6-5}(p,c,a)) \land R_{6-5}(p,a,a) \land R_{6-5}(p,b,b) \land R_{6-5}(p,c,c) \land (R_{6-5}(q,a,b) \land \neg R_{6-5}(q,b,a)) \land (\neg R_{6-5}(q,b,c) \land R_{6-5}(q,c,b)) \land (\neg R_{6-5}(q,a,c) \land R_{6-5}(q,c,a)) \land R_{6-5}(q,a,a) \land R_{6-5}(q,b,b) \land R_{6-5}(q,c,c)$ prem.
74 | $(\neg R_{6-6}(p,a,b) \land R_{6-6}(p,b,a)) \land (\neg R_{6-6}(p,b,c) \land R_{6-6}(p,c,b)) \land (\neg R_{6-6}(p,a,c) \land R_{6-6}(p,c,a)) \land R_{6-6}(p,a,a) \land R_{6-6}(p,b,b) \land R_{6-6}(p,c,c) \land (\neg R_{6-6}(q,a,b) \land R_{6-6}(q,b,a)) \land (\neg R_{6-6}(q,b,c) \land R_{6-6}(q,c,b)) \land (\neg R_{6-6}(q,a,c) \land R_{6-6}(q,c,a)) \land R_{6-6}(q,a,a) \land R_{6-6}(q,b,b) \land R_{6-6}(q,c,c)$ prem.
75 | $(\neg R_{6-7}(p,a,b) \land R_{6-7}(p,b,a)) \land (\neg R_{6-7}(p,b,c) \land R_{6-7}(p,c,b)) \land (\neg R_{6-7}(p,a,c) \land R_{6-7}(p,c,a)) \land R_{6-7}(p,a,a) \land R_{6-7}(p,b,b) \land R_{6-7}(p,c,c) \land (R_{6-7}(q,a,b) \land R_{6-7}(q,b,a)) \land (R_{6-7}(q,b,c) \land \neg R_{6-7}(q,c,b)) \land (R_{6-7}(q,a,c) \land \neg R_{6-7}(q,c,a)) \land R_{6-7}(q,a,a) \land R_{6-7}(q,b,b) \land R_{6-7}(q,c,c)$ prem.
76 | $(\neg R_{6-8}(p,a,b) \land R_{6-8}(p,b,a)) \land (\neg R_{6-8}(p,b,c) \land R_{6-8}(p,c,b)) \land (\neg R_{6-8}(p,a,c) \land R_{6-8}(p,c,a)) \land R_{6-8}(p,a,a) \land R_{6-8}(p,b,b) \land R_{6-8}(p,c,c) \land (R_{6-8}(q,a,b) \land R_{6-8}(q,b,a)) \land (\neg R_{6-8}(q,b,c) \land R_{6-8}(q,c,b)) \land (\neg R_{6-8}(q,a,c) \land R_{6-8}(q,c,a)) \land R_{6-8}(q,a,a) \land R_{6-8}(q,b,b) \land R_{6-8}(q,c,c)$ prem.
77 | $(\neg R_{6-9}(p,a,b) \land R_{6-9}(p,b,a)) \land (\neg R_{6-9}(p,b,c) \land R_{6-9}(p,c,b)) \land (\neg R_{6-9}(p,a,c) \land R_{6-9}(p,c,a)) \land R_{6-9}(p,a,a) \land R_{6-9}(p,b,b) \land R_{6-9}(p,c,c) \land (\neg R_{6-9}(q,a,b) \land R_{6-9}(q,b,a)) \land (R_{6-9}(q,b,c) \land R_{6-9}(q,c,b)) \land (\neg R_{6-9}(q,a,c) \land R_{6-9}(q,c,a)) \land R_{6-9}(q,a,a) \land R_{6-9}(q,b,b) \land R_{6-9}(q,c,c)$ prem.
78 | $(\neg R_{6-10}(p,a,b) \land R_{6-10}(p,b,a)) \land (\neg R_{6-10}(p,b,c) \land R_{6-10}(p,c,b)) \land (\neg R_{6-10}(p,a,c) \land R_{6-10}(p,c,a)) \land R_{6-10}(p,a,a) \land R_{6-10}(p,b,b) \land R_{6-10}(p,c,c) \land (R_{6-10}(q,a,b) \land \neg R_{6-10}(q,b,a)) \land (R_{6-10}(q,b,c) \land R_{6-10}(q,c,b)) \land (R_{6-10}(q,a,c) \land \neg R_{6-10}(q,c,a)) \land R_{6-10}(q,a,a) \land R_{6-10}(q,b,b) \land R_{6-10}(q,c,c)$ prem.
79 | $(\neg R_{6-11}(p,a,b) \land R_{6-11}(p,b,a)) \land (\neg R_{6-11}(p,b,c) \land R_{6-11}(p,c,b)) \land (\neg R_{6-11}(p,a,c) \land R_{6-11}(p,c,a)) \land R_{6-11}(p,a,a) \land R_{6-11}(p,b,b) \land R_{6-11}(p,c,c) \land (R_{6-11}(q,a,b) \land \neg R_{6-11}(q,b,a)) \land (\neg R_{6-11}(q,b,c) \land R_{6-11}(q,c,b)) \land (R_{6-11}(q,a,c) \land R_{6-11}(q,c,a)) \land R_{6-11}(q,a,a) \land R_{6-11}(q,b,b) \land R_{6-11}(q,c,c)$ prem.
80 | $(\neg R_{6-12}(p,a,b) \land R_{6-12}(p,b,a)) \land (\neg R_{6-12}(p,b,c) \land R_{6-12}(p,c,b)) \land (\neg R_{6-12}(p,a,c) \land R_{6-12}(p,c,a)) \land R_{6-12}(p,a,a) \land R_{6-12}(p,b,b) \land R_{6-12}(p,c,c) \land (\neg R_{6-12}(q,a,b) \land R_{6-12}(q,b,a)) \land (R_{6-12}(q,b,c) \land \neg R_{6-12}(q,c,b)) \land (R_{6-12}(q,a,c) \land R_{6-12}(q,c,a)) \land R_{6-12}(q,a,a) \land R_{6-12}(q,b,b) \land R_{6-12}(q,c,c)$ prem.
81 | $(\neg R_{6-13}(p,a,b) \land R_{6-13}(p,b,a)) \land (\neg R_{6-13}(p,b,c) \land R_{6-13}(p,c,b)) \land (\neg R_{6-13}(p,a,c) \land R_{6-13}(p,c,a)) \land R_{6-13}(p,a,a) \land R_{6-13}(p,b,b) \land R_{6-13}(p,c,c) \land (R_{6-13}(q,a,b) \land R_{6-13}(q,b,a)) \land (R_{6-13}(q,b,c) \land R_{6-13}(q,c,b)) \land (R_{6-13}(q,a,c) \land R_{6-13}(q,c,a)) \land R_{6-13}(q,a,a) \land R_{6-13}(q,b,b) \land R_{6-13}(q,c,c)$ prem.
82 | $(R_{7-1}(p,a,b) \land R_{7-1}(p,b,a)) \land (R_{7-1}(p,b,c) \land \neg R_{7-1}(p,c,b)) \land (R_{7-1}(p,a,c) \land \neg R_{7-1}(p,c,a)) \land R_{7-1}(p,a,a) \land R_{7-1}(p,b,b) \land R_{7-1}(p,c,c) \land (R_{7-1}(q,a,b) \land \neg R_{7-1}(q,b,a)) \land (R_{7-1}(q,b,c) \land \neg R_{7-1}(q,c,b)) \land (R_{7-1}(q,a,c) \land \neg R_{7-1}(q,c,a)) \land R_{7-1}(q,a,a) \land R_{7-1}(q,b,b) \land R_{7-1}(q,c,c)$ prem.
83 | $(R_{7-2}(p,a,b) \land R_{7-2}(p,b,a)) \land (R_{7-2}(p,b,c) \land \neg R_{7-2}(p,c,b)) \land (R_{7-2}(p,a,c) \land \neg R_{7-2}(p,c,a)) \land R_{7-2}(p,a,a) \land R_{7-2}(p,b,b) \land R_{7-2}(p,c,c) \land (R_{7-2}(q,a,b) \land \neg R_{7-2}(q,b,a)) \land (\neg R_{7-2}(q,b,c) \land R_{7-2}(q,c,b)) \land (R_{7-2}(q,a,c) \land \neg R_{7-2}(q,c,a)) \land R_{7-2}(q,a,a) \land R_{7-2}(q,b,b) \land R_{7-2}(q,c,c)$ prem.
84 | $(R_{7-3}(p,a,b) \land R_{7-3}(p,b,a)) \land (R_{7-3}(p,b,c) \land \neg R_{7-3}(p,c,b)) \land (R_{7-3}(p,a,c) \land \neg R_{7-3}(p,c,a)) \land R_{7-3}(p,a,a) \land R_{7-3}(p,b,b) \land R_{7-3}(p,c,c) \land (\neg R_{7-3}(q,a,b) \land R_{7-3}(q,b,a)) \land (R_{7-3}(q,b,c) \land \neg R_{7-3}(q,c,b)) \land (R_{7-3}(q,a,c) \land \neg R_{7-3}(q,c,a)) \land R_{7-3}(q,a,a) \land R_{7-3}(q,b,b) \land R_{7-3}(q,c,c)$ prem.
85 | $(R_{7-4}(p,a,b) \land R_{7-4}(p,b,a)) \land (R_{7-4}(p,b,c) \land \neg R_{7-4}(p,c,b)) \land (R_{7-4}(p,a,c) \land \neg R_{7-4}(p,c,a)) \land R_{7-4}(p,a,a) \land R_{7-4}(p,b,b) \land R_{7-4}(p,c,c) \land (\neg R_{7-4}(q,a,b) \land R_{7-4}(q,b,a)) \land (R_{7-4}(q,b,c) \land \neg R_{7-4}(q,c,b)) \land (\neg R_{7-4}(q,a,c) \land R_{7-4}(q,c,a)) \land R_{7-4}(q,a,a) \land R_{7-4}(q,b,b) \land R_{7-4}(q,c,c)$ prem.
86 | $(R_{7-5}(p,a,b) \land R_{7-5}(p,b,a)) \land (R_{7-5}(p,b,c) \land \neg R_{7-5}(p,c,b)) \land (R_{7-5}(p,a,c) \land \neg R_{7-5}(p,c,a)) \land R_{7-5}(p,a,a) \land R_{7-5}(p,b,b) \land R_{7-5}(p,c,c) \land (R_{7-5}(q,a,b) \land \neg R_{7-5}(q,b,a)) \land (\neg R_{7-5}(q,b,c) \land R_{7-5}(q,c,b)) \land (\neg R_{7-5}(q,a,c) \land R_{7-5}(q,c,a)) \land R_{7-5}(q,a,a) \land R_{7-5}(q,b,b) \land R_{7-5}(q,c,c)$ prem.
87 | $(R_{7-6}(p,a,b) \land R_{7-6}(p,b,a)) \land (R_{7-6}(p,b,c) \land \neg R_{7-6}(p,c,b)) \land (R_{7-6}(p,a,c) \land \neg R_{7-6}(p,c,a)) \land R_{7-6}(p,a,a) \land R_{7-6}(p,b,b) \land R_{7-6}(p,c,c) \land (\neg R_{7-6}(q,a,b) \land R_{7-6}(q,b,a)) \land (\neg R_{7-6}(q,b,c) \land R_{7-6}(q,c,b)) \land (\neg R_{7-6}(q,a,c) \land R_{7-6}(q,c,a)) \land R_{7-6}(q,a,a) \land R_{7-6}(q,b,b) \land R_{7-6}(q,c,c)$ prem.
88 | $(R_{7-7}(p,a,b) \land R_{7-7}(p,b,a)) \land (R_{7-7}(p,b,c) \land \neg R_{7-7}(p,c,b)) \land (R_{7-7}(p,a,c) \land \neg R_{7-7}(p,c,a)) \land R_{7-7}(p,a,a) \land R_{7-7}(p,b,b) \land R_{7-7}(p,c,c) \land (R_{7-7}(q,a,b) \land R_{7-7}(q,b,a)) \land (R_{7-7}(q,b,c) \land \neg R_{7-7}(q,c,b)) \land (R_{7-7}(q,a,c) \land \neg R_{7-7}(q,c,a)) \land R_{7-7}(q,a,a) \land R_{7-7}(q,b,b) \land R_{7-7}(q,c,c)$ prem.
89 | $(R_{7-8}(p,a,b) \land R_{7-8}(p,b,a)) \land (R_{7-8}(p,b,c) \land \neg R_{7-8}(p,c,b)) \land (R_{7-8}(p,a,c) \land \neg R_{7-8}(p,c,a)) \land R_{7-8}(p,a,a) \land R_{7-8}(p,b,b) \land R_{7-8}(p,c,c) \land (R_{7-8}(q,a,b) \land R_{7-8}(q,b,a)) \land (\neg R_{7-8}(q,b,c) \land R_{7-8}(q,c,b)) \land (\neg R_{7-8}(q,a,c) \land R_{7-8}(q,c,a)) \land R_{7-8}(q,a,a) \land R_{7-8}(q,b,b) \land R_{7-8}(q,c,c)$ prem.
90 | $(R_{7-9}(p,a,b) \land R_{7-9}(p,b,a)) \land (R_{7-9}(p,b,c) \land \neg R_{7-9}(p,c,b)) \land (R_{7-9}(p,a,c) \land \neg R_{7-9}(p,c,a)) \land R_{7-9}(p,a,a) \land R_{7-9}(p,b,b) \land R_{7-9}(p,c,c) \land (\neg R_{7-9}(q,a,b) \land R_{7-9}(q,b,a)) \land (R_{7-9}(q,b,c) \land R_{7-9}(q,c,b)) \land (\neg R_{7-9}(q,a,c) \land R_{7-9}(q,c,a)) \land R_{7-9}(q,a,a) \land R_{7-9}(q,b,b) \land R_{7-9}(q,c,c)$ prem.
91 | $(R_{7-10}(p,a,b) \land R_{7-10}(p,b,a)) \land (R_{7-10}(p,b,c) \land \neg R_{7-10}(p,c,b)) \land (R_{7-10}(p,a,c) \land \neg R_{7-10}(p,c,a)) \land R_{7-10}(p,a,a) \land R_{7-10}(p,b,b) \land R_{7-10}(p,c,c) \land (R_{7-10}(q,a,b) \land \neg R_{7-10}(q,b,a)) \land (R_{7-10}(q,b,c) \land R_{7-10}(q,c,b)) \land (R_{7-10}(q,a,c) \land \neg R_{7-10}(q,c,a)) \land R_{7-10}(q,a,a) \land R_{7-10}(q,b,b) \land R_{7-10}(q,c,c)$ prem.
92 | $(R_{7-11}(p,a,b) \land R_{7-11}(p,b,a)) \land (R_{7-11}(p,b,c) \land \neg R_{7-11}(p,c,b)) \land (R_{7-11}(p,a,c) \land \neg R_{7-11}(p,c,a)) \land R_{7-11}(p,a,a) \land R_{7-11}(p,b,b) \land R_{7-11}(p,c,c) \land (R_{7-11}(q,a,b) \land \neg R_{7-11}(q,b,a)) \land (\neg R_{7-11}(q,b,c) \land R_{7-11}(q,c,b)) \land (R_{7-11}(q,a,c) \land R_{7-11}(q,c,a)) \land R_{7-11}(q,a,a) \land R_{7-11}(q,b,b) \land R_{7-11}(q,c,c)$ prem.
93 | $(R_{7-12}(p,a,b) \land R_{7-12}(p,b,a)) \land (R_{7-12}(p,b,c) \land \neg R_{7-12}(p,c,b)) \land (R_{7-12}(p,a,c) \land \neg R_{7-12}(p,c,a)) \land R_{7-12}(p,a,a) \land R_{7-12}(p,b,b) \land R_{7-12}(p,c,c) \land (\neg R_{7-12}(q,a,b) \land R_{7-12}(q,b,a)) \land (R_{7-12}(q,b,c) \land \neg R_{7-12}(q,c,b)) \land (R_{7-12}(q,a,c) \land R_{7-12}(q,c,a)) \land R_{7-12}(q,a,a) \land R_{7-12}(q,b,b) \land R_{7-12}(q,c,c)$ prem.
94 | $(R_{7-13}(p,a,b) \land R_{7-13}(p,b,a)) \land (R_{7-13}(p,b,c) \land \neg R_{7-13}(p,c,b)) \land (R_{7-13}(p,a,c) \land \neg R_{7-13}(p,c,a)) \land R_{7-13}(p,a,a) \land R_{7-13}(p,b,b) \land R_{7-13}(p,c,c) \land (R_{7-13}(q,a,b) \land R_{7-13}(q,b,a)) \land (R_{7-13}(q,b,c) \land R_{7-13}(q,c,b)) \land (R_{7-13}(q,a,c) \land R_{7-13}(q,c,a)) \land R_{7-13}(q,a,a) \land R_{7-13}(q,b,b) \land R_{7-13}(q,c,c)$ prem.
95 | $(R_{8-1}(p,a,b) \land R_{8-1}(p,b,a)) \land (\neg R_{8-1}(p,b,c) \land R_{8-1}(p,c,b)) \land (\neg R_{8-1}(p,a,c) \land R_{8-1}(p,c,a)) \land R_{8-1}(p,a,a) \land R_{8-1}(p,b,b) \land R_{8-1}(p,c,c) \land (R_{8-1}(q,a,b) \land \neg R_{8-1}(q,b,a)) \land (R_{8-1}(q,b,c) \land \neg R_{8-1}(q,c,b)) \land (R_{8-1}(q,a,c) \land \neg R_{8-1}(q,c,a)) \land R_{8-1}(q,a,a) \land R_{8-1}(q,b,b) \land R_{8-1}(q,c,c)$ prem.
96 | $(R_{8-2}(p,a,b) \land R_{8-2}(p,b,a)) \land (\neg R_{8-2}(p,b,c) \land R_{8-2}(p,c,b)) \land (\neg R_{8-2}(p,a,c) \land R_{8-2}(p,c,a)) \land R_{8-2}(p,a,a) \land R_{8-2}(p,b,b) \land R_{8-2}(p,c,c) \land (R_{8-2}(q,a,b) \land \neg R_{8-2}(q,b,a)) \land (\neg R_{8-2}(q,b,c) \land R_{8-2}(q,c,b)) \land (R_{8-2}(q,a,c) \land \neg R_{8-2}(q,c,a)) \land R_{8-2}(q,a,a) \land R_{8-2}(q,b,b) \land R_{8-2}(q,c,c)$ prem.
97 | $(R_{8-3}(p,a,b) \land R_{8-3}(p,b,a)) \land (\neg R_{8-3}(p,b,c) \land R_{8-3}(p,c,b)) \land (\neg R_{8-3}(p,a,c) \land R_{8-3}(p,c,a)) \land R_{8-3}(p,a,a) \land R_{8-3}(p,b,b) \land R_{8-3}(p,c,c) \land (\neg R_{8-3}(q,a,b) \land R_{8-3}(q,b,a)) \land (R_{8-3}(q,b,c) \land \neg R_{8-3}(q,c,b)) \land (R_{8-3}(q,a,c) \land \neg R_{8-3}(q,c,a)) \land R_{8-3}(q,a,a) \land R_{8-3}(q,b,b) \land R_{8-3}(q,c,c)$ prem.
98 | $(R_{8-4}(p,a,b) \land R_{8-4}(p,b,a)) \land (\neg R_{8-4}(p,b,c) \land R_{8-4}(p,c,b)) \land (\neg R_{8-4}(p,a,c) \land R_{8-4}(p,c,a)) \land R_{8-4}(p,a,a) \land R_{8-4}(p,b,b) \land R_{8-4}(p,c,c) \land (\neg R_{8-4}(q,a,b) \land R_{8-4}(q,b,a)) \land (R_{8-4}(q,b,c) \land \neg R_{8-4}(q,c,b)) \land (\neg R_{8-4}(q,a,c) \land R_{8-4}(q,c,a)) \land R_{8-4}(q,a,a) \land R_{8-4}(q,b,b) \land R_{8-4}(q,c,c)$ prem.
99 | $(R_{8-5}(p,a,b) \land R_{8-5}(p,b,a)) \land (\neg R_{8-5}(p,b,c) \land R_{8-5}(p,c,b)) \land (\neg R_{8-5}(p,a,c) \land R_{8-5}(p,c,a)) \land R_{8-5}(p,a,a) \land R_{8-5}(p,b,b) \land R_{8-5}(p,c,c) \land (R_{8-5}(q,a,b) \land \neg R_{8-5}(q,b,a)) \land (\neg R_{8-5}(q,b,c) \land R_{8-5}(q,c,b)) \land (\neg R_{8-5}(q,a,c) \land R_{8-5}(q,c,a)) \land R_{8-5}(q,a,a) \land R_{8-5}(q,b,b) \land R_{8-5}(q,c,c)$ prem.
100 | $(R_{8-6}(p,a,b) \land R_{8-6}(p,b,a)) \land (\neg R_{8-6}(p,b,c) \land R_{8-6}(p,c,b)) \land (\neg R_{8-6}(p,a,c) \land R_{8-6}(p,c,a)) \land R_{8-6}(p,a,a) \land R_{8-6}(p,b,b) \land R_{8-6}(p,c,c) \land (\neg R_{8-6}(q,a,b) \land R_{8-6}(q,b,a)) \land (\neg R_{8-6}(q,b,c) \land R_{8-6}(q,c,b)) \land (\neg R_{8-6}(q,a,c) \land R_{8-6}(q,c,a)) \land R_{8-6}(q,a,a) \land R_{8-6}(q,b,b) \land R_{8-6}(q,c,c)$ prem.
101 | $(R_{8-7}(p,a,b) \land R_{8-7}(p,b,a)) \land (\neg R_{8-7}(p,b,c) \land R_{8-7}(p,c,b)) \land (\neg R_{8-7}(p,a,c) \land R_{8-7}(p,c,a)) \land R_{8-7}(p,a,a) \land R_{8-7}(p,b,b) \land R_{8-7}(p,c,c) \land (R_{8-7}(q,a,b) \land R_{8-7}(q,b,a)) \land (R_{8-7}(q,b,c) \land \neg R_{8-7}(q,c,b)) \land (R_{8-7}(q,a,c) \land \neg R_{8-7}(q,c,a)) \land R_{8-7}(q,a,a) \land R_{8-7}(q,b,b) \land R_{8-7}(q,c,c)$ prem.
102 | $(R_{8-8}(p,a,b) \land R_{8-8}(p,b,a)) \land (\neg R_{8-8}(p,b,c) \land R_{8-8}(p,c,b)) \land (\neg R_{8-8}(p,a,c) \land R_{8-8}(p,c,a)) \land R_{8-8}(p,a,a) \land R_{8-8}(p,b,b) \land R_{8-8}(p,c,c) \land (R_{8-8}(q,a,b) \land R_{8-8}(q,b,a)) \land (\neg R_{8-8}(q,b,c) \land R_{8-8}(q,c,b)) \land (\neg R_{8-8}(q,a,c) \land R_{8-8}(q,c,a)) \land R_{8-8}(q,a,a) \land R_{8-8}(q,b,b) \land R_{8-8}(q,c,c)$ prem.
103 | $(R_{8-9}(p,a,b) \land R_{8-9}(p,b,a)) \land (\neg R_{8-9}(p,b,c) \land R_{8-9}(p,c,b)) \land (\neg R_{8-9}(p,a,c) \land R_{8-9}(p,c,a)) \land R_{8-9}(p,a,a) \land R_{8-9}(p,b,b) \land R_{8-9}(p,c,c) \land (\neg R_{8-9}(q,a,b) \land R_{8-9}(q,b,a)) \land (R_{8-9}(q,b,c) \land R_{8-9}(q,c,b)) \land (\neg R_{8-9}(q,a,c) \land R_{8-9}(q,c,a)) \land R_{8-9}(q,a,a) \land R_{8-9}(q,b,b) \land R_{8-9}(q,c,c)$ prem.
104 | $(R_{8-10}(p,a,b) \land R_{8-10}(p,b,a)) \land (\neg R_{8-10}(p,b,c) \land R_{8-10}(p,c,b)) \land (\neg R_{8-10}(p,a,c) \land R_{8-10}(p,c,a)) \land R_{8-10}(p,a,a) \land R_{8-10}(p,b,b) \land R_{8-10}(p,c,c) \land (R_{8-10}(q,a,b) \land \neg R_{8-10}(q,b,a)) \land (R_{8-10}(q,b,c) \land R_{8-10}(q,c,b)) \land (R_{8-10}(q,a,c) \land \neg R_{8-10}(q,c,a)) \land R_{8-10}(q,a,a) \land R_{8-10}(q,b,b) \land R_{8-10}(q,c,c)$ prem.
105 | $(R_{8-11}(p,a,b) \land R_{8-11}(p,b,a)) \land (\neg R_{8-11}(p,b,c) \land R_{8-11}(p,c,b)) \land (\neg R_{8-11}(p,a,c) \land R_{8-11}(p,c,a)) \land R_{8-11}(p,a,a) \land R_{8-11}(p,b,b) \land R_{8-11}(p,c,c) \land (R_{8-11}(q,a,b) \land \neg R_{8-11}(q,b,a)) \land (\neg R_{8-11}(q,b,c) \land R_{8-11}(q,c,b)) \land (R_{8-11}(q,a,c) \land R_{8-11}(q,c,a)) \land R_{8-11}(q,a,a) \land R_{8-11}(q,b,b) \land R_{8-11}(q,c,c)$ prem.
106 | $(R_{8-12}(p,a,b) \land R_{8-12}(p,b,a)) \land (\neg R_{8-12}(p,b,c) \land R_{8-12}(p,c,b)) \land (\neg R_{8-12}(p,a,c) \land R_{8-12}(p,c,a)) \land R_{8-12}(p,a,a) \land R_{8-12}(p,b,b) \land R_{8-12}(p,c,c) \land (\neg R_{8-12}(q,a,b) \land R_{8-12}(q,b,a)) \land (R_{8-12}(q,b,c) \land \neg R_{8-12}(q,c,b)) \land (R_{8-12}(q,a,c) \land R_{8-12}(q,c,a)) \land R_{8-12}(q,a,a) \land R_{8-12}(q,b,b) \land R_{8-12}(q,c,c)$ prem.
107 | $(R_{8-13}(p,a,b) \land R_{8-13}(p,b,a)) \land (\neg R_{8-13}(p,b,c) \land R_{8-13}(p,c,b)) \land (\neg R_{8-13}(p,a,c) \land R_{8-13}(p,c,a)) \land R_{8-13}(p,a,a) \land R_{8-13}(p,b,b) \land R_{8-13}(p,c,c) \land (R_{8-13}(q,a,b) \land R_{8-13}(q,b,a)) \land (R_{8-13}(q,b,c) \land R_{8-13}(q,c,b)) \land (R_{8-13}(q,a,c) \land R_{8-13}(q,c,a)) \land R_{8-13}(q,a,a) \land R_{8-13}(q,b,b) \land R_{8-13}(q,c,c)$ prem.
108 | $(\neg R_{9-1}(p,a,b) \land R_{9-1}(p,b,a)) \land (R_{9-1}(p,b,c) \land R_{9-1}(p,c,b)) \land (\neg R_{9-1}(p,a,c) \land R_{9-1}(p,c,a)) \land R_{9-1}(p,a,a) \land R_{9-1}(p,b,b) \land R_{9-1}(p,c,c) \land (R_{9-1}(q,a,b) \land \neg R_{9-1}(q,b,a)) \land (R_{9-1}(q,b,c) \land \neg R_{9-1}(q,c,b)) \land (R_{9-1}(q,a,c) \land \neg R_{9-1}(q,c,a)) \land R_{9-1}(q,a,a) \land R_{9-1}(q,b,b) \land R_{9-1}(q,c,c)$ prem.
109 | $(\neg R_{9-2}(p,a,b) \land R_{9-2}(p,b,a)) \land (R_{9-2}(p,b,c) \land R_{9-2}(p,c,b)) \land (\neg R_{9-2}(p,a,c) \land R_{9-2}(p,c,a)) \land R_{9-2}(p,a,a) \land R_{9-2}(p,b,b) \land R_{9-2}(p,c,c) \land (R_{9-2}(q,a,b) \land \neg R_{9-2}(q,b,a)) \land (\neg R_{9-2}(q,b,c) \land R_{9-2}(q,c,b)) \land (R_{9-2}(q,a,c) \land \neg R_{9-2}(q,c,a)) \land R_{9-2}(q,a,a) \land R_{9-2}(q,b,b) \land R_{9-2}(q,c,c)$ prem.
110 | $(\neg R_{9-3}(p,a,b) \land R_{9-3}(p,b,a)) \land (R_{9-3}(p,b,c) \land R_{9-3}(p,c,b)) \land (\neg R_{9-3}(p,a,c) \land R_{9-3}(p,c,a)) \land R_{9-3}(p,a,a) \land R_{9-3}(p,b,b) \land R_{9-3}(p,c,c) \land (\neg R_{9-3}(q,a,b) \land R_{9-3}(q,b,a)) \land (R_{9-3}(q,b,c) \land \neg R_{9-3}(q,c,b)) \land (R_{9-3}(q,a,c) \land \neg R_{9-3}(q,c,a)) \land R_{9-3}(q,a,a) \land R_{9-3}(q,b,b) \land R_{9-3}(q,c,c)$ prem.
111 | $(\neg R_{9-4}(p,a,b) \land R_{9-4}(p,b,a)) \land (R_{9-4}(p,b,c) \land R_{9-4}(p,c,b)) \land (\neg R_{9-4}(p,a,c) \land R_{9-4}(p,c,a)) \land R_{9-4}(p,a,a) \land R_{9-4}(p,b,b) \land R_{9-4}(p,c,c) \land (\neg R_{9-4}(q,a,b) \land R_{9-4}(q,b,a)) \land (R_{9-4}(q,b,c) \land \neg R_{9-4}(q,c,b)) \land (\neg R_{9-4}(q,a,c) \land R_{9-4}(q,c,a)) \land R_{9-4}(q,a,a) \land R_{9-4}(q,b,b) \land R_{9-4}(q,c,c)$ prem.
112 | $(\neg R_{9-5}(p,a,b) \land R_{9-5}(p,b,a)) \land (R_{9-5}(p,b,c) \land R_{9-5}(p,c,b)) \land (\neg R_{9-5}(p,a,c) \land R_{9-5}(p,c,a)) \land R_{9-5}(p,a,a) \land R_{9-5}(p,b,b) \land R_{9-5}(p,c,c) \land (R_{9-5}(q,a,b) \land \neg R_{9-5}(q,b,a)) \land (\neg R_{9-5}(q,b,c) \land R_{9-5}(q,c,b)) \land (\neg R_{9-5}(q,a,c) \land R_{9-5}(q,c,a)) \land R_{9-5}(q,a,a) \land R_{9-5}(q,b,b) \land R_{9-5}(q,c,c)$ prem.
113 | $(\neg R_{9-6}(p,a,b) \land R_{9-6}(p,b,a)) \land (R_{9-6}(p,b,c) \land R_{9-6}(p,c,b)) \land (\neg R_{9-6}(p,a,c) \land R_{9-6}(p,c,a)) \land R_{9-6}(p,a,a) \land R_{9-6}(p,b,b) \land R_{9-6}(p,c,c) \land (\neg R_{9-6}(q,a,b) \land R_{9-6}(q,b,a)) \land (\neg R_{9-6}(q,b,c) \land R_{9-6}(q,c,b)) \land (\neg R_{9-6}(q,a,c) \land R_{9-6}(q,c,a)) \land R_{9-6}(q,a,a) \land R_{9-6}(q,b,b) \land R_{9-6}(q,c,c)$ prem.
114 | $(\neg R_{9-7}(p,a,b) \land R_{9-7}(p,b,a)) \land (R_{9-7}(p,b,c) \land R_{9-7}(p,c,b)) \land (\neg R_{9-7}(p,a,c) \land R_{9-7}(p,c,a)) \land R_{9-7}(p,a,a) \land R_{9-7}(p,b,b) \land R_{9-7}(p,c,c) \land (R_{9-7}(q,a,b) \land R_{9-7}(q,b,a)) \land (R_{9-7}(q,b,c) \land \neg R_{9-7}(q,c,b)) \land (R_{9-7}(q,a,c) \land \neg R_{9-7}(q,c,a)) \land R_{9-7}(q,a,a) \land R_{9-7}(q,b,b) \land R_{9-7}(q,c,c)$ prem.
115 | $(\neg R_{9-8}(p,a,b) \land R_{9-8}(p,b,a)) \land (R_{9-8}(p,b,c) \land R_{9-8}(p,c,b)) \land (\neg R_{9-8}(p,a,c) \land R_{9-8}(p,c,a)) \land R_{9-8}(p,a,a) \land R_{9-8}(p,b,b) \land R_{9-8}(p,c,c) \land (R_{9-8}(q,a,b) \land R_{9-8}(q,b,a)) \land (\neg R_{9-8}(q,b,c) \land R_{9-8}(q,c,b)) \land (\neg R_{9-8}(q,a,c) \land R_{9-8}(q,c,a)) \land R_{9-8}(q,a,a) \land R_{9-8}(q,b,b) \land R_{9-8}(q,c,c)$ prem.
116 | $(\neg R_{9-9}(p,a,b) \land R_{9-9}(p,b,a)) \land (R_{9-9}(p,b,c) \land R_{9-9}(p,c,b)) \land (\neg R_{9-9}(p,a,c) \land R_{9-9}(p,c,a)) \land R_{9-9}(p,a,a) \land R_{9-9}(p,b,b) \land R_{9-9}(p,c,c) \land (\neg R_{9-9}(q,a,b) \land R_{9-9}(q,b,a)) \land (R_{9-9}(q,b,c) \land R_{9-9}(q,c,b)) \land (\neg R_{9-9}(q,a,c) \land R_{9-9}(q,c,a)) \land R_{9-9}(q,a,a) \land R_{9-9}(q,b,b) \land R_{9-9}(q,c,c)$ prem.
117 | $(\neg R_{9-10}(p,a,b) \land R_{9-10}(p,b,a)) \land (R_{9-10}(p,b,c) \land R_{9-10}(p,c,b)) \land (\neg R_{9-10}(p,a,c) \land R_{9-10}(p,c,a)) \land R_{9-10}(p,a,a) \land R_{9-10}(p,b,b) \land R_{9-10}(p,c,c) \land (R_{9-10}(q,a,b) \land \neg R_{9-10}(q,b,a)) \land (R_{9-10}(q,b,c) \land R_{9-10}(q,c,b)) \land (R_{9-10}(q,a,c) \land \neg R_{9-10}(q,c,a)) \land R_{9-10}(q,a,a) \land R_{9-10}(q,b,b) \land R_{9-10}(q,c,c)$ prem.
118 | $(\neg R_{9-11}(p,a,b) \land R_{9-11}(p,b,a)) \land (R_{9-11}(p,b,c) \land R_{9-11}(p,c,b)) \land (\neg R_{9-11}(p,a,c) \land R_{9-11}(p,c,a)) \land R_{9-11}(p,a,a) \land R_{9-11}(p,b,b) \land R_{9-11}(p,c,c) \land (R_{9-11}(q,a,b) \land \neg R_{9-11}(q,b,a)) \land (\neg R_{9-11}(q,b,c) \land R_{9-11}(q,c,b)) \land (R_{9-11}(q,a,c) \land R_{9-11}(q,c,a)) \land R_{9-11}(q,a,a) \land R_{9-11}(q,b,b) \land R_{9-11}(q,c,c)$ prem.
119 | $(\neg R_{9-12}(p,a,b) \land R_{9-12}(p,b,a)) \land (R_{9-12}(p,b,c) \land R_{9-12}(p,c,b)) \land (\neg R_{9-12}(p,a,c) \land R_{9-12}(p,c,a)) \land R_{9-12}(p,a,a) \land R_{9-12}(p,b,b) \land R_{9-12}(p,c,c) \land (\neg R_{9-12}(q,a,b) \land R_{9-12}(q,b,a)) \land (R_{9-12}(q,b,c) \land \neg R_{9-12}(q,c,b)) \land (R_{9-12}(q,a,c) \land R_{9-12}(q,c,a)) \land R_{9-12}(q,a,a) \land R_{9-12}(q,b,b) \land R_{9-12}(q,c,c)$ prem.
120 | $(\neg R_{9-13}(p,a,b) \land R_{9-13}(p,b,a)) \land (R_{9-13}(p,b,c) \land R_{9-13}(p,c,b)) \land (\neg R_{9-13}(p,a,c) \land R_{9-13}(p,c,a)) \land R_{9-13}(p,a,a) \land R_{9-13}(p,b,b) \land R_{9-13}(p,c,c) \land (R_{9-13}(q,a,b) \land R_{9-13}(q,b,a)) \land (R_{9-13}(q,b,c) \land R_{9-13}(q,c,b)) \land (R_{9-13}(q,a,c) \land R_{9-13}(q,c,a)) \land R_{9-13}(q,a,a) \land R_{9-13}(q,b,b) \land R_{9-13}(q,c,c)$ prem.
121 | $(R_{10-1}(p,a,b) \land \neg R_{10-1}(p,b,a)) \land (R_{10-1}(p,b,c) \land R_{10-1}(p,c,b)) \land (R_{10-1}(p,a,c) \land \neg R_{10-1}(p,c,a)) \land R_{10-1}(p,a,a) \land R_{10-1}(p,b,b) \land R_{10-1}(p,c,c) \land (R_{10-1}(q,a,b) \land \neg R_{10-1}(q,b,a)) \land (R_{10-1}(q,b,c) \land \neg R_{10-1}(q,c,b)) \land (R_{10-1}(q,a,c) \land \neg R_{10-1}(q,c,a)) \land R_{10-1}(q,a,a) \land R_{10-1}(q,b,b) \land R_{10-1}(q,c,c)$ prem.
122 | $(R_{10-2}(p,a,b) \land \neg R_{10-2}(p,b,a)) \land (R_{10-2}(p,b,c) \land R_{10-2}(p,c,b)) \land (R_{10-2}(p,a,c) \land \neg R_{10-2}(p,c,a)) \land R_{10-2}(p,a,a) \land R_{10-2}(p,b,b) \land R_{10-2}(p,c,c) \land (R_{10-2}(q,a,b) \land \neg R_{10-2}(q,b,a)) \land (\neg R_{10-2}(q,b,c) \land R_{10-2}(q,c,b)) \land (R_{10-2}(q,a,c) \land \neg R_{10-2}(q,c,a)) \land R_{10-2}(q,a,a) \land R_{10-2}(q,b,b) \land R_{10-2}(q,c,c)$ prem.
123 | $(R_{10-3}(p,a,b) \land \neg R_{10-3}(p,b,a)) \land (R_{10-3}(p,b,c) \land R_{10-3}(p,c,b)) \land (R_{10-3}(p,a,c) \land \neg R_{10-3}(p,c,a)) \land R_{10-3}(p,a,a) \land R_{10-3}(p,b,b) \land R_{10-3}(p,c,c) \land (\neg R_{10-3}(q,a,b) \land R_{10-3}(q,b,a)) \land (R_{10-3}(q,b,c) \land \neg R_{10-3}(q,c,b)) \land (R_{10-3}(q,a,c) \land \neg R_{10-3}(q,c,a)) \land R_{10-3}(q,a,a) \land R_{10-3}(q,b,b) \land R_{10-3}(q,c,c)$ prem.
124 | $(R_{10-4}(p,a,b) \land \neg R_{10-4}(p,b,a)) \land (R_{10-4}(p,b,c) \land R_{10-4}(p,c,b)) \land (R_{10-4}(p,a,c) \land \neg R_{10-4}(p,c,a)) \land R_{10-4}(p,a,a) \land R_{10-4}(p,b,b) \land R_{10-4}(p,c,c) \land (\neg R_{10-4}(q,a,b) \land R_{10-4}(q,b,a)) \land (R_{10-4}(q,b,c) \land \neg R_{10-4}(q,c,b)) \land (\neg R_{10-4}(q,a,c) \land R_{10-4}(q,c,a)) \land R_{10-4}(q,a,a) \land R_{10-4}(q,b,b) \land R_{10-4}(q,c,c)$ prem.
125 | $(R_{10-5}(p,a,b) \land \neg R_{10-5}(p,b,a)) \land (R_{10-5}(p,b,c) \land R_{10-5}(p,c,b)) \land (R_{10-5}(p,a,c) \land \neg R_{10-5}(p,c,a)) \land R_{10-5}(p,a,a) \land R_{10-5}(p,b,b) \land R_{10-5}(p,c,c) \land (R_{10-5}(q,a,b) \land \neg R_{10-5}(q,b,a)) \land (\neg R_{10-5}(q,b,c) \land R_{10-5}(q,c,b)) \land (\neg R_{10-5}(q,a,c) \land R_{10-5}(q,c,a)) \land R_{10-5}(q,a,a) \land R_{10-5}(q,b,b) \land R_{10-5}(q,c,c)$ prem.
126 | $(R_{10-6}(p,a,b) \land \neg R_{10-6}(p,b,a)) \land (R_{10-6}(p,b,c) \land R_{10-6}(p,c,b)) \land (R_{10-6}(p,a,c) \land \neg R_{10-6}(p,c,a)) \land R_{10-6}(p,a,a) \land R_{10-6}(p,b,b) \land R_{10-6}(p,c,c) \land (\neg R_{10-6}(q,a,b) \land R_{10-6}(q,b,a)) \land (\neg R_{10-6}(q,b,c) \land R_{10-6}(q,c,b)) \land (\neg R_{10-6}(q,a,c) \land R_{10-6}(q,c,a)) \land R_{10-6}(q,a,a) \land R_{10-6}(q,b,b) \land R_{10-6}(q,c,c)$ prem.
127 | $(R_{10-7}(p,a,b) \land \neg R_{10-7}(p,b,a)) \land (R_{10-7}(p,b,c) \land R_{10-7}(p,c,b)) \land (R_{10-7}(p,a,c) \land \neg R_{10-7}(p,c,a)) \land R_{10-7}(p,a,a) \land R_{10-7}(p,b,b) \land R_{10-7}(p,c,c) \land (R_{10-7}(q,a,b) \land R_{10-7}(q,b,a)) \land (R_{10-7}(q,b,c) \land \neg R_{10-7}(q,c,b)) \land (R_{10-7}(q,a,c) \land \neg R_{10-7}(q,c,a)) \land R_{10-7}(q,a,a) \land R_{10-7}(q,b,b) \land R_{10-7}(q,c,c)$ prem.
128 | $(R_{10-8}(p,a,b) \land \neg R_{10-8}(p,b,a)) \land (R_{10-8}(p,b,c) \land R_{10-8}(p,c,b)) \land (R_{10-8}(p,a,c) \land \neg R_{10-8}(p,c,a)) \land R_{10-8}(p,a,a) \land R_{10-8}(p,b,b) \land R_{10-8}(p,c,c) \land (R_{10-8}(q,a,b) \land R_{10-8}(q,b,a)) \land (\neg R_{10-8}(q,b,c) \land R_{10-8}(q,c,b)) \land (\neg R_{10-8}(q,a,c) \land R_{10-8}(q,c,a)) \land R_{10-8}(q,a,a) \land R_{10-8}(q,b,b) \land R_{10-8}(q,c,c)$ prem.
129 | $(R_{10-9}(p,a,b) \land \neg R_{10-9}(p,b,a)) \land (R_{10-9}(p,b,c) \land R_{10-9}(p,c,b)) \land (R_{10-9}(p,a,c) \land \neg R_{10-9}(p,c,a)) \land R_{10-9}(p,a,a) \land R_{10-9}(p,b,b) \land R_{10-9}(p,c,c) \land (\neg R_{10-9}(q,a,b) \land R_{10-9}(q,b,a)) \land (R_{10-9}(q,b,c) \land R_{10-9}(q,c,b)) \land (\neg R_{10-9}(q,a,c) \land R_{10-9}(q,c,a)) \land R_{10-9}(q,a,a) \land R_{10-9}(q,b,b) \land R_{10-9}(q,c,c)$ prem.
130 | $(R_{10-10}(p,a,b) \land \neg R_{10-10}(p,b,a)) \land (R_{10-10}(p,b,c) \land R_{10-10}(p,c,b)) \land (R_{10-10}(p,a,c) \land \neg R_{10-10}(p,c,a)) \land R_{10-10}(p,a,a) \land R_{10-10}(p,b,b) \land R_{10-10}(p,c,c) \land (R_{10-10}(q,a,b) \land \neg R_{10-10}(q,b,a)) \land (R_{10-10}(q,b,c) \land R_{10-10}(q,c,b)) \land (R_{10-10}(q,a,c) \land \neg R_{10-10}(q,c,a)) \land R_{10-10}(q,a,a) \land R_{10-10}(q,b,b) \land R_{10-10}(q,c,c)$ prem.
131 | $(R_{10-11}(p,a,b) \land \neg R_{10-11}(p,b,a)) \land (R_{10-11}(p,b,c) \land R_{10-11}(p,c,b)) \land (R_{10-11}(p,a,c) \land \neg R_{10-11}(p,c,a)) \land R_{10-11}(p,a,a) \land R_{10-11}(p,b,b) \land R_{10-11}(p,c,c) \land (R_{10-11}(q,a,b) \land \neg R_{10-11}(q,b,a)) \land (\neg R_{10-11}(q,b,c) \land R_{10-11}(q,c,b)) \land (R_{10-11}(q,a,c) \land R_{10-11}(q,c,a)) \land R_{10-11}(q,a,a) \land R_{10-11}(q,b,b) \land R_{10-11}(q,c,c)$ prem.
132 | $(R_{10-12}(p,a,b) \land \neg R_{10-12}(p,b,a)) \land (R_{10-12}(p,b,c) \land R_{10-12}(p,c,b)) \land (R_{10-12}(p,a,c) \land \neg R_{10-12}(p,c,a)) \land R_{10-12}(p,a,a) \land R_{10-12}(p,b,b) \land R_{10-12}(p,c,c) \land (\neg R_{10-12}(q,a,b) \land R_{10-12}(q,b,a)) \land (R_{10-12}(q,b,c) \land \neg R_{10-12}(q,c,b)) \land (R_{10-12}(q,a,c) \land R_{10-12}(q,c,a)) \land R_{10-12}(q,a,a) \land R_{10-12}(q,b,b) \land R_{10-12}(q,c,c)$ prem.
133 | $(R_{10-13}(p,a,b) \land \neg R_{10-13}(p,b,a)) \land (R_{10-13}(p,b,c) \land R_{10-13}(p,c,b)) \land (R_{10-13}(p,a,c) \land \neg R_{10-13}(p,c,a)) \land R_{10-13}(p,a,a) \land R_{10-13}(p,b,b) \land R_{10-13}(p,c,c) \land (R_{10-13}(q,a,b) \land R_{10-13}(q,b,a)) \land (R_{10-13}(q,b,c) \land R_{10-13}(q,c,b)) \land (R_{10-13}(q,a,c) \land R_{10-13}(q,c,a)) \land R_{10-13}(q,a,a) \land R_{10-13}(q,b,b) \land R_{10-13}(q,c,c)$ prem.
134 | $(R_{11-1}(p,a,b) \land \neg R_{11-1}(p,b,a)) \land (\neg R_{11-1}(p,b,c) \land R_{11-1}(p,c,b)) \land (R_{11-1}(p,a,c) \land R_{11-1}(p,c,a)) \land R_{11-1}(p,a,a) \land R_{11-1}(p,b,b) \land R_{11-1}(p,c,c) \land (R_{11-1}(q,a,b) \land \neg R_{11-1}(q,b,a)) \land (R_{11-1}(q,b,c) \land \neg R_{11-1}(q,c,b)) \land (R_{11-1}(q,a,c) \land \neg R_{11-1}(q,c,a)) \land R_{11-1}(q,a,a) \land R_{11-1}(q,b,b) \land R_{11-1}(q,c,c)$ prem.
135 | $(R_{11-2}(p,a,b) \land \neg R_{11-2}(p,b,a)) \land (\neg R_{11-2}(p,b,c) \land R_{11-2}(p,c,b)) \land (R_{11-2}(p,a,c) \land R_{11-2}(p,c,a)) \land R_{11-2}(p,a,a) \land R_{11-2}(p,b,b) \land R_{11-2}(p,c,c) \land (R_{11-2}(q,a,b) \land \neg R_{11-2}(q,b,a)) \land (\neg R_{11-2}(q,b,c) \land R_{11-2}(q,c,b)) \land (R_{11-2}(q,a,c) \land \neg R_{11-2}(q,c,a)) \land R_{11-2}(q,a,a) \land R_{11-2}(q,b,b) \land R_{11-2}(q,c,c)$ prem.
136 | $(R_{11-3}(p,a,b) \land \neg R_{11-3}(p,b,a)) \land (\neg R_{11-3}(p,b,c) \land R_{11-3}(p,c,b)) \land (R_{11-3}(p,a,c) \land R_{11-3}(p,c,a)) \land R_{11-3}(p,a,a) \land R_{11-3}(p,b,b) \land R_{11-3}(p,c,c) \land (\neg R_{11-3}(q,a,b) \land R_{11-3}(q,b,a)) \land (R_{11-3}(q,b,c) \land \neg R_{11-3}(q,c,b)) \land (R_{11-3}(q,a,c) \land \neg R_{11-3}(q,c,a)) \land R_{11-3}(q,a,a) \land R_{11-3}(q,b,b) \land R_{11-3}(q,c,c)$ prem.
137 | $(R_{11-4}(p,a,b) \land \neg R_{11-4}(p,b,a)) \land (\neg R_{11-4}(p,b,c) \land R_{11-4}(p,c,b)) \land (R_{11-4}(p,a,c) \land R_{11-4}(p,c,a)) \land R_{11-4}(p,a,a) \land R_{11-4}(p,b,b) \land R_{11-4}(p,c,c) \land (\neg R_{11-4}(q,a,b) \land R_{11-4}(q,b,a)) \land (R_{11-4}(q,b,c) \land \neg R_{11-4}(q,c,b)) \land (\neg R_{11-4}(q,a,c) \land R_{11-4}(q,c,a)) \land R_{11-4}(q,a,a) \land R_{11-4}(q,b,b) \land R_{11-4}(q,c,c)$ prem.
138 | $(R_{11-5}(p,a,b) \land \neg R_{11-5}(p,b,a)) \land (\neg R_{11-5}(p,b,c) \land R_{11-5}(p,c,b)) \land (R_{11-5}(p,a,c) \land R_{11-5}(p,c,a)) \land R_{11-5}(p,a,a) \land R_{11-5}(p,b,b) \land R_{11-5}(p,c,c) \land (R_{11-5}(q,a,b) \land \neg R_{11-5}(q,b,a)) \land (\neg R_{11-5}(q,b,c) \land R_{11-5}(q,c,b)) \land (\neg R_{11-5}(q,a,c) \land R_{11-5}(q,c,a)) \land R_{11-5}(q,a,a) \land R_{11-5}(q,b,b) \land R_{11-5}(q,c,c)$ prem.
139 | $(R_{11-6}(p,a,b) \land \neg R_{11-6}(p,b,a)) \land (\neg R_{11-6}(p,b,c) \land R_{11-6}(p,c,b)) \land (R_{11-6}(p,a,c) \land R_{11-6}(p,c,a)) \land R_{11-6}(p,a,a) \land R_{11-6}(p,b,b) \land R_{11-6}(p,c,c) \land (\neg R_{11-6}(q,a,b) \land R_{11-6}(q,b,a)) \land (\neg R_{11-6}(q,b,c) \land R_{11-6}(q,c,b)) \land (\neg R_{11-6}(q,a,c) \land R_{11-6}(q,c,a)) \land R_{11-6}(q,a,a) \land R_{11-6}(q,b,b) \land R_{11-6}(q,c,c)$ prem.
140 | $(R_{11-7}(p,a,b) \land \neg R_{11-7}(p,b,a)) \land (\neg R_{11-7}(p,b,c) \land R_{11-7}(p,c,b)) \land (R_{11-7}(p,a,c) \land R_{11-7}(p,c,a)) \land R_{11-7}(p,a,a) \land R_{11-7}(p,b,b) \land R_{11-7}(p,c,c) \land (R_{11-7}(q,a,b) \land R_{11-7}(q,b,a)) \land (R_{11-7}(q,b,c) \land \neg R_{11-7}(q,c,b)) \land (R_{11-7}(q,a,c) \land \neg R_{11-7}(q,c,a)) \land R_{11-7}(q,a,a) \land R_{11-7}(q,b,b) \land R_{11-7}(q,c,c)$ prem.
141 | $(R_{11-8}(p,a,b) \land \neg R_{11-8}(p,b,a)) \land (\neg R_{11-8}(p,b,c) \land R_{11-8}(p,c,b)) \land (R_{11-8}(p,a,c) \land R_{11-8}(p,c,a)) \land R_{11-8}(p,a,a) \land R_{11-8}(p,b,b) \land R_{11-8}(p,c,c) \land (R_{11-8}(q,a,b) \land R_{11-8}(q,b,a)) \land (\neg R_{11-8}(q,b,c) \land R_{11-8}(q,c,b)) \land (\neg R_{11-8}(q,a,c) \land R_{11-8}(q,c,a)) \land R_{11-8}(q,a,a) \land R_{11-8}(q,b,b) \land R_{11-8}(q,c,c)$ prem.

142 | $(R_{11\text{-}9}(p,a,b) \land \neg R_{11\text{-}9}(p,b,a)) \land (\neg R_{11\text{-}9}(p,b,c) \land R_{11\text{-}9}(p,c,b)) \land (R_{11\text{-}9}(p,a,c) \land R_{11\text{-}9}(p,c,a)) \land R_{11\text{-}9}(p,a,a) \land R_{11\text{-}9}(p,b,b) \land R_{11\text{-}9}(p,c,c) \land (\neg R_{11\text{-}9}(q,a,b) \land R_{11\text{-}9}(q,b,a)) \land (R_{11\text{-}9}(q,b,c) \land R_{11\text{-}9}(q,c,b)) \land (\neg R_{11\text{-}9}(q,a,c) \land R_{11\text{-}9}(q,c,a)) \land R_{11\text{-}9}(q,a,a) \land R_{11\text{-}9}(q,b,b) \land R_{11\text{-}9}(q,c,c)$ prem.
143 | $(R_{11\text{-}10}(p,a,b) \land \neg R_{11\text{-}10}(p,b,a)) \land (\neg R_{11\text{-}10}(p,b,c) \land R_{11\text{-}10}(p,c,b)) \land (R_{11\text{-}10}(p,a,c) \land R_{11\text{-}10}(p,c,a)) \land R_{11\text{-}10}(p,a,a) \land R_{11\text{-}10}(p,b,b) \land R_{11\text{-}10}(p,c,c) \land (R_{11\text{-}10}(q,a,b) \land \neg R_{11\text{-}10}(q,b,a)) \land (R_{11\text{-}10}(q,b,c) \land R_{11\text{-}10}(q,c,b)) \land (R_{11\text{-}10}(q,a,c) \land \neg R_{11\text{-}10}(q,c,a)) \land R_{11\text{-}10}(q,a,a) \land R_{11\text{-}10}(q,b,b) \land R_{11\text{-}10}(q,c,c)$ prem.
144 | $(R_{11\text{-}11}(p,a,b) \land \neg R_{11\text{-}11}(p,b,a)) \land (\neg R_{11\text{-}11}(p,b,c) \land R_{11\text{-}11}(p,c,b)) \land (R_{11\text{-}11}(p,a,c) \land R_{11\text{-}11}(p,c,a)) \land R_{11\text{-}11}(p,a,a) \land R_{11\text{-}11}(p,b,b) \land R_{11\text{-}11}(p,c,c) \land (R_{11\text{-}11}(q,a,b) \land \neg R_{11\text{-}11}(q,b,a)) \land (\neg R_{11\text{-}11}(q,b,c) \land R_{11\text{-}11}(q,c,b)) \land (R_{11\text{-}11}(q,a,c) \land R_{11\text{-}11}(q,c,a)) \land R_{11\text{-}11}(q,a,a) \land R_{11\text{-}11}(q,b,b) \land R_{11\text{-}11}(q,c,c)$ prem.
145 | $(R_{11\text{-}12}(p,a,b) \land \neg R_{11\text{-}12}(p,b,a)) \land (\neg R_{11\text{-}12}(p,b,c) \land R_{11\text{-}12}(p,c,b)) \land (R_{11\text{-}12}(p,a,c) \land R_{11\text{-}12}(p,c,a)) \land R_{11\text{-}12}(p,a,a) \land R_{11\text{-}12}(p,b,b) \land R_{11\text{-}12}(p,c,c) \land (\neg R_{11\text{-}12}(q,a,b) \land R_{11\text{-}12}(q,b,a)) \land (R_{11\text{-}12}(q,b,c) \land \neg R_{11\text{-}12}(q,c,b)) \land (R_{11\text{-}12}(q,a,c) \land R_{11\text{-}12}(q,c,a)) \land R_{11\text{-}12}(q,a,a) \land R_{11\text{-}12}(q,b,b) \land R_{11\text{-}12}(q,c,c)$ prem.
146 | $(R_{11\text{-}13}(p,a,b) \land \neg R_{11\text{-}13}(p,b,a)) \land (\neg R_{11\text{-}13}(p,b,c) \land R_{11\text{-}13}(p,c,b)) \land (R_{11\text{-}13}(p,a,c) \land R_{11\text{-}13}(p,c,a)) \land R_{11\text{-}13}(p,a,a) \land R_{11\text{-}13}(p,b,b) \land R_{11\text{-}13}(p,c,c) \land (R_{11\text{-}13}(q,a,b) \land R_{11\text{-}13}(q,b,a)) \land (R_{11\text{-}13}(q,b,c) \land R_{11\text{-}13}(q,c,b)) \land (R_{11\text{-}13}(q,a,c) \land R_{11\text{-}13}(q,c,a)) \land R_{11\text{-}13}(q,a,a) \land R_{11\text{-}13}(q,b,b) \land R_{11\text{-}13}(q,c,c)$ prem.
147 | $(\neg R_{12\text{-}1}(p,a,b) \land R_{12\text{-}1}(p,b,a)) \land (R_{12\text{-}1}(p,b,c) \land \neg R_{12\text{-}1}(p,c,b)) \land (R_{12\text{-}1}(p,a,c) \land R_{12\text{-}1}(p,c,a)) \land R_{12\text{-}1}(p,a,a) \land R_{12\text{-}1}(p,b,b) \land R_{12\text{-}1}(p,c,c) \land (R_{12\text{-}1}(q,a,b) \land \neg R_{12\text{-}1}(q,b,a)) \land (R_{12\text{-}1}(q,b,c) \land \neg R_{12\text{-}1}(q,c,b)) \land (R_{12\text{-}1}(q,a,c) \land \neg R_{12\text{-}1}(q,c,a)) \land R_{12\text{-}1}(q,a,a) \land R_{12\text{-}1}(q,b,b) \land R_{12\text{-}1}(q,c,c)$ prem.
148 | $(\neg R_{12\text{-}2}(p,a,b) \land R_{12\text{-}2}(p,b,a)) \land (R_{12\text{-}2}(p,b,c) \land \neg R_{12\text{-}2}(p,c,b)) \land (R_{12\text{-}2}(p,a,c) \land R_{12\text{-}2}(p,c,a)) \land R_{12\text{-}2}(p,a,a) \land R_{12\text{-}2}(p,b,b) \land R_{12\text{-}2}(p,c,c) \land (R_{12\text{-}2}(q,a,b) \land \neg R_{12\text{-}2}(q,b,a)) \land (\neg R_{12\text{-}2}(q,b,c) \land R_{12\text{-}2}(q,c,b)) \land (R_{12\text{-}2}(q,a,c) \land \neg R_{12\text{-}2}(q,c,a)) \land R_{12\text{-}2}(q,a,a) \land R_{12\text{-}2}(q,b,b) \land R_{12\text{-}2}(q,c,c)$ prem.
149 | $(\neg R_{12\text{-}3}(p,a,b) \land R_{12\text{-}3}(p,b,a)) \land (R_{12\text{-}3}(p,b,c) \land \neg R_{12\text{-}3}(p,c,b)) \land (R_{12\text{-}3}(p,a,c) \land R_{12\text{-}3}(p,c,a)) \land R_{12\text{-}3}(p,a,a) \land R_{12\text{-}3}(p,b,b) \land R_{12\text{-}3}(p,c,c) \land (\neg R_{12\text{-}3}(q,a,b) \land R_{12\text{-}3}(q,b,a)) \land (R_{12\text{-}3}(q,b,c) \land \neg R_{12\text{-}3}(q,c,b)) \land (R_{12\text{-}3}(q,a,c) \land \neg R_{12\text{-}3}(q,c,a)) \land R_{12\text{-}3}(q,a,a) \land R_{12\text{-}3}(q,b,b) \land R_{12\text{-}3}(q,c,c)$ prem.
150 | $(\neg R_{12\text{-}4}(p,a,b) \land R_{12\text{-}4}(p,b,a)) \land (R_{12\text{-}4}(p,b,c) \land \neg R_{12\text{-}4}(p,c,b)) \land (R_{12\text{-}4}(p,a,c) \land R_{12\text{-}4}(p,c,a)) \land R_{12\text{-}4}(p,a,a) \land R_{12\text{-}4}(p,b,b) \land R_{12\text{-}4}(p,c,c) \land (\neg R_{12\text{-}4}(q,a,b) \land R_{12\text{-}4}(q,b,a)) \land (R_{12\text{-}4}(q,b,c) \land \neg R_{12\text{-}4}(q,c,b)) \land (\neg R_{12\text{-}4}(q,a,c) \land R_{12\text{-}4}(q,c,a)) \land R_{12\text{-}4}(q,a,a) \land R_{12\text{-}4}(q,b,b) \land R_{12\text{-}4}(q,c,c)$ prem.
151 | $(\neg R_{12\text{-}5}(p,a,b) \land R_{12\text{-}5}(p,b,a)) \land (R_{12\text{-}5}(p,b,c) \land \neg R_{12\text{-}5}(p,c,b)) \land (R_{12\text{-}5}(p,a,c) \land R_{12\text{-}5}(p,c,a)) \land R_{12\text{-}5}(p,a,a) \land R_{12\text{-}5}(p,b,b) \land R_{12\text{-}5}(p,c,c) \land (R_{12\text{-}5}(q,a,b) \land \neg R_{12\text{-}5}(q,b,a)) \land (\neg R_{12\text{-}5}(q,b,c) \land R_{12\text{-}5}(q,c,b)) \land (\neg R_{12\text{-}5}(q,a,c) \land R_{12\text{-}5}(q,c,a)) \land R_{12\text{-}5}(q,a,a) \land R_{12\text{-}5}(q,b,b) \land R_{12\text{-}5}(q,c,c)$ prem.
152 | $(\neg R_{12\text{-}6}(p,a,b) \land R_{12\text{-}6}(p,b,a)) \land (R_{12\text{-}6}(p,b,c) \land \neg R_{12\text{-}6}(p,c,b)) \land (R_{12\text{-}6}(p,a,c) \land R_{12\text{-}6}(p,c,a)) \land R_{12\text{-}6}(p,a,a) \land R_{12\text{-}6}(p,b,b) \land R_{12\text{-}6}(p,c,c) \land (\neg R_{12\text{-}6}(q,a,b) \land R_{12\text{-}6}(q,b,a)) \land (\neg R_{12\text{-}6}(q,b,c) \land R_{12\text{-}6}(q,c,b)) \land (\neg R_{12\text{-}6}(q,a,c) \land R_{12\text{-}6}(q,c,a)) \land R_{12\text{-}6}(q,a,a) \land R_{12\text{-}6}(q,b,b) \land R_{12\text{-}6}(q,c,c)$ prem.
153 | $(\neg R_{12\text{-}7}(p,a,b) \land R_{12\text{-}7}(p,b,a)) \land (R_{12\text{-}7}(p,b,c) \land \neg R_{12\text{-}7}(p,c,b)) \land (R_{12\text{-}7}(p,a,c) \land R_{12\text{-}7}(p,c,a)) \land R_{12\text{-}7}(p,a,a) \land R_{12\text{-}7}(p,b,b) \land R_{12\text{-}7}(p,c,c) \land (R_{12\text{-}7}(q,a,b) \land R_{12\text{-}7}(q,b,a)) \land (R_{12\text{-}7}(q,b,c) \land \neg R_{12\text{-}7}(q,c,b)) \land (R_{12\text{-}7}(q,a,c) \land \neg R_{12\text{-}7}(q,c,a)) \land R_{12\text{-}7}(q,a,a) \land R_{12\text{-}7}(q,b,b) \land R_{12\text{-}7}(q,c,c)$ prem.
154 | $(\neg R_{12\text{-}8}(p,a,b) \land R_{12\text{-}8}(p,b,a)) \land (R_{12\text{-}8}(p,b,c) \land \neg R_{12\text{-}8}(p,c,b)) \land (R_{12\text{-}8}(p,a,c) \land R_{12\text{-}8}(p,c,a)) \land R_{12\text{-}8}(p,a,a) \land R_{12\text{-}8}(p,b,b) \land R_{12\text{-}8}(p,c,c) \land (R_{12\text{-}8}(q,a,b) \land R_{12\text{-}8}(q,b,a)) \land (\neg R_{12\text{-}8}(q,b,c) \land R_{12\text{-}8}(q,c,b)) \land (\neg R_{12\text{-}8}(q,a,c) \land R_{12\text{-}8}(q,c,a)) \land R_{12\text{-}8}(q,a,a) \land R_{12\text{-}8}(q,b,b) \land R_{12\text{-}8}(q,c,c)$ prem.
155 | $(\neg R_{12\text{-}9}(p,a,b) \land R_{12\text{-}9}(p,b,a)) \land (R_{12\text{-}9}(p,b,c) \land \neg R_{12\text{-}9}(p,c,b)) \land (R_{12\text{-}9}(p,a,c) \land R_{12\text{-}9}(p,c,a)) \land R_{12\text{-}9}(p,a,a) \land R_{12\text{-}9}(p,b,b) \land R_{12\text{-}9}(p,c,c) \land (\neg R_{12\text{-}9}(q,a,b) \land R_{12\text{-}9}(q,b,a)) \land (R_{12\text{-}9}(q,b,c) \land R_{12\text{-}9}(q,c,b)) \land (\neg R_{12\text{-}9}(q,a,c) \land R_{12\text{-}9}(q,c,a)) \land R_{12\text{-}9}(q,a,a) \land R_{12\text{-}9}(q,b,b) \land R_{12\text{-}9}(q,c,c)$ prem.
156 | $(\neg R_{12\text{-}10}(p,a,b) \land R_{12\text{-}10}(p,b,a)) \land (R_{12\text{-}10}(p,b,c) \land \neg R_{12\text{-}10}(p,c,b)) \land (R_{12\text{-}10}(p,a,c) \land R_{12\text{-}10}(p,c,a)) \land R_{12\text{-}10}(p,a,a) \land R_{12\text{-}10}(p,b,b) \land R_{12\text{-}10}(p,c,c) \land (R_{12\text{-}10}(q,a,b) \land \neg R_{12\text{-}10}(q,b,a)) \land (R_{12\text{-}10}(q,b,c) \land R_{12\text{-}10}(q,c,b)) \land (R_{12\text{-}10}(q,a,c) \land \neg R_{12\text{-}10}(q,c,a)) \land R_{12\text{-}10}(q,a,a) \land R_{12\text{-}10}(q,b,b) \land R_{12\text{-}10}(q,c,c)$ prem.
157 | $(\neg R_{12\text{-}11}(p,a,b) \land R_{12\text{-}11}(p,b,a)) \land (R_{12\text{-}11}(p,b,c) \land \neg R_{12\text{-}11}(p,c,b)) \land (R_{12\text{-}11}(p,a,c) \land R_{12\text{-}11}(p,c,a)) \land R_{12\text{-}11}(p,a,a) \land R_{12\text{-}11}(p,b,b) \land R_{12\text{-}11}(p,c,c) \land (R_{12\text{-}11}(q,a,b) \land \neg R_{12\text{-}11}(q,b,a)) \land (\neg R_{12\text{-}11}(q,b,c) \land R_{12\text{-}11}(q,c,b)) \land (R_{12\text{-}11}(q,a,c) \land R_{12\text{-}11}(q,c,a)) \land R_{12\text{-}11}(q,a,a) \land R_{12\text{-}11}(q,b,b) \land R_{12\text{-}11}(q,c,c)$ prem.
158 | $(\neg R_{12\text{-}12}(p,a,b) \land R_{12\text{-}12}(p,b,a)) \land (R_{12\text{-}12}(p,b,c) \land \neg R_{12\text{-}12}(p,c,b)) \land (R_{12\text{-}12}(p,a,c) \land R_{12\text{-}12}(p,c,a)) \land R_{12\text{-}12}(p,a,a) \land R_{12\text{-}12}(p,b,b) \land R_{12\text{-}12}(p,c,c) \land (\neg R_{12\text{-}12}(q,a,b) \land R_{12\text{-}12}(q,b,a)) \land (R_{12\text{-}12}(q,b,c) \land \neg R_{12\text{-}12}(q,c,b)) \land (R_{12\text{-}12}(q,a,c) \land R_{12\text{-}12}(q,c,a)) \land R_{12\text{-}12}(q,a,a) \land R_{12\text{-}12}(q,b,b) \land R_{12\text{-}12}(q,c,c)$ prem.
159 | $(\neg R_{12\text{-}13}(p,a,b) \land R_{12\text{-}13}(p,b,a)) \land (R_{12\text{-}13}(p,b,c) \land \neg R_{12\text{-}13}(p,c,b)) \land (R_{12\text{-}13}(p,a,c) \land R_{12\text{-}13}(p,c,a)) \land R_{12\text{-}13}(p,a,a) \land R_{12\text{-}13}(p,b,b) \land R_{12\text{-}13}(p,c,c) \land (R_{12\text{-}13}(q,a,b) \land R_{12\text{-}13}(q,b,a)) \land (R_{12\text{-}13}(q,b,c) \land R_{12\text{-}13}(q,c,b)) \land (R_{12\text{-}13}(q,a,c) \land R_{12\text{-}13}(q,c,a)) \land R_{12\text{-}13}(q,a,a) \land R_{12\text{-}13}(q,b,b) \land R_{12\text{-}13}(q,c,c)$ prem.
160 | $(R_{13\text{-}1}(p,a,b) \land R_{13\text{-}1}(p,b,a)) \land (R_{13\text{-}1}(p,b,c) \land R_{13\text{-}1}(p,c,b)) \land (R_{13\text{-}1}(p,a,c) \land R_{13\text{-}1}(p,c,a)) \land R_{13\text{-}1}(p,a,a) \land R_{13\text{-}1}(p,b,b) \land R_{13\text{-}1}(p,c,c) \land (R_{13\text{-}1}(q,a,b) \land \neg R_{13\text{-}1}(q,b,a)) \land (R_{13\text{-}1}(q,b,c) \land \neg R_{13\text{-}1}(q,c,b)) \land (R_{13\text{-}1}(q,a,c) \land \neg R_{13\text{-}1}(q,c,a)) \land R_{13\text{-}1}(q,a,a) \land R_{13\text{-}1}(q,b,b) \land R13\text{-}1(q,c,c)$ prem.
161 | $(R_{13\text{-}2}(p,a,b) \land R_{13\text{-}2}(p,b,a)) \land (R_{13\text{-}2}(p,b,c) \land R_{13\text{-}2}(p,c,b)) \land (R_{13\text{-}2}(p,a,c) \land R_{13\text{-}2}(p,c,a)) \land R_{13\text{-}2}(p,a,a) \land R_{13\text{-}2}(p,b,b) \land R_{13\text{-}2}(p,c,c) \land (R_{13\text{-}2}(q,a,b) \land \neg R_{13\text{-}2}(q,b,a)) \land (\neg R_{13\text{-}2}(q,b,c) \land R_{13\text{-}2}(q,c,b)) \land (R_{13\text{-}2}(q,a,c) \land \neg R_{13\text{-}2}(q,c,a)) \land R_{13\text{-}2}(q,a,a) \land R_{13\text{-}2}(q,b,b) \land R_{13\text{-}2}(q,c,c)$ prem.
162 | $(R_{13\text{-}3}(p,a,b) \land R_{13\text{-}3}(p,b,a)) \land (R_{13\text{-}3}(p,b,c) \land R_{13\text{-}3}(p,c,b)) \land (R_{13\text{-}3}(p,a,c) \land R_{13\text{-}3}(p,c,a)) \land R_{13\text{-}3}(p,a,a) \land R_{13\text{-}3}(p,b,b) \land R_{13\text{-}3}(p,c,c) \land (\neg R_{13\text{-}3}(q,a,b) \land R_{13\text{-}3}(q,b,a)) \land (R_{13\text{-}3}(q,b,c) \land \neg R_{13\text{-}3}(q,c,b)) \land (R_{13\text{-}3}(q,a,c) \land \neg R_{13\text{-}3}(q,c,a)) \land R_{13\text{-}3}(q,a,a) \land R_{13\text{-}3}(q,b,b) \land R_{13\text{-}3}(q,c,c)$ prem.
163 | $(R_{13\text{-}4}(p,a,b) \land R_{13\text{-}4}(p,b,a)) \land (R_{13\text{-}4}(p,b,c) \land R_{13\text{-}4}(p,c,b)) \land (R_{13\text{-}4}(p,a,c) \land R_{13\text{-}4}(p,c,a)) \land R_{13\text{-}4}(p,a,a) \land R_{13\text{-}4}(p,b,b) \land R_{13\text{-}4}(p,c,c) \land (\neg R_{13\text{-}4}(q,a,b) \land R_{13\text{-}4}(q,b,a)) \land (R_{13\text{-}4}(q,b,c) \land \neg R_{13\text{-}4}(q,c,b)) \land (\neg R_{13\text{-}4}(q,a,c) \land R_{13\text{-}4}(q,c,a)) \land R_{13\text{-}4}(q,a,a) \land R_{13\text{-}4}(q,b,b) \land R_{13\text{-}4}(q,c,c)$ prem.
164 | $(R_{13\text{-}5}(p,a,b) \land R_{13\text{-}5}(p,b,a)) \land (R_{13\text{-}5}(p,b,c) \land R_{13\text{-}5}(p,c,b)) \land (R_{13\text{-}5}(p,a,c) \land R_{13\text{-}5}(p,c,a)) \land R_{13\text{-}5}(p,a,a) \land R_{13\text{-}5}(p,b,b) \land R_{13\text{-}5}(p,c,c) \land (R_{13\text{-}5}(q,a,b) \land \neg R_{13\text{-}5}(q,b,a)) \land (\neg R_{13\text{-}5}(q,b,c) \land R_{13\text{-}5}(q,c,b)) \land (\neg R_{13\text{-}5}(q,a,c) \land R_{13\text{-}5}(q,c,a)) \land R_{13\text{-}5}(q,a,a) \land R_{13\text{-}5}(q,b,b) \land R_{13\text{-}5}(q,c,c)$ prem.
165 | $(R_{13\text{-}6}(p,a,b) \land R_{13\text{-}6}(p,b,a)) \land (R_{13\text{-}6}(p,b,c) \land R_{13\text{-}6}(p,c,b)) \land (R_{13\text{-}6}(p,a,c) \land R_{13\text{-}6}(p,c,a)) \land R_{13\text{-}6}(p,a,a) \land R_{13\text{-}6}(p,b,b) \land R_{13\text{-}6}(p,c,c) \land (\neg R_{13\text{-}6}(q,a,b) \land R_{13\text{-}6}(q,b,a)) \land (\neg R_{13\text{-}6}(q,b,c) \land R_{13\text{-}6}(q,c,b)) \land (\neg R_{13\text{-}6}(q,a,c) \land R_{13\text{-}6}(q,c,a)) \land R_{13\text{-}6}(q,a,a) \land R_{13\text{-}6}(q,b,b) \land R_{13\text{-}6}(q,c,c)$ prem.
166 | $(R_{13\text{-}7}(p,a,b) \land R_{13\text{-}7}(p,b,a)) \land (R_{13\text{-}7}(p,b,c) \land R_{13\text{-}7}(p,c,b)) \land (R_{13\text{-}7}(p,a,c) \land R_{13\text{-}7}(p,c,a)) \land R_{13\text{-}7}(p,a,a) \land R_{13\text{-}7}(p,b,b) \land R_{13\text{-}7}(p,c,c) \land (R_{13\text{-}7}(q,a,b) \land R_{13\text{-}7}(q,b,a)) \land (R_{13\text{-}7}(q,b,c) \land \neg R_{13\text{-}7}(q,c,b)) \land (R_{13\text{-}7}(q,a,c) \land \neg R_{13\text{-}7}(q,c,a)) \land R_{13\text{-}7}(q,a,a) \land R_{13\text{-}7}(q,b,b) \land R_{13\text{-}7}(q,c,c)$ prem.
167 | $(R_{13\text{-}8}(p,a,b) \land R_{13\text{-}8}(p,b,a)) \land (R_{13\text{-}8}(p,b,c) \land R_{13\text{-}8}(p,c,b)) \land (R_{13\text{-}8}(p,a,c) \land R_{13\text{-}8}(p,c,a)) \land R_{13\text{-}8}(p,a,a) \land R_{13\text{-}8}(p,b,b) \land R_{13\text{-}8}(p,c,c) \land (R_{13\text{-}8}(q,a,b) \land R_{13\text{-}8}(q,b,a)) \land (\neg R_{13\text{-}8}(q,b,c) \land R_{13\text{-}8}(q,c,b)) \land (\neg R_{13\text{-}8}(q,a,c) \land R_{13\text{-}8}(q,c,a)) \land R_{13\text{-}8}(q,a,a) \land R_{13\text{-}8}(q,b,b) \land R_{13\text{-}8}(q,c,c)$ prem.
168 | $(R_{13\text{-}9}(p,a,b) \land R_{13\text{-}9}(p,b,a)) \land (R_{13\text{-}9}(p,b,c) \land R_{13\text{-}9}(p,c,b)) \land (R_{13\text{-}9}(p,a,c) \land R_{13\text{-}9}(p,c,a)) \land R_{13\text{-}9}(p,a,a) \land R_{13\text{-}9}(p,b,b) \land R_{13\text{-}9}(p,c,c) \land (\neg R_{13\text{-}9}(q,a,b) \land R_{13\text{-}9}(q,b,a)) \land (R_{13\text{-}9}(q,b,c) \land R_{13\text{-}9}(q,c,b)) \land (\neg R_{13\text{-}9}(q,a,c) \land R_{13\text{-}9}(q,c,a)) \land R_{13\text{-}9}(q,a,a) \land R_{13\text{-}9}(q,b,b) \land R_{13\text{-}9}(q,c,c)$ prem.
169 | $(R_{13\text{-}10}(p,a,b) \land R_{13\text{-}10}(p,b,a)) \land (R_{13\text{-}10}(p,b,c) \land R_{13\text{-}10}(p,c,b)) \land (R_{13\text{-}10}(p,a,c) \land R_{13\text{-}10}(p,c,a)) \land R_{13\text{-}10}(p,a,a) \land R_{13\text{-}10}(p,b,b) \land R_{13\text{-}10}(p,c,c) \land (R_{13\text{-}10}(q,a,b) \land \neg R_{13\text{-}10}(q,b,a)) \land (R_{13\text{-}10}(q,b,c) \land R_{13\text{-}10}(q,c,b)) \land (R_{13\text{-}10}(q,a,c) \land \neg R_{13\text{-}10}(q,c,a)) \land R_{13\text{-}10}(q,a,a) \land R_{13\text{-}10}(q,b,b) \land R_{13\text{-}10}(q,c,c)$ prem.
170 | $(R_{13\text{-}11}(p,a,b) \land R_{13\text{-}11}(p,b,a)) \land (R_{13\text{-}11}(p,b,c) \land R_{13\text{-}11}(p,c,b)) \land (R_{13\text{-}11}(p,a,c) \land R_{13\text{-}11}(p,c,a)) \land R_{13\text{-}11}(p,a,a) \land R_{13\text{-}11}(p,b,b) \land R_{13\text{-}11}(p,c,c) \land (R_{13\text{-}11}(q,a,b) \land \neg R_{13\text{-}11}(q,b,a)) \land (\neg R_{13\text{-}11}(q,b,c) \land R_{13\text{-}11}(q,c,b)) \land (R_{13\text{-}11}(q,a,c) \land R_{13\text{-}11}(q,c,a)) \land R_{13\text{-}11}(q,a,a) \land R_{13\text{-}11}(q,b,b) \land R_{13\text{-}11}(q,c,c)$ prem.
171 | $(R_{13\text{-}12}(p,a,b) \land R_{13\text{-}12}(p,b,a)) \land (R_{13\text{-}12}(p,b,c) \land R_{13\text{-}12}(p,c,b)) \land (R_{13\text{-}12}(p,a,c) \land R_{13\text{-}12}(p,c,a)) \land R_{13\text{-}12}(p,a,a) \land R_{13\text{-}12}(p,b,b) \land R_{13\text{-}12}(p,c,c) \land (\neg R_{13\text{-}12}(q,a,b) \land R_{13\text{-}12}(q,b,a)) \land (R_{13\text{-}12}(q,b,c) \land \neg R_{13\text{-}12}(q,c,b)) \land (R_{13\text{-}12}(q,a,c) \land R_{13\text{-}12}(q,c,a)) \land R_{13\text{-}12}(q,a,a) \land R_{13\text{-}12}(q,b,b) \land R_{13\text{-}12}(q,c,c)$ prem.
172 | $(R_{13\text{-}13}(p,a,b) \land R_{13\text{-}13}(p,b,a)) \land (R_{13\text{-}13}(p,b,c) \land R_{13\text{-}13}(p,c,b)) \land (R_{13\text{-}13}(p,a,c) \land R_{13\text{-}13}(p,c,a)) \land R_{13\text{-}13}(p,a,a) \land R_{13\text{-}13}(p,b,b) \land R_{13\text{-}13}(p,c,c) \land (R_{13\text{-}13}(q,a,b) \land R_{13\text{-}13}(q,b,a)) \land (R_{13\text{-}13}(q,b,c) \land R_{13\text{-}13}(q,c,b)) \land (R_{13\text{-}13}(q,a,c) \land R_{13\text{-}13}(q,c,a)) \land R_{13\text{-}13}(q,a,a) \land R_{13\text{-}13}(q,b,b) \land R_{13\text{-}13}(q,c,c)$ prem.
173 | $\forall X(\boldsymbol{P}(X) \to \forall x \forall y((A(x) \land A(y)) \to ((X(s,x,y) \lor \neg X(s,x,y)) \land (X(s,y,x) \lor \neg X(s,y,x)))))$ prem.
174 | $\forall X(\boldsymbol{P}(X) \to \forall x \forall y((A(x) \land A(y)) \to (X(s,x,y) \lor X(s,y,x))))$ prem.
175 | $\forall X(\boldsymbol{P}(X) \to \forall x \forall y \forall z((A(x) \land A(y) \land A(z)) \to ((X(s,x,y) \land X(s,y,z)) \to X(s,x,z))))$ prem.
176 | $\forall X(\boldsymbol{P}(X) \to \forall x \forall y((A(x) \land A(y)) \to (\forall w(H(w) \to (X(w,x,y) \land \neg X(w,y,x))) \to (X(s,x,y) \land \neg X(s,y,x)))))$ prem.
177 | $\forall X \forall Y((\boldsymbol{P}(X) \land \boldsymbol{P}(Y)) \to \forall x \forall y((A(x) \land A(y)) \to (\forall w(H(w) \to ((X(w,x,y) \leftrightarrow Y(w,x,y)) \land (X(w,y,x) \leftrightarrow Y(w,y,x)))) \to ((X(s,x,y) \leftrightarrow Y(s,x,y)) \land (X(s,y,x) \leftrightarrow Y(s,y,x))))))$ prem.
178 | | $\neg \exists w(H(w) \land \forall X(\boldsymbol{P}(X) \to \forall x \forall y((A(x) \land A(y)) \to ((X(w,x,y) \land \neg X(w,y,x)) \to (X(s,x,y) \land \neg X(s,y,x))))) \land \forall u(H(u) \to (\forall X(\boldsymbol{P}(X) \to \forall x \forall y((A(x) \land A(y)) \to ((X(u,x,y) \land \neg X(u,y,x)) \to (X(s,x,y) \land \neg X(s,y,x))))) \to u = w)))$ prem.
179 | | $\boldsymbol{P}(R_{1\text{-}1}) \to \forall x \forall y((A(x) \land A(y)) \to (\forall w(H(w) \to (R_{1\text{-}1}(w,x,y) \land \neg R_{1\text{-}1}(w,y,x))) \to (R_{1\text{-}1}(s,x,y) \land \neg R_{1\text{-}1}(s,y,x))))$ 176, ($\forall E$)
180 | | $\boldsymbol{P}(R_{1\text{-}1})$ 3, ($\land E$)
181 | | $\forall x \forall y((A(x) \land A(y)) \to (\forall w(H(w) \to (R_{1\text{-}1}(w,x,y) \land \neg R_{1\text{-}1}(w,y,x))) \to (R_{1\text{-}1}(s,x,y) \land \neg R_{1\text{-}1}(s,y,x))))$ 179, 180, ($\to E$)
182 | | $(A(a) \land A(b)) \to (\forall w(H(w) \to (R_{1\text{-}1}(w,a,b) \land \neg R_{1\text{-}1}(w,b,a))) \to (R_{1\text{-}1}(s,a,b) \land \neg R_{1\text{-}1}(s,b,a)))$ 181, ($\forall E$)
183 | | $A(a) \land A(b)$ 2, ($\land E$)
184 | | $\forall w(H(w) \to (R_{1\text{-}1}(w,a,b) \land \neg R_{1\text{-}1}(w,b,a))) \to (R_{1\text{-}1}(s,a,b) \land \neg R_{1\text{-}1}(s,b,a))$ 182, 183, ($\to E$)
185 | | | $\neg \forall w(H(w) \to (R_{1\text{-}1}(w,a,b) \land \neg R_{1\text{-}1}(w,b,a)))$ prem.
186 | | | $\exists w \neg(H(w) \to (R_{1\text{-}1}(w,a,b) \land \neg R_{1\text{-}1}(w,b,a)))$ 185, (rep.)
187 | | | | $\neg(H(h) \to (R_{1\text{-}1}(h,a,b) \land \neg R_{1\text{-}1}(h,b,a)))$ prem.
188 | | | | $\forall x(H(x) \to (x = p \lor x = q))$ 1, ($\land E$)
189 | | | | $H(h) \to (h = p \lor h = q)$ 188, ($\forall E$)
190 | | | | | $H(h)$ prem.
191 | | | | | $h = p \lor h = q$ 189 190, ($\to E$)
192 | | | | | | $h = p$ prem.
193 | | | | | | $R_{1\text{-}1}(p,a,b) \land \neg R_{1\text{-}1}(p,b,a)$ 4, ($\land E$)
194 | | | | | | $R_{1\text{-}1}(h,a,b) \land \neg R_{1\text{-}1}(h,b,a)$ 192, 193, ($=E$)
195 | | | | | $(h = p) \to R_{1\text{-}1}(h,a,b) \land \neg R_{1\text{-}1}(h,b,a)$ 192, 194, ($\to I$)
196 | | | | | | $h = q$ prem.
197 | | | | | | $R_{1\text{-}1}(q,a,b) \land \neg R_{1\text{-}1}(q,b,a)$ 4, ($\land E$)
198 | | | | | | $R_{1\text{-}1}(h,a,b) \land \neg R_{1\text{-}1}(h,b,a)$ 196, 197, ($=E$)
199 | | | | | $(h = q) \to R_{1\text{-}1}(h,a,b) \land \neg R_{1\text{-}1}(h,b,a)$ 196, 198, ($\to I$)
200 | | | | | $R_{1\text{-}1}(h,a,b) \land \neg R_{1\text{-}1}(h,b,a)$ 191, 195, 199, ($\lor E$)
201 | | | | $H(h) \to (R_{1\text{-}1}(h,a,b) \land \neg R_{1\text{-}1}(h,b,a))$ 190, 200, ($\to I$)
202 | | | | $\bot$ 187, 201, ($\neg E$)
203 | | | $\bot$ 186, 202, ($\exists E$)
204 | | $\forall w(H(w) \to (R_{1\text{-}1}(w,a,b) \land \neg R_{1\text{-}1}(w,b,a)))$ 185, 203, (DNE)
205 | | $R_{1\text{-}1}(s,a,b) \land \neg R_{1\text{-}1}(s,b,a)$ 184, 204, ($\to E$)
206 | | $R_{1\text{-}1}(s,b,c) \land \neg R_{1\text{-}1}(s,c,b)$ 4, (similar procedure using unanimity 179–205 [SPU])
207 | | $R_{1\text{-}1}(s,a,c) \land \neg R_{1\text{-}1}(s,c,a)$ 4, (SPU)
208 | | $R_{1\text{-}2}(s,a,b) \land \neg R_{1\text{-}2}(s,b,a)$ 5, (SPU)
209 | | $R_{1\text{-}2}(s,a,c) \land \neg R_{1\text{-}2}(s,c,a)$ 5, (SPU)
210 | | $R_{1\text{-}3}(s,b,c) \land \neg R_{1\text{-}3}(s,c,b)$ 6, (SPU)
211 | | $R_{1\text{-}3}(s,a,c) \land \neg R_{1\text{-}3}(s,c,a)$ 6, (SPU)
212 | | $R_{1\text{-}4}(s,b,c) \land \neg R_{1\text{-}4}(s,c,b)$ 7, (SPU)
213 | | $R_{1\text{-}5}(s,a,b) \land \neg R_{1\text{-}5}(s,b,a)$ 8, (SPU)
214 | | $R_{1\text{-}7}(s,b,c) \land \neg R_{1\text{-}7}(s,c,b)$ 10, (SPU)
215 | | $R_{1\text{-}7}(s,a,c) \land \neg R_{1\text{-}7}(s,c,a)$ 10, (SPU)
216 | | $R_{1\text{-}10}(s,a,b) \land \neg R_{1\text{-}10}(s,b,a)$ 13, (SPU)
217 | | $R_{1\text{-}10}(s,a,c) \land \neg R_{1\text{-}10}(s,c,a)$ 13, (SPU)
218 | | $R_{1\text{-}11}(s,a,b) \land \neg R_{1\text{-}11}(s,b,a)$ 14, (SPU)
219 | | $R_{1\text{-}12}(s,b,c) \land \neg R_{1\text{-}12}(s,c,b)$ 15, (SPU)
220 | | $R_{2\text{-}1}(s,a,b) \land \neg R_{2\text{-}1}(s,b,a)$ 17, (SPU)
221 | | $R_{2\text{-}1}(s,a,c) \land \neg R_{2\text{-}1}(s,c,a)$ 17, (SPU)
222 | | $R_{2\text{-}2}(s,a,b) \land \neg R_{2\text{-}2}(s,b,a)$ 18, (SPU)
223 | | $\neg R_{2\text{-}2}(s,b,c) \land R_{2\text{-}2}(s,c,b)$ 18, (SPU)
224 | | $R_{2\text{-}2}(s,a,c) \land \neg R_{2\text{-}2}(s,c,a)$ 18, (SPU)
225 | | $R_{2\text{-}3}(s,a,c) \land \neg R_{2\text{-}3}(s,c,a)$ 19, (SPU)
226 | | $R_{2\text{-}5}(s,a,b) \land \neg R_{2\text{-}5}(s,b,a)$ 21, (SPU)
227 | | $\neg R_{2\text{-}5}(s,b,c) \land R_{2\text{-}5}(s,c,b)$ 21, (SPU)
228 | | $\neg R_{2\text{-}6}(s,b,c) \land R_{2\text{-}6}(s,c,b)$ 22, (SPU)
229 | | $R_{2\text{-}7}(s,a,c) \land \neg R_{2\text{-}7}(s,c,a)$ 23, (SPU)
230 | | $\neg R_{2\text{-}8}(s,b,c) \land R_{2\text{-}8}(s,c,b)$ 24, (SPU)
231 | | $R_{2\text{-}10}(s,a,b) \land \neg R_{2\text{-}10}(s,b,a)$ 26, (SPU)
232 | | $R_{2\text{-}10}(s,a,c) \land \neg R_{2\text{-}10}(s,c,a)$ 26, (SPU)
233 | | $R_{2\text{-}11}(s,a,b) \land \neg R_{2\text{-}11}(s,b,a)$ 27, (SPU)
234 | | $\neg R_{2\text{-}11}(s,b,c) \land R_{2\text{-}11}(s,c,b)$ 27, (SPU)
235 | | $R_{3\text{-}1}(s,b,c) \land \neg R_{3\text{-}1}(s,c,b)$ 30, (SPU)
236 | | $R_{3\text{-}1}(s,a,c) \land \neg R_{3\text{-}1}(s,c,a)$ 30, (SPU)
237 | | $R_{3\text{-}2}(s,a,c) \land \neg R_{3\text{-}2}(s,c,a)$ 31, (SPU)
238 | | $\neg R_{3\text{-}3}(s,a,b) \land R_{3\text{-}3}(s,b,a)$ 32, (SPU)
239 | | $R_{3\text{-}3}(s,b,c) \land \neg R_{3\text{-}3}(s,c,b)$ 32, (SPU)
240 | | $R_{3\text{-}3}(s,a,c) \land \neg R_{3\text{-}3}(s,c,a)$ 32, (SPU)
241 | | $\neg R_{3\text{-}4}(s,a,b) \land R_{3\text{-}4}(s,b,a)$ 33, (SPU)
242 | | $R_{3\text{-}4}(s,b,c) \land \neg R_{3\text{-}4}(s,c,b)$ 33, (SPU)
243 | | $\neg R_{3\text{-}6}(s,a,b) \land R_{3\text{-}6}(s,b,a)$ 35, (SPU)
244 | | $R_{3\text{-}7}(s,b,c) \land \neg R_{3\text{-}7}(s,c,b)$ 36, (SPU)
245 | | $R_{3\text{-}7}(s,a,c) \land \neg R_{3\text{-}7}(s,c,a)$ 36, (SPU)
246 | | $\neg R_{3\text{-}9}(s,a,b) \land R_{3\text{-}9}(s,b,a)$ 38, (SPU)
247 | | $R_{3\text{-}10}(s,a,c) \land \neg R_{3\text{-}10}(s,c,a)$ 39, (SPU)
248 | | $\neg R_{3\text{-}12}(s,a,b) \land R_{3\text{-}12}(s,b,a)$ 41, (SPU)
249 | | $R_{3\text{-}12}(s,b,c) \land \neg R_{3\text{-}12}(s,c,b)$ 41, (SPU)
250 | | $R_{4\text{-}1}(s,b,c) \land \neg R_{4\text{-}1}(s,c,b)$ 43, (SPU)
251 | | $\neg R_{4\text{-}3}(s,a,b) \land R_{4\text{-}3}(s,b,a)$ 45, (SPU)
252 | | $R_{4\text{-}3}(s,b,c) \land \neg R_{4\text{-}3}(s,c,b)$ 45, (SPU)
253 | | $\neg R_{4\text{-}4}(s,a,b) \land R_{4\text{-}4}(s,b,a)$ 46, (SPU)
254 | | $R_{4\text{-}4}(s,b,c) \land \neg R_{4\text{-}4}(s,c,b)$ 46, (SPU)
255 | | $\neg R_{4\text{-}4}(s,a,c) \land R_{4\text{-}4}(s,c,a)$ 46, (SPU)
256 | | $\neg R_{4\text{-}5}(s,a,c) \land R_{4\text{-}5}(s,c,a)$ 47, (SPU)
257 | | $\neg R_{4\text{-}6}(s,a,b) \land R_{4\text{-}6}(s,b,a)$ 48, (SPU)
258 | | $\neg R_{4\text{-}6}(s,a,c) \land R_{4\text{-}6}(s,c,a)$ 48, (SPU)
259 | | $R_{4\text{-}7}(s,b,c) \land \neg R_{4\text{-}7}(s,c,b)$ 49, (SPU)
260 | | $\neg R_{4\text{-}8}(s,a,c) \land R_{4\text{-}8}(s,c,a)$ 50, (SPU)
261 | | $\neg R_{4\text{-}9}(s,a,b) \land R_{4\text{-}9}(s,b,a)$ 51, (SPU)
262 | | $\neg R_{4\text{-}9}(s,a,c) \land R_{4\text{-}9}(s,c,a)$ 51, (SPU)
263 | | $\neg R_{4\text{-}12}(s,a,b) \land R_{4\text{-}12}(s,b,a)$ 54, (SPU)
264 | | $R_{4\text{-}12}(s,b,c) \land \neg R_{4\text{-}12}(s,c,b)$ 54, (SPU)
265 | | $R_{5\text{-}1}(s,a,b) \land \neg R_{5\text{-}1}(s,b,a)$ 56, (SPU)
266 | | $R_{5\text{-}2}(s,a,b) \land \neg R_{5\text{-}2}(s,b,a)$ 57, (SPU)
267 | | $\neg R_{5\text{-}2}(s,b,c) \land R_{5\text{-}2}(s,c,b)$ 57, (SPU)
268 | | $\neg R_{5\text{-}4}(s,a,c) \land R_{5\text{-}4}(s,c,a)$ 59, (SPU)
269 | | $R_{5\text{-}5}(s,a,b) \land \neg R_{5\text{-}5}(s,b,a)$ 60, (SPU)
270 | | $\neg R_{5\text{-}5}(s,b,c) \land R_{5\text{-}5}(s,c,b)$ 60, (SPU)
271 | | $\neg R_{5\text{-}5}(s,a,c) \land R_{5\text{-}5}(s,c,a)$ 60, (SPU)
272 | | $\neg R_{5\text{-}6}(s,b,c) \land R_{5\text{-}6}(s,c,b)$ 61, (SPU)
273 | | $\neg R_{5\text{-}6}(s,a,c) \land R_{5\text{-}6}(s,c,a)$ 61, (SPU)
274 | | $\neg R_{5\text{-}8}(s,b,c) \land R_{5\text{-}8}(s,c,b)$ 63, (SPU)
275 | | $\neg R_{5\text{-}8}(s,a,c) \land R_{5\text{-}8}(s,c,a)$ 63, (SPU)
276 | | $\neg R_{5\text{-}9}(s,a,c) \land R_{5\text{-}9}(s,c,a)$ 64, (SPU)
277 | | $R_{5\text{-}10}(s,a,b) \land \neg R_{5\text{-}10}(s,b,a)$ 65, (SPU)
278 | | $R_{5\text{-}11}(s,a,b) \land \neg R_{5\text{-}11}(s,b,a)$ 66, (SPU)
279 | | $\neg R_{5\text{-}11}(s,b,c) \land R_{5\text{-}11}(s,c,b)$ 66, (SPU)
280 | | $\neg R_{6\text{-}2}(s,b,c) \land R_{6\text{-}2}(s,c,b)$ 70, (SPU)
281 | | $\neg R_{6\text{-}3}(s,a,b) \land R_{6\text{-}3}(s,b,a)$ 71, (SPU)
282 | | $\neg R_{6\text{-}4}(s,a,b) \land R_{6\text{-}4}(s,b,a)$ 72, (SPU)
283 | | $\neg R_{6\text{-}4}(s,a,c) \land R_{6\text{-}4}(s,c,a)$ 72, (SPU)
284 | | $\neg R_{6\text{-}5}(s,b,c) \land R_{6\text{-}5}(s,c,b)$ 73, (SPU)
285 | | $\neg R_{6\text{-}5}(s,a,c) \land R_{6\text{-}5}(s,c,a)$ 73, (SPU)
286 | | $\neg R_{6\text{-}6}(s,a,b) \land R_{6\text{-}6}(s,b,a)$ 74, (SPU)
287 | | $\neg R_{6\text{-}6}(s,b,c) \land R_{6\text{-}6}(s,c,b)$ 74, (SPU)
288 | | $\neg R_{6\text{-}6}(s,a,c) \land R_{6\text{-}6}(s,c,a)$ 74, (SPU)
289 | | $\neg R_{6\text{-}8}(s,b,c) \land R_{6\text{-}8}(s,c,b)$ 76, (SPU)
290 | | $\neg R_{6\text{-}8}(s,a,c) \land R_{6\text{-}8}(s,c,a)$ 76, (SPU)
291 | | $\neg R_{6\text{-}9}(s,a,b) \land R_{6\text{-}9}(s,b,a)$ 77, (SPU)
292 | | $\neg R_{6\text{-}9}(s,a,c) \land R_{6\text{-}9}(s,c,a)$ 77, (SPU)

293 | | $\neg R_{6-11}(s,b,c) \wedge R_{6-11}(s,c,b)$ 79, (*SPU*)
294 | | $\neg R_{6-12}(s,a,b) \wedge R_{6-12}(s,b,a)$ 80, (*SPU*)
295 | | $R_{7-1}(s,b,c) \wedge \neg R_{7-1}(s,c,b)$ 82, (*SPU*)
296 | | $R_{7-1}(s,a,c) \wedge \neg R_{7-1}(s,c,a)$ 82, (*SPU*)
297 | | $R_{7-2}(s,a,c) \wedge \neg R_{7-2}(s,c,a)$ 83, (*SPU*)
298 | | $R_{7-3}(s,b,c) \wedge \neg R_{7-3}(s,c,b)$ 84, (*SPU*)
299 | | $R_{7-3}(s,a,c) \wedge \neg R_{7-3}(s,c,a)$ 84, (*SPU*)
300 | | $R_{7-4}(s,b,c) \wedge \neg R_{7-4}(s,c,b)$ 85, (*SPU*)
301 | | $R_{7-7}(s,b,c) \wedge \neg R_{7-7}(s,c,b)$ 88, (*SPU*)
302 | | $R_{7-7}(s,a,c) \wedge \neg R_{7-7}(s,c,a)$ 88, (*SPU*)
303 | | $R_{7-10}(s,a,c) \wedge \neg R_{7-10}(s,c,a)$ 91, (*SPU*)
304 | | $R_{7-12}(s,b,c) \wedge \neg R_{7-12}(s,c,b)$ 93, (*SPU*)
305 | | $\neg R_{8-2}(s,b,c) \wedge R_{8-2}(s,c,b)$ 96, (*SPU*)
306 | | $\neg R_{8-4}(s,a,c) \wedge R_{8-4}(s,c,a)$ 98, (*SPU*)
307 | | $\neg R_{8-5}(s,b,c) \wedge R_{8-5}(s,c,b)$ 99, (*SPU*)
308 | | $\neg R_{8-5}(s,a,c) \wedge R_{8-5}(s,c,a)$ 99, (*SPU*)
309 | | $\neg R_{8-6}(s,b,c) \wedge R_{8-6}(s,c,b)$ 100, (*SPU*)
310 | | $\neg R_{8-6}(s,a,c) \wedge R_{8-6}(s,c,a)$ 100, (*SPU*)
311 | | $\neg R_{8-8}(s,b,c) \wedge R_{8-8}(s,c,b)$ 102, (*SPU*)
312 | | $\neg R_{8-8}(s,a,c) \wedge R_{8-8}(s,c,a)$ 102, (*SPU*)
313 | | $\neg R_{8-9}(s,a,c) \wedge R_{8-9}(s,c,a)$ 103, (*SPU*)
314 | | $\neg R_{8-11}(s,b,c) \wedge R_{8-11}(s,c,b)$ 105, (*SPU*)
315 | | $\neg R_{9-3}(s,a,b) \wedge R_{9-3}(s,b,a)$ 110, (*SPU*)
316 | | $\neg R_{9-4}(s,a,b) \wedge R_{9-4}(s,b,a)$ 111, (*SPU*)
317 | | $\neg R_{9-4}(s,a,c) \wedge R_{9-4}(s,c,a)$ 111, (*SPU*)
318 | | $\neg R_{9-5}(s,a,c) \wedge R_{9-5}(s,c,a)$ 112, (*SPU*)
319 | | $\neg R_{9-6}(s,a,b) \wedge R_{9-6}(s,b,a)$ 113, (*SPU*)
320 | | $\neg R_{9-6}(s,a,c) \wedge R_{9-6}(s,c,a)$ 113, (*SPU*)
321 | | $\neg R_{9-8}(s,a,c) \wedge R_{9-8}(s,c,a)$ 115, (*SPU*)
322 | | $\neg R_{9-9}(s,a,b) \wedge R_{9-9}(s,b,a)$ 116, (*SPU*)
323 | | $\neg R_{9-9}(s,a,c) \wedge R_{9-9}(s,c,a)$ 116, (*SPU*)
324 | | $\neg R_{9-12}(s,a,b) \wedge R_{9-12}(s,b,a)$ 119, (*SPU*)
325 | | $R_{10-1}(s,a,b) \wedge \neg R_{10-1}(s,b,a)$ 121, (*SPU*)
326 | | $R_{10-1}(s,a,c) \wedge \neg R_{10-1}(s,c,a)$ 121, (*SPU*)
327 | | $R_{10-2}(s,a,b) \wedge \neg R_{10-2}(s,b,a)$ 122, (*SPU*)
328 | | $R_{10-2}(s,a,c) \wedge \neg R_{10-2}(s,c,a)$ 122, (*SPU*)
329 | | $R_{10-3}(s,a,c) \wedge \neg R_{10-3}(s,c,a)$ 123, (*SPU*)
330 | | $R_{10-5}(s,a,b) \wedge \neg R_{10-5}(s,b,a)$ 125, (*SPU*)
331 | | $R_{10-7}(s,a,c) \wedge \neg R_{10-7}(s,c,a)$ 127, (*SPU*)
332 | | $R_{10-10}(s,a,b) \wedge \neg R_{10-10}(s,b,a)$ 130, (*SPU*)
333 | | $R_{10-10}(s,a,c) \wedge \neg R_{10-10}(s,c,a)$ 130, (*SPU*)
334 | | $R_{10-11}(s,a,b) \wedge \neg R_{10-11}(s,b,a)$ 131, (*SPU*)
335 | | $R_{11-1}(s,a,b) \wedge \neg R_{11-1}(s,b,a)$ 134, (*SPU*)
336 | | $R_{11-2}(s,a,b) \wedge \neg R_{11-2}(s,b,a)$ 135, (*SPU*)
337 | | $\neg R_{11-2}(s,b,c) \wedge R_{11-2}(s,c,b)$ 135, (*SPU*)
338 | | $R_{11-5}(s,a,b) \wedge \neg R_{11-5}(s,b,a)$ 138, (*SPU*)
339 | | $\neg R_{11-5}(s,b,c) \wedge R_{11-5}(s,c,b)$ 138, (*SPU*)
340 | | $\neg R_{11-6}(s,b,c) \wedge R_{11-6}(s,c,b)$ 139, (*SPU*)
341 | | $\neg R_{11-8}(s,b,c) \wedge R_{11-8}(s,c,b)$ 141, (*SPU*)
342 | | $R_{11-10}(s,a,b) \wedge \neg R_{11-10}(s,b,a)$ 143, (*SPU*)
343 | | $R_{11-11}(s,a,b) \wedge \neg R_{11-11}(s,b,a)$ 144, (*SPU*)
344 | | $\neg R_{11-11}(s,b,c) \wedge R_{11-11}(s,c,b)$ 144, (*SPU*)
345 | | $R_{12-1}(s,b,c) \wedge \neg R_{12-1}(s,c,b)$ 147, (*SPU*)
346 | | $\neg R_{12-3}(s,a,b) \wedge R_{12-3}(s,b,a)$ 149, (*SPU*)
347 | | $R_{12-3}(s,b,c) \wedge \neg R_{12-3}(s,c,b)$ 149, (*SPU*)
348 | | $\neg R_{12-4}(s,a,b) \wedge R_{12-4}(s,b,a)$ 150, (*SPU*)
349 | | $R_{12-4}(s,b,c) \wedge \neg R_{12-4}(s,c,b)$ 150, (*SPU*)
350 | | $\neg R_{12-6}(s,a,b) \wedge R_{12-6}(s,b,a)$ 152, (*SPU*)
351 | | $R_{12-7}(s,b,c) \wedge \neg R_{12-7}(s,c,b)$ 153, (*SPU*)
352 | | $\neg R_{12-9}(s,a,b) \wedge R_{12-9}(s,b,a)$ 155, (*SPU*)
353 | | $\neg R_{12-12}(s,a,b) \wedge R_{12-12}(s,b,a)$ 158, (*SPU*)
354 | | $R_{12-12}(s,b,c) \wedge \neg R_{12-12}(s,c,b)$ 158, (*SPU*)
355 | | $\boldsymbol{P}(R_{1-2}) \rightarrow \forall x \forall y((A(x) \wedge A(y)) \rightarrow ((R_{1-2}(s,x,y) \vee \neg R_{1-2}(s,x,y)) \wedge (R_{1-2}(s,y,x) \vee \neg R_{1-2}(s,y,x))))$ 173, ($\forall E$)
356 | | $\boldsymbol{P}(R_{1-2})$ 3, ($\wedge E$)
357 | | $\forall x \forall y((A(x) \wedge A(y)) \rightarrow ((R_{1-2}(s,x,y) \vee \neg R_{1-2}(s,x,y)) \wedge (R_{1-2}(s,y,x) \vee \neg R_{1-2}(s,y,x))))$ 355, 356, ($\rightarrow E$)
358 | | $(A(b) \wedge A(c)) \rightarrow ((R_{1-2}(s,b,c) \vee \neg R_{1-2}(s,b,c)) \wedge (R_{1-2}(s,c,b) \vee \neg R_{1-2}(s,c,b)))$ 357, ($\forall E$)
359 | | $A(b) \wedge A(c)$ 2, ($\wedge E$)
360 | | $(R_{1-2}(s,b,c) \vee \neg R_{1-2}(s,b,c)) \wedge (R_{1-2}(s,c,b) \vee \neg R_{1-2}(s,c,b))$ 358, 359, ($\rightarrow E$)
361 | | $R_{1-2}(s,b,c) \vee \neg R_{1-2}(s,b,c)$ 360, ($\wedge E$)
362 | | | $R_{1-2}(s,b,c)$ prem.
363 | | | $R_{1-2}(s,c,b) \vee \neg R_{1-2}(s,c,b)$ 360, ($\wedge E$)
364 | | | | $R_{1-2}(s,c,b)$ prem.
365 | | | | $(\boldsymbol{P}(R_{1-2}) \wedge \boldsymbol{P}(R_{1-5})) \rightarrow \forall x \forall y((A(x) \wedge A(y)) \rightarrow (\forall w(H(w) \rightarrow ((R_{1-2}(w,x,y) \leftrightarrow R_{1-5}(w,x,y)) \wedge (R_{1-2}(w,y,x) \leftrightarrow R_{1-5}(w,y,x)))) \rightarrow ((R_{1-2}(s,x,y) \leftrightarrow R_{1-5}(s,x,y)) \wedge (R_{1-2}(s,y,x) \leftrightarrow R_{1-5}(s,y,x)))))$ 177, ($\forall E$)
366 | | | | $\boldsymbol{P}(R_{1-2}) \wedge \boldsymbol{P}(R_{1-5})$ 3, ($\wedge E$)
367 | | | | $\forall x \forall y((A(x) \wedge A(y)) \rightarrow (\forall w(H(w) \rightarrow ((R_{1-2}(w,x,y) \leftrightarrow R_{1-5}(w,x,y)) \wedge (R_{1-2}(w,y,x) \leftrightarrow R_{1-5}(w,y,x)))) \rightarrow ((R_{1-2}(s,x,y) \leftrightarrow R_{1-5}(s,x,y)) \wedge (R_{1-2}(s,y,x) \leftrightarrow R_{1-5}(s,y,x)))))$ 365, 366, ($\rightarrow E$)
368 | | | | $(A(b) \wedge A(c)) \rightarrow (\forall w(H(w) \rightarrow ((R_{1-2}(w,b,c) \leftrightarrow R_{1-5}(w,b,c)) \wedge (R_{1-2}(w,c,b) \leftrightarrow R_{1-5}(w,c,b)))) \rightarrow ((R_{1-2}(s,b,c) \leftrightarrow R_{1-5}(s,b,c)) \wedge (R_{1-2}(s,c,b) \leftrightarrow R_{1-5}(s,c,b))))$ 367, ($\forall E$)
369 | | | | $A(b) \wedge A(c)$ 2, ($\wedge E$)
370 | | | | $\forall w(H(w) \rightarrow ((R_{1-2}(w,b,c) \leftrightarrow R_{1-5}(w,b,c)) \wedge (R_{1-2}(w,c,b) \leftrightarrow R_{1-5}(w,c,b)))) \rightarrow ((R_{1-2}(s,b,c) \leftrightarrow R_{1-5}(s,b,c)) \wedge (R_{1-2}(s,c,b) \leftrightarrow R_{1-5}(s,c,b)))$ 368, 369, ($\rightarrow E$)
371 | | | | | $\neg \forall w(H(w) \rightarrow ((R_{1-2}(w,b,c) \leftrightarrow R_{1-5}(w,b,c)) \wedge (R_{1-2}(w,c,b) \leftrightarrow R_{1-5}(w,c,b))))$ prem.
372 | | | | | $\exists w \neg(H(w) \rightarrow ((R_{1-2}(w,b,c) \leftrightarrow R_{1-5}(w,b,c)) \wedge (R_{1-2}(w,c,b) \leftrightarrow R_{1-5}(w,c,b))))$ 371, (rep.)
373 | | | | | | $\neg(H(h) \rightarrow ((R_{1-2}(h,b,c) \leftrightarrow R_{1-5}(h,b,c)) \wedge (R_{1-2}(h,c,b) \leftrightarrow R_{1-5}(h,c,b))))$ prem.
374 | | | | | | $\forall x(H(x) \rightarrow (x = p \vee x = q))$ 1, ($\wedge E$)
375 | | | | | | $H(h) \rightarrow (h = p \vee h = q)$ 374, ($\forall E$)
376 | | | | | | | $H(h)$ prem.
377 | | | | | | | $h = p \vee h = q$ 375, 376, ($\rightarrow E$)
378 | | | | | | | | $h = p$ prem.
379 | | | | | | | | | $R_{1-2}(p,b,c)$ prem.
380 | | | | | | | | | $R_{1-5}(p,b,c)$ 8, ($\wedge E$)
381 | | | | | | | | $R_{1-2}(p,b,c) \rightarrow R_{1-5}(p,b,c)$ 379, 380, ($\rightarrow I$)
382 | | | | | | | | | $R_{1-5}(p,b,c)$ prem.
383 | | | | | | | | | $R_{1-2}(p,b,c)$ 5, ($\wedge E$)
384 | | | | | | | | $R_{1-5}(p,b,c) \rightarrow R_{1-2}(p,b,c)$ 382, 383, ($\rightarrow I$)
385 | | | | | | | | $R_{1-2}(p,b,c) \leftrightarrow R_{1-5}(p,b,c)$ 381, 384, ($\leftrightarrow I$)
386 | | | | | | | | | $\neg R_{1-2}(p,c,b)$ prem.
387 | | | | | | | | | $\neg R_{1-5}(p,c,b)$ 8, ($\wedge E$)
388 | | | | | | | | $\neg R_{1-2}(p,c,b) \rightarrow \neg R_{1-5}(p,c,b)$ 386, 387, ($\rightarrow I$)
389 | | | | | | | | $R_{1-5}(p,c,b) \rightarrow R_{1-2}(p,c,b)$ 388, (rep.)
390 | | | | | | | | | $\neg R_{1-5}(p,c,b)$ prem.
391 | | | | | | | | | $\neg R_{1-2}(p,c,b)$ 5, ($\wedge E$)
392 | | | | | | | | $\neg R_{1-5}(p,c,b) \rightarrow \neg R_{1-2}(p,c,b)$ 390, 391, ($\rightarrow I$)
393 | | | | | | | | $R_{1-2}(p,c,b) \rightarrow R_{1-5}(p,c,b)$ 392, (rep.)
394 | | | | | | | | $R_{1-2}(p,c,b) \leftrightarrow R_{1-5}(p,c,b)$ 389, 393, ($\leftrightarrow I$)
395 | | | | | | | | $(R_{1-2}(p,b,c) \leftrightarrow R_{1-5}(p,b,c)) \wedge (R_{1-2}(p,c,b) \leftrightarrow R_{1-5}(p,c,b))$ 385, 394, ($\wedge I$)
396 | | | | | | | | $(R_{1-2}(h,b,c) \leftrightarrow R_{1-5}(h,b,c)) \wedge (R_{1-2}(h,c,b) \leftrightarrow R_{1-5}(h,c,b))$ 378, 395, ($=E$)
397 | | | | | | | $(h = p) \rightarrow ((R_{1-2}(h,b,c) \leftrightarrow R_{1-5}(h,b,c)) \wedge (R_{1-2}(h,c,b) \leftrightarrow R_{1-5}(h,c,b)))$ 378, 396, ($\rightarrow I$)
398 | | | | | | | | $h = q$ prem.
399 | | | | | | | | | $\neg R_{1-2}(q,b,c)$ prem.
400 | | | | | | | | | $\neg R_{1-5}(q,b,c)$ 8, ($\wedge E$)
401 | | | | | | | | $\neg R_{1-2}(q,b,c) \rightarrow \neg R_{1-5}(q,b,c)$ 399, 400, ($\rightarrow I$)
402 | | | | | | | | $R_{1-5}(q,b,c) \rightarrow R_{1-2}(q,b,c)$ 401, (rep.)
403 | | | | | | | | | $\neg R_{1-5}(q,b,c)$ prem.
404 | | | | | | | | | $\neg R_{1-2}(q,b,c)$ 5, ($\wedge E$)
405 | | | | | | | | $\neg R_{1-5}(q,b,c) \rightarrow \neg R_{1-2}(q,b,c)$ 403, 404, ($\rightarrow I$)
406 | | | | | | | | $R_{1-2}(q,b,c) \rightarrow R_{1-5}(q,b,c)$ 405, (rep.)
407 | | | | | | | | $R_{1-2}(q,b,c) \leftrightarrow R_{1-5}(q,b,c)$ 402, 406, ($\leftrightarrow I$)
408 | | | | | | | | | $R_{1-2}(q,c,b)$ prem.
409 | | | | | | | | | $R_{1-5}(q,c,b)$ 8, ($\wedge E$)
410 | | | | | | | | $R_{1-2}(q,c,b) \rightarrow R_{1-5}(q,c,b)$ 408, 409, ($\rightarrow I$)
411 | | | | | | | | | $R_{1-5}(q,c,b)$ prem.
412 | | | | | | | | | $R_{1-2}(q,c,b)$ 5, ($\wedge E$)
413 | | | | | | | | $R_{1-5}(q,c,b) \rightarrow R_{1-2}(q,c,b)$ 411, 412, ($\rightarrow I$)
414 | | | | | | | | $R_{1-2}(q,c,b) \leftrightarrow R_{1-5}(q,c,b)$ 410, 413, ($\leftrightarrow I$)
415 | | | | | | | | $(R_{1-2}(q,b,c) \leftrightarrow R_{1-5}(q,b,c)) \wedge (R_{1-2}(q,c,b) \leftrightarrow R_{1-5}(q,c,b))$ 407, 414, ($\wedge I$)
416 | | | | | | | | $(R_{1-2}(h,b,c) \leftrightarrow R_{1-5}(h,b,c)) \wedge (R_{1-2}(h,c,b) \leftrightarrow R_{1-5}(h,c,b))$ 398, 415, ($=E$)
417 | | | | | | | $(h = q) \rightarrow ((R_{1-2}(h,b,c) \leftrightarrow R_{1-5}(h,b,c)) \wedge (R_{1-2}(h,c,b) \leftrightarrow R_{1-5}(h,c,b)))$ 398, 416, ($\rightarrow I$)
418 | | | | | | | $(R_{1-2}(h,b,c) \leftrightarrow R_{1-5}(h,b,c)) \wedge (R_{1-2}(h,c,b) \leftrightarrow R_{1-5}(h,c,b))$ 377, 397, 417, ($\vee E$)
419 | | | | | | $H(h) \rightarrow ((R_{1-2}(h,b,c) \leftrightarrow R_{1-5}(h,b,c)) \wedge (R_{1-2}(h,c,b) \leftrightarrow R_{1-5}(h,c,b)))$ 376, 418, ($\rightarrow I$)
420 | | | | | | $\bot$ 373, 419, ($\neg E$)
421 | | | | | $\bot$ 372, 420, ($\exists E$)
422 | | | | $\forall w(H(w) \rightarrow ((R_{1-2}(w,b,c) \leftrightarrow R_{1-5}(w,b,c)) \wedge (R_{1-2}(w,c,b) \leftrightarrow R_{1-5}(w,c,b))))$ 371, 421, (*DNE*)
423 | | | | $(R_{1-2}(s,b,c) \leftrightarrow R_{1-5}(s,b,c)) \wedge (R_{1-2}(s,c,b) \leftrightarrow R_{1-5}(s,c,b))$ 370, 422, ($\rightarrow E$)
424 | | | | $R_{1-2}(s,b,c) \leftrightarrow R_{1-5}(s,b,c)$ 423, ($\wedge E$)
425 | | | | $R_{1-5}(s,b,c)$ 362, 424, ($\leftrightarrow E$)
426 | | | | $R_{1-2}(s,c,b) \leftrightarrow R_{1-5}(s,c,b)$ 423, ($\wedge E$)
427 | | | | $R_{1-5}(s,c,b)$ 364, 426, ($\leftrightarrow E$)
428 | | | | $R_{1-5}(s,b,c) \wedge R_{1-5}(s,c,b)$ 425, 427, ($\wedge I$)
429 | | | | $\boldsymbol{P}(R_{1-5}) \rightarrow \forall x \forall y \forall z((A(x) \wedge A(y) \wedge A(z)) \rightarrow ((R_{1-5}(s,x,y) \wedge R_{1-5}(s,y,z)) \rightarrow R_{1-5}(s,x,z)))$ 175, ($\forall E$)
430 | | | | $\boldsymbol{P}(R_{1-5})$ 3, ($\wedge E$)
431 | | | | $\forall x \forall y \forall z((A(x) \wedge A(y) \wedge A(z)) \rightarrow ((R_{1-5}(s,x,y) \wedge R_{1-5}(s,y,z)) \rightarrow R_{1-5}(s,x,z)))$ 429, 430, ($\rightarrow E$)
432 | | | | | $\neg(R_{1-5}(s,a,c) \wedge \neg R_{1-5}(s,c,a))$ prem.
433 | | | | | $\neg R_{1-5}(s,a,c) \vee R_{1-5}(s,c,a)$ 432, (rep.)
434 | | | | | | $\neg R_{1-5}(s,a,c)$ prem.
435 | | | | | | $(A(a) \wedge A(b) \wedge A(c)) \rightarrow ((R_{1-5}(s,a,b) \wedge R_{1-5}(s,b,c)) \rightarrow R_{1-5}(s,a,c))$ 431, ($\forall E$)
436 | | | | | | $A(a) \wedge A(b) \wedge A(c)$ 2, ($\wedge E$)
437 | | | | | | $(R_{1-5}(s,a,b) \wedge R_{1-5}(s,b,c)) \rightarrow R_{1-5}(s,a,c)$ 435, 436, ($\rightarrow E$)
438 | | | | | | $R_{1-5}(s,a,b)$ 213, ($\wedge E$)
439 | | | | | | $R_{1-5}(s,b,c)$ 428, ($\wedge E$)
440 | | | | | | $R_{1-5}(s,a,b) \wedge R_{1-5}(s,b,c)$ 438, 439, ($\wedge I$)
441 | | | | | | $R_{1-5}(s,a,c)$ 437, 440, ($\rightarrow E$)
442 | | | | | | $\bot$ 434, 441, ($\neg E$)
443 | | | | | $\neg R_{1-5}(s,a,c) \rightarrow \bot$ 434, 442, ($\rightarrow I$)

444 | | | | | | $\underline{R_{1\text{-}5}(s,c,a)}$ prem.
445 | | | | | | $(A(b) \land A(c) \land A(a)) \to ((R_{1\text{-}5}(s,b,c) \land R_{1\text{-}5}(s,c,a)) \to R_{1\text{-}5}(s,b,a))$ 431, ($\forall E$)
446 | | | | | | $A(b) \land A(c) \land A(a)$ 2, ($\land E$)
447 | | | | | | $(R_{1\text{-}5}(s,b,c) \land R_{1\text{-}5}(s,c,a)) \to R_{1\text{-}5}(s,b,a)$ 445, 446, ($\to E$)
448 | | | | | | $R_{1\text{-}5}(s,b,c)$ 428, ($\land E$)
449 | | | | | | $R_{1\text{-}5}(s,b,c) \land R_{1\text{-}5}(s,c,a)$ 448, 444, ($\land I$)
450 | | | | | | $R_{1\text{-}5}(s,b,a)$ 447, 449, ($\to E$)
451 | | | | | | $\neg R_{1\text{-}5}(s,b,a)$ 213, ($\land E$)
452 | | | | | | $\bot$ 450, 451, ($\neg E$)
453 | | | | | $R_{1\text{-}5}(s,c,a) \to \bot$ 444, 452, ($\to I$)
454 | | | | | $\bot$ 433, 443, 453, ($\lor E$)
455 | | | | $R_{1\text{-}5}(s,a,c) \land \neg R_{1\text{-}5}(s,c,a)$ 432, 454, (*DNE*)
456 | | | | $\neg R_{3\text{-}6}(s,a,b) \land R_{3\text{-}6}(s,b,a)$ 243, (rep.)
457 | | | | $R_{3\text{-}6}(s,b,c) \land R_{3\text{-}6}(s,c,b)$ 428, (similar procedure using IIA 365–428 [*SPI*])
458 | | | | $\neg R_{3\text{-}6}(s,a,c) \land R_{3\text{-}6}(s,c,a)$ 456, 457, (similar procedure using transitivity 429–455 [*SPT*])
459 | | | | $R_{3\text{-}6}(s,a,c) \land \neg R_{3\text{-}6}(s,c,a)$ 455, (*SPI*)
460 | | | | $\neg R_{3\text{-}6}(s,a,c)$ 458, ($\land E$)
461 | | | | $R_{3\text{-}6}(s,a,c)$ 459, ($\land E$)
462 | | | | $\bot$ 460, 461, ($\neg E$)
463 | | | $R_{1\text{-}2}(s,c,b) \to \bot$ 364, 462, ($\to I$)
464 | | | | $\underline{\neg R_{1\text{-}2}(s,c,b)}$ prem.
465 | | | | $R_{1\text{-}5}(s,b,c) \land \neg R_{1\text{-}5}(s,c,b)$ 362, 464, (*SPI*)
466 | | | | $R_{1\text{-}6}(s,b,c) \land \neg R_{1\text{-}6}(s,c,b)$ 362, 464, (*SPI*)
467 | | | | $R_{1\text{-}8}(s,b,c) \land \neg R_{1\text{-}8}(s,c,b)$ 362, 464, (*SPI*)
468 | | | | $R_{1\text{-}11}(s,b,c) \land \neg R_{1\text{-}11}(s,c,b)$ 362, 464, (*SPI*)
469 | | | | $R_{3\text{-}2}(s,b,c) \land \neg R_{3\text{-}2}(s,c,b)$ 362, 464, (*SPI*)
470 | | | | $R_{3\text{-}5}(s,b,c) \land \neg R_{3\text{-}5}(s,c,b)$ 362, 464, (*SPI*)
471 | | | | $R_{3\text{-}6}(s,b,c) \land \neg R_{3\text{-}6}(s,c,b)$ 362, 464, (*SPI*)
472 | | | | $R_{3\text{-}8}(s,b,c) \land \neg R_{3\text{-}8}(s,c,b)$ 362, 464, (*SPI*)
473 | | | | $R_{3\text{-}11}(s,b,c) \land \neg R_{3\text{-}11}(s,c,b)$ 362, 464, (*SPI*)
474 | | | | $R_{4\text{-}2}(s,b,c) \land \neg R_{4\text{-}2}(s,c,b)$ 362, 464, (*SPI*)
475 | | | | $R_{4\text{-}5}(s,b,c) \land \neg R_{4\text{-}5}(s,c,b)$ 362, 464, (*SPI*)
476 | | | | $R_{4\text{-}6}(s,b,c) \land \neg R_{4\text{-}6}(s,c,b)$ 362, 464, (*SPI*)
477 | | | | $R_{4\text{-}8}(s,b,c) \land \neg R_{4\text{-}8}(s,c,b)$ 362, 464, (*SPI*)
478 | | | | $R_{4\text{-}11}(s,b,c) \land \neg R_{4\text{-}11}(s,c,b)$ 362, 464, (*SPI*)
479 | | | | $R_{7\text{-}2}(s,b,c) \land \neg R_{7\text{-}2}(s,c,b)$ 362, 464, (*SPI*)
480 | | | | $R_{7\text{-}5}(s,b,c) \land \neg R_{7\text{-}5}(s,c,b)$ 362, 464, (*SPI*)
481 | | | | $R_{7\text{-}6}(s,b,c) \land \neg R_{7\text{-}6}(s,c,b)$ 362, 464, (*SPI*)
482 | | | | $R_{7\text{-}8}(s,b,c) \land \neg R_{7\text{-}8}(s,c,b)$ 362, 464, (*SPI*)
483 | | | | $R_{7\text{-}11}(s,b,c) \land \neg R_{7\text{-}11}(s,c,b)$ 362, 464, (*SPI*)
484 | | | | $R_{12\text{-}2}(s,b,c) \land \neg R_{12\text{-}2}(s,c,b)$ 362, 464, (*SPI*)
485 | | | | $R_{12\text{-}5}(s,b,c) \land \neg R_{12\text{-}5}(s,c,b)$ 362, 464, (*SPI*)
486 | | | | $R_{12\text{-}6}(s,b,c) \land \neg R_{12\text{-}6}(s,c,b)$ 362, 464, (*SPI*)
487 | | | | $R_{12\text{-}8}(s,b,c) \land \neg R_{12\text{-}8}(s,c,b)$ 362, 464, (*SPI*)
488 | | | | $R_{12\text{-}11}(s,b,c) \land \neg R_{12\text{-}11}(s,c,b)$ 362, 464, (*SPI*)
489 | | | | $R_{1\text{-}5}(s,a,c) \land \neg R_{1\text{-}5}(s,c,a)$ 213, 465, (*SPT*)
490 | | | | $R_{1\text{-}4}(s,a,c) \land \neg R_{1\text{-}4}(s,c,a)$ 489, (*SPI*)
491 | | | | $R_{1\text{-}6}(s,a,c) \land \neg R_{1\text{-}6}(s,c,a)$ 489, (*SPI*)
492 | | | | $R_{1\text{-}8}(s,a,c) \land \neg R_{1\text{-}8}(s,c,a)$ 489, (*SPI*)
493 | | | | $R_{1\text{-}9}(s,a,c) \land \neg R_{1\text{-}9}(s,c,a)$ 489, (*SPI*)
494 | | | | $R_{2\text{-}4}(s,a,c) \land \neg R_{2\text{-}4}(s,c,a)$ 489, (*SPI*)
495 | | | | $R_{2\text{-}5}(s,a,c) \land \neg R_{2\text{-}5}(s,c,a)$ 489, (*SPI*)
496 | | | | $R_{2\text{-}6}(s,a,c) \land \neg R_{2\text{-}6}(s,c,a)$ 489, (*SPI*)
497 | | | | $R_{2\text{-}8}(s,a,c) \land \neg R_{2\text{-}8}(s,c,a)$ 489, (*SPI*)
498 | | | | $R_{2\text{-}9}(s,a,c) \land \neg R_{2\text{-}9}(s,c,a)$ 489, (*SPI*)
499 | | | | $R_{3\text{-}4}(s,a,c) \land \neg R_{3\text{-}4}(s,c,a)$ 489, (*SPI*)
500 | | | | $R_{3\text{-}5}(s,a,c) \land \neg R_{3\text{-}5}(s,c,a)$ 489, (*SPI*)
501 | | | | $R_{3\text{-}6}(s,a,c) \land \neg R_{3\text{-}6}(s,c,a)$ 489, (*SPI*)
502 | | | | $R_{3\text{-}8}(s,a,c) \land \neg R_{3\text{-}8}(s,c,a)$ 489, (*SPI*)
503 | | | | $R_{3\text{-}9}(s,a,c) \land \neg R_{3\text{-}9}(s,c,a)$ 489, (*SPI*)
504 | | | | $R_{7\text{-}4}(s,a,c) \land \neg R_{7\text{-}4}(s,c,a)$ 489, (*SPI*)
505 | | | | $R_{7\text{-}5}(s,a,c) \land \neg R_{7\text{-}5}(s,c,a)$ 489, (*SPI*)
506 | | | | $R_{7\text{-}6}(s,a,c) \land \neg R_{7\text{-}6}(s,c,a)$ 489, (*SPI*)
507 | | | | $R_{7\text{-}8}(s,a,c) \land \neg R_{7\text{-}8}(s,c,a)$ 489, (*SPI*)
508 | | | | $R_{7\text{-}9}(s,a,c) \land \neg R_{7\text{-}9}(s,c,a)$ 489, (*SPI*)
509 | | | | $R_{10\text{-}4}(s,a,c) \land \neg R_{10\text{-}4}(s,c,a)$ 489, (*SPI*)
510 | | | | $R_{10\text{-}5}(s,a,c) \land \neg R_{10\text{-}5}(s,c,a)$ 489, (*SPI*)
511 | | | | $R_{10\text{-}6}(s,a,c) \land \neg R_{10\text{-}6}(s,c,a)$ 489, (*SPI*)
512 | | | | $R_{10\text{-}8}(s,a,c) \land \neg R_{10\text{-}8}(s,c,a)$ 489, (*SPI*)
513 | | | | $R_{10\text{-}9}(s,a,c) \land \neg R_{10\text{-}9}(s,c,a)$ 489, (*SPI*)
514 | | | | $R_{2\text{-}6}(s,a,b) \land \neg R_{2\text{-}6}(s,b,a)$ 228, 496, (*SPT*)
515 | | | | $R_{1\text{-}3}(s,a,b) \land \neg R_{1\text{-}3}(s,b,a)$ 514, (*SPI*)
516 | | | | $R_{1\text{-}4}(s,a,b) \land \neg R_{1\text{-}4}(s,b,a)$ 514, (*SPI*)
517 | | | | $R_{1\text{-}6}(s,a,b) \land \neg R_{1\text{-}6}(s,b,a)$ 514, (*SPI*)
518 | | | | $R_{1\text{-}9}(s,a,b) \land \neg R_{1\text{-}9}(s,b,a)$ 514, (*SPI*)
519 | | | | $R_{1\text{-}12}(s,a,b) \land \neg R_{1\text{-}12}(s,b,a)$ 514, (*SPI*)
520 | | | | $R_{2\text{-}3}(s,a,b) \land \neg R_{2\text{-}3}(s,b,a)$ 514, (*SPI*)
521 | | | | $R_{2\text{-}4}(s,a,b) \land \neg R_{2\text{-}4}(s,b,a)$ 514, (*SPI*)
522 | | | | $R_{2\text{-}9}(s,a,b) \land \neg R_{2\text{-}9}(s,b,a)$ 514, (*SPI*)
523 | | | | $R_{2\text{-}12}(s,a,b) \land \neg R_{2\text{-}12}(s,b,a)$ 514, (*SPI*)
524 | | | | $R_{5\text{-}3}(s,a,b) \land \neg R_{5\text{-}3}(s,b,a)$ 514, (*SPI*)
525 | | | | $R_{5\text{-}4}(s,a,b) \land \neg R_{5\text{-}4}(s,b,a)$ 514, (*SPI*)
526 | | | | $R_{5\text{-}6}(s,a,b) \land \neg R_{5\text{-}6}(s,b,a)$ 514, (*SPI*)
527 | | | | $R_{5\text{-}9}(s,a,b) \land \neg R_{5\text{-}9}(s,b,a)$ 514, (*SPI*)
528 | | | | $R_{5\text{-}12}(s,a,b) \land \neg R_{5\text{-}12}(s,b,a)$ 514, (*SPI*)
529 | | | | $R_{10\text{-}3}(s,a,b) \land \neg R_{10\text{-}3}(s,b,a)$ 514, (*SPI*)
530 | | | | $R_{10\text{-}4}(s,a,b) \land \neg R_{10\text{-}4}(s,b,a)$ 514, (*SPI*)
531 | | | | $R_{10\text{-}6}(s,a,b) \land \neg R_{10\text{-}6}(s,b,a)$ 514, (*SPI*)
532 | | | | $R_{10\text{-}9}(s,a,b) \land \neg R_{10\text{-}9}(s,b,a)$ 514, (*SPI*)
533 | | | | $R_{10\text{-}12}(s,a,b) \land \neg R_{10\text{-}12}(s,b,a)$ 514, (*SPI*)
534 | | | | $R_{11\text{-}3}(s,a,b) \land \neg R_{11\text{-}3}(s,b,a)$ 514, (*SPI*)
535 | | | | $R_{11\text{-}4}(s,a,b) \land \neg R_{11\text{-}4}(s,b,a)$ 514, (*SPI*)
536 | | | | $R_{11\text{-}6}(s,a,b) \land \neg R_{11\text{-}6}(s,b,a)$ 514, (*SPI*)
537 | | | | $R_{11\text{-}9}(s,a,b) \land \neg R_{11\text{-}9}(s,b,a)$ 514, (*SPI*)
538 | | | | $R_{11\text{-}12}(s,a,b) \land \neg R_{11\text{-}12}(s,b,a)$ 514, (*SPI*)
539 | | | | $R_{1\text{-}11}(s,a,c) \land \neg R_{1\text{-}11}(s,c,a)$ 218, 468, (*SPT*)
540 | | | | $R_{1\text{-}12}(s,a,c) \land \neg R_{1\text{-}12}(s,c,a)$ 539, (*SPI*)
541 | | | | $R_{1\text{-}13}(s,a,c) \land \neg R_{1\text{-}13}(s,c,a)$ 539, (*SPI*)
542 | | | | $R_{2\text{-}11}(s,a,c) \land \neg R_{2\text{-}11}(s,c,a)$ 539, (*SPI*)
543 | | | | $R_{2\text{-}12}(s,a,c) \land \neg R_{2\text{-}12}(s,c,a)$ 539, (*SPI*)
544 | | | | $R_{2\text{-}13}(s,a,c) \land \neg R_{2\text{-}13}(s,c,a)$ 539, (*SPI*)
545 | | | | $R_{3\text{-}11}(s,a,c) \land \neg R_{3\text{-}11}(s,c,a)$ 539, (*SPI*)
546 | | | | $R_{3\text{-}12}(s,a,c) \land \neg R_{3\text{-}12}(s,c,a)$ 539, (*SPI*)
547 | | | | $R_{3\text{-}13}(s,a,c) \land \neg R_{3\text{-}13}(s,c,a)$ 539, (*SPI*)
548 | | | | $R_{7\text{-}11}(s,a,c) \land \neg R_{7\text{-}11}(s,c,a)$ 539, (*SPI*)
549 | | | | $R_{7\text{-}12}(s,a,c) \land \neg R_{7\text{-}12}(s,c,a)$ 539, (*SPI*)
550 | | | | $R_{7\text{-}13}(s,a,c) \land \neg R_{7\text{-}13}(s,c,a)$ 539, (*SPI*)
551 | | | | $R_{10\text{-}11}(s,a,c) \land \neg R_{10\text{-}11}(s,c,a)$ 539, (*SPI*)
552 | | | | $R_{10\text{-}12}(s,a,c) \land \neg R_{10\text{-}12}(s,c,a)$ 539, (*SPI*)
553 | | | | $R_{10\text{-}13}(s,a,c) \land \neg R_{10\text{-}13}(s,c,a)$ 539, (*SPI*)
554 | | | | $R_{2\text{-}8}(s,a,b) \land \neg R_{2\text{-}8}(s,b,a)$ 230, 497, (*SPT*)
555 | | | | $R_{1\text{-}7}(s,a,b) \land \neg R_{1\text{-}7}(s,b,a)$ 554. (*SPI*)
556 | | | | $R_{1\text{-}8}(s,a,b) \land \neg R_{1\text{-}8}(s,b,a)$ 554. (*SPI*)
557 | | | | $R_{1\text{-}13}(s,a,b) \land \neg R_{1\text{-}13}(s,b,a)$ 554. (*SPI*)
558 | | | | $R_{2\text{-}7}(s,a,b) \land \neg R_{2\text{-}7}(s,b,a)$ 554. (*SPI*)
559 | | | | $R_{2\text{-}13}(s,a,b) \land \neg R_{2\text{-}13}(s,b,a)$ 554. (*SPI*)
560 | | | | $R_{5\text{-}7}(s,a,b) \land \neg R_{5\text{-}7}(s,b,a)$ 554. (*SPI*)
561 | | | | $R_{5\text{-}8}(s,a,b) \land \neg R_{5\text{-}8}(s,b,a)$ 554. (*SPI*)
562 | | | | $R_{5\text{-}13}(s,a,b) \land \neg R_{5\text{-}13}(s,b,a)$ 554. (*SPI*)
563 | | | | $R_{10\text{-}7}(s,a,b) \land \neg R_{10\text{-}7}(s,b,a)$ 554. (*SPI*)
564 | | | | $R_{10\text{-}8}(s,a,b) \land \neg R_{10\text{-}8}(s,b,a)$ 554. (*SPI*)
565 | | | | $R_{10\text{-}13}(s,a,b) \land \neg R_{10\text{-}13}(s,b,a)$ 554. (*SPI*)
566 | | | | $R_{11\text{-}7}(s,a,b) \land \neg R_{11\text{-}7}(s,b,a)$ 554. (*SPI*)
567 | | | | $R_{11\text{-}8}(s,a,b) \land \neg R_{11\text{-}8}(s,b,a)$ 554. (*SPI*)
568 | | | | $R_{11\text{-}13}(s,a,b) \land \neg R_{11\text{-}13}(s,b,a)$ 554. (*SPI*)
569 | | | | $R_{3\text{-}9}(s,b,c) \land \neg R_{3\text{-}9}(s,c,b)$ 246, 503, (*SPT*)
570 | | | | $R_{1\text{-}9}(s,b,c) \land \neg R_{1\text{-}9}(s,c,b)$ 569, (*SPI*)
571 | | | | $R_{1\text{-}10}(s,b,c) \land \neg R_{1\text{-}10}(s,c,b)$ 569, (*SPI*)
572 | | | | $R_{1\text{-}13}(s,b,c) \land \neg R_{1\text{-}13}(s,c,b)$ 569, (*SPI*)
573 | | | | $R_{3\text{-}10}(s,b,c) \land \neg R_{3\text{-}10}(s,c,b)$ 569, (*SPI*)
574 | | | | $R_{3\text{-}13}(s,b,c) \land \neg R_{3\text{-}13}(s,c,b)$ 569, (*SPI*)
575 | | | | $R_{4\text{-}9}(s,b,c) \land \neg R_{4\text{-}9}(s,c,b)$ 569, (*SPI*)
576 | | | | $R_{4\text{-}10}(s,b,c) \land \neg R_{4\text{-}10}(s,c,b)$ 569, (*SPI*)
577 | | | | $R_{4\text{-}13}(s,b,c) \land \neg R_{4\text{-}13}(s,c,b)$ 569, (*SPI*)
578 | | | | $R_{7\text{-}9}(s,b,c) \land \neg R_{7\text{-}9}(s,c,b)$ 569, (*SPI*)
579 | | | | $R_{7\text{-}10}(s,b,c) \land \neg R_{7\text{-}10}(s,c,b)$ 569, (*SPI*)
580 | | | | $R_{7\text{-}13}(s,b,c) \land \neg R_{7\text{-}13}(s,c,b)$ 569, (*SPI*)
581 | | | | $R_{12\text{-}9}(s,b,c) \land \neg R_{12\text{-}9}(s,c,b)$ 569, (*SPI*)
582 | | | | $R_{12\text{-}10}(s,b,c) \land \neg R_{12\text{-}10}(s,c,b)$ 569, (*SPI*)
583 | | | | $R_{12\text{-}13}(s,b,c) \land \neg R_{12\text{-}13}(s,c,b)$ 569, (*SPI*)
584 | | | | $\neg R_{4\text{-}5}(s,a,b) \land R_{4\text{-}5}(s,b,a)$ 256, 475, (*SPT*)
585 | | | | $\neg R_{3\text{-}1}(s,a,b) \land R_{3\text{-}1}(s,b,a)$ 584, (*SPI*)
586 | | | | $\neg R_{3\text{-}2}(s,a,b) \land R_{3\text{-}2}(s,b,a)$ 584, (*SPI*)
587 | | | | $\neg R_{3\text{-}5}(s,a,b) \land R_{3\text{-}5}(s,b,a)$ 584, (*SPI*)
588 | | | | $\neg R_{3\text{-}10}(s,a,b) \land R_{3\text{-}10}(s,b,a)$ 584, (*SPI*)
589 | | | | $\neg R_{3\text{-}11}(s,a,b) \land R_{3\text{-}11}(s,b,a)$ 584, (*SPI*)
590 | | | | $\neg R_{4\text{-}1}(s,a,b) \land R_{4\text{-}1}(s,b,a)$ 584, (*SPI*)
591 | | | | $\neg R_{4\text{-}2}(s,a,b) \land R_{4\text{-}2}(s,b,a)$ 584, (*SPI*)
592 | | | | $\neg R_{4\text{-}10}(s,a,b) \land R_{4\text{-}10}(s,b,a)$ 584, (*SPI*)
593 | | | | $\neg R_{4\text{-}11}(s,a,b) \land R_{4\text{-}11}(s,b,a)$ 584, (*SPI*)
594 | | | | $\neg R_{6\text{-}1}(s,a,b) \land R_{6\text{-}1}(s,b,a)$ 584, (*SPI*)

595 | | | | $\neg R_{6\text{-}2}(s,a,b) \land R_{6\text{-}2}(s,b,a)$ 584, (*SPI*)
596 | | | | $\neg R_{6\text{-}5}(s,a,b) \land R_{6\text{-}5}(s,b,a)$ 584, (*SPI*)
597 | | | | $\neg R_{6\text{-}10}(s,a,b) \land R_{6\text{-}10}(s,b,a)$ 584, (*SPI*)
598 | | | | $\neg R_{6\text{-}11}(s,a,b) \land R_{6\text{-}11}(s,b,a)$ 584, (*SPI*)
599 | | | | $\neg R_{9\text{-}1}(s,a,b) \land R_{9\text{-}1}(s,b,a)$ 584, (*SPI*)
600 | | | | $\neg R_{9\text{-}2}(s,a,b) \land R_{9\text{-}2}(s,b,a)$ 584, (*SPI*)
601 | | | | $\neg R_{9\text{-}5}(s,a,b) \land R_{9\text{-}5}(s,b,a)$ 584, (*SPI*)
602 | | | | $\neg R_{9\text{-}10}(s,a,b) \land R_{9\text{-}10}(s,b,a)$ 584, (*SPI*)
603 | | | | $\neg R_{9\text{-}11}(s,a,b) \land R_{9\text{-}11}(s,b,a)$ 584, (*SPI*)
604 | | | | $\neg R_{12\text{-}1}(s,a,b) \land R_{12\text{-}1}(s,b,a)$ 584, (*SPI*)
605 | | | | $\neg R_{12\text{-}2}(s,a,b) \land R_{12\text{-}2}(s,b,a)$ 584, (*SPI*)
606 | | | | $\neg R_{12\text{-}5}(s,a,b) \land R_{12\text{-}5}(s,b,a)$ 584, (*SPI*)
607 | | | | $\neg R_{12\text{-}10}(s,a,b) \land R_{12\text{-}10}(s,b,a)$ 584, (*SPI*)
608 | | | | $\neg R_{12\text{-}11}(s,a,b) \land R_{12\text{-}11}(s,b,a)$ 584, (*SPI*)
609 | | | | $\neg R_{4\text{-}8}(s,a,b) \land R_{4\text{-}8}(s,b,a)$ 260, 477, (*SPT*)
610 | | | | $\neg R_{3\text{-}7}(s,a,b) \land R_{3\text{-}7}(s,b,a)$ 609, (*SPI*)
611 | | | | $\neg R_{3\text{-}8}(s,a,b) \land R_{3\text{-}8}(s,b,a)$ 609, (*SPI*)
612 | | | | $\neg R_{3\text{-}13}(s,a,b) \land R_{3\text{-}13}(s,b,a)$ 609, (*SPI*)
613 | | | | $\neg R_{4\text{-}7}(s,a,b) \land R_{4\text{-}7}(s,b,a)$ 609, (*SPI*)
614 | | | | $\neg R_{4\text{-}13}(s,a,b) \land R_{4\text{-}13}(s,b,a)$ 609, (*SPI*)
615 | | | | $\neg R_{6\text{-}7}(s,a,b) \land R_{6\text{-}7}(s,b,a)$ 609, (*SPI*)
616 | | | | $\neg R_{6\text{-}8}(s,a,b) \land R_{6\text{-}8}(s,b,a)$ 609, (*SPI*)
617 | | | | $\neg R_{6\text{-}13}(s,a,b) \land R_{6\text{-}13}(s,b,a)$ 609, (*SPI*)
618 | | | | $\neg R_{9\text{-}7}(s,a,b) \land R_{9\text{-}7}(s,b,a)$ 609, (*SPI*)
619 | | | | $\neg R_{9\text{-}8}(s,a,b) \land R_{9\text{-}8}(s,b,a)$ 609, (*SPI*)
620 | | | | $\neg R_{9\text{-}13}(s,a,b) \land R_{9\text{-}13}(s,b,a)$ 609, (*SPI*)
621 | | | | $\neg R_{12\text{-}7}(s,a,b) \land R_{12\text{-}7}(s,b,a)$ 609, (*SPI*)
622 | | | | $\neg R_{12\text{-}8}(s,a,b) \land R_{12\text{-}8}(s,b,a)$ 609, (*SPI*)
623 | | | | $\neg R_{12\text{-}13}(s,a,b) \land R_{12\text{-}13}(s,b,a)$ 609, (*SPI*)
624 | | | | $\neg R_{5\text{-}4}(s,b,c) \land R_{5\text{-}4}(s,c,b)$ 268, 525, (*SPT*)
625 | | | | $\neg R_{2\text{-}1}(s,b,c) \land R_{2\text{-}1}(s,c,b)$ 624, (*SPI*)
626 | | | | $\neg R_{2\text{-}3}(s,b,c) \land R_{2\text{-}3}(s,c,b)$ 624, (*SPI*)
627 | | | | $\neg R_{2\text{-}4}(s,b,c) \land R_{2\text{-}4}(s,c,b)$ 624, (*SPI*)
628 | | | | $\neg R_{2\text{-}7}(s,b,c) \land R_{2\text{-}7}(s,c,b)$ 624, (*SPI*)
629 | | | | $\neg R_{2\text{-}12}(s,b,c) \land R_{2\text{-}12}(s,c,b)$ 624, (*SPI*)
630 | | | | $\neg R_{5\text{-}1}(s,b,c) \land R_{5\text{-}1}(s,c,b)$ 624, (*SPI*)
631 | | | | $\neg R_{5\text{-}3}(s,b,c) \land R_{5\text{-}3}(s,c,b)$ 624, (*SPI*)
632 | | | | $\neg R_{5\text{-}7}(s,b,c) \land R_{5\text{-}7}(s,c,b)$ 624, (*SPI*)
633 | | | | $\neg R_{5\text{-}12}(s,b,c) \land R_{5\text{-}12}(s,c,b)$ 624, (*SPI*)
634 | | | | $\neg R_{6\text{-}1}(s,b,c) \land R_{6\text{-}1}(s,c,b)$ 624, (*SPI*)
635 | | | | $\neg R_{6\text{-}3}(s,b,c) \land R_{6\text{-}3}(s,c,b)$ 624, (*SPI*)
636 | | | | $\neg R_{6\text{-}4}(s,b,c) \land R_{6\text{-}4}(s,c,b)$ 624, (*SPI*)
637 | | | | $\neg R_{6\text{-}7}(s,b,c) \land R_{6\text{-}7}(s,c,b)$ 624, (*SPI*)
638 | | | | $\neg R_{6\text{-}12}(s,b,c) \land R_{6\text{-}12}(s,c,b)$ 624, (*SPI*)
639 | | | | $\neg R_{8\text{-}1}(s,b,c) \land R_{8\text{-}1}(s,c,b)$ 624, (*SPI*)
640 | | | | $\neg R_{8\text{-}3}(s,b,c) \land R_{8\text{-}3}(s,c,b)$ 624, (*SPI*)
641 | | | | $\neg R_{8\text{-}4}(s,b,c) \land R_{8\text{-}4}(s,c,b)$ 624, (*SPI*)
642 | | | | $\neg R_{8\text{-}7}(s,b,c) \land R_{8\text{-}7}(s,c,b)$ 624, (*SPI*)
643 | | | | $\neg R_{8\text{-}12}(s,b,c) \land R_{8\text{-}12}(s,c,b)$ 624, (*SPI*)
644 | | | | $\neg R_{11\text{-}1}(s,b,c) \land R_{11\text{-}1}(s,c,b)$ 624, (*SPI*)
645 | | | | $\neg R_{11\text{-}3}(s,b,c) \land R_{11\text{-}3}(s,c,b)$ 624, (*SPI*)
646 | | | | $\neg R_{11\text{-}4}(s,b,c) \land R_{11\text{-}4}(s,c,b)$ 624, (*SPI*)
647 | | | | $\neg R_{11\text{-}7}(s,b,c) \land R_{11\text{-}7}(s,c,b)$ 624, (*SPI*)
648 | | | | $\neg R_{11\text{-}12}(s,b,c) \land R_{11\text{-}12}(s,c,b)$ 624, (*SPI*)
649 | | | | $\neg R_{5\text{-}9}(s,b,c) \land R_{5\text{-}9}(s,c,b)$ 276, 527, (*SPT*)
650 | | | | $\neg R_{2\text{-}9}(s,b,c) \land R_{2\text{-}9}(s,c,b)$ 649, (*SPI*)
651 | | | | $\neg R_{2\text{-}10}(s,b,c) \land R_{2\text{-}10}(s,c,b)$ 649, (*SPI*)
652 | | | | $\neg R_{2\text{-}13}(s,b,c) \land R_{2\text{-}13}(s,c,b)$ 649, (*SPI*)
653 | | | | $\neg R_{5\text{-}10}(s,b,c) \land R_{5\text{-}10}(s,c,b)$ 649, (*SPI*)
654 | | | | $\neg R_{5\text{-}13}(s,b,c) \land R_{5\text{-}13}(s,c,b)$ 649, (*SPI*)
655 | | | | $\neg R_{6\text{-}9}(s,b,c) \land R_{6\text{-}9}(s,c,b)$ 649, (*SPI*)
656 | | | | $\neg R_{6\text{-}10}(s,b,c) \land R_{6\text{-}10}(s,c,b)$ 649, (*SPI*)
657 | | | | $\neg R_{6\text{-}13}(s,b,c) \land R_{6\text{-}13}(s,c,b)$ 649, (*SPI*)
658 | | | | $\neg R_{8\text{-}9}(s,b,c) \land R_{8\text{-}9}(s,c,b)$ 649, (*SPI*)
659 | | | | $\neg R_{8\text{-}10}(s,b,c) \land R_{8\text{-}10}(s,c,b)$ 649, (*SPI*)
660 | | | | $\neg R_{8\text{-}13}(s,b,c) \land R_{8\text{-}13}(s,c,b)$ 649, (*SPI*)
661 | | | | $\neg R_{11\text{-}9}(s,b,c) \land R_{11\text{-}9}(s,c,b)$ 649, (*SPI*)
662 | | | | $\neg R_{11\text{-}10}(s,b,c) \land R_{11\text{-}10}(s,c,b)$ 649, (*SPI*)
663 | | | | $\neg R_{11\text{-}13}(s,b,c) \land R_{11\text{-}13}(s,c,b)$ 649, (*SPI*)
664 | | | | $\neg R_{6\text{-}1}(s,a,c) \land R_{6\text{-}1}(s,c,a)$ 594, 634, (*SPT*)
665 | | | | $\neg R_{4\text{-}1}(s,a,c) \land R_{4\text{-}1}(s,c,a)$ 664, (*SPI*)
666 | | | | $\neg R_{4\text{-}2}(s,a,c) \land R_{4\text{-}2}(s,c,a)$ 664, (*SPI*)
667 | | | | $\neg R_{4\text{-}3}(s,a,c) \land R_{4\text{-}3}(s,c,a)$ 664, (*SPI*)
668 | | | | $\neg R_{4\text{-}7}(s,a,c) \land R_{4\text{-}7}(s,c,a)$ 664, (*SPI*)
669 | | | | $\neg R_{4\text{-}10}(s,a,c) \land R_{4\text{-}10}(s,c,a)$ 664, (*SPI*)
670 | | | | $\neg R_{5\text{-}1}(s,a,c) \land R_{5\text{-}1}(s,c,a)$ 664, (*SPI*)
671 | | | | $\neg R_{5\text{-}2}(s,a,c) \land R_{5\text{-}2}(s,c,a)$ 664, (*SPI*)
672 | | | | $\neg R_{5\text{-}3}(s,a,c) \land R_{5\text{-}3}(s,c,a)$ 664, (*SPI*)
673 | | | | $\neg R_{5\text{-}7}(s,a,c) \land R_{5\text{-}7}(s,c,a)$ 664, (*SPI*)
674 | | | | $\neg R_{5\text{-}10}(s,a,c) \land R_{5\text{-}10}(s,c,a)$ 664, (*SPI*)
675 | | | | $\neg R_{6\text{-}2}(s,a,c) \land R_{6\text{-}2}(s,c,a)$ 664, (*SPI*)
676 | | | | $\neg R_{6\text{-}3}(s,a,c) \land R_{6\text{-}3}(s,c,a)$ 664, (*SPI*)
677 | | | | $\neg R_{6\text{-}7}(s,a,c) \land R_{6\text{-}7}(s,c,a)$ 664, (*SPI*)
678 | | | | $\neg R_{6\text{-}10}(s,a,c) \land R_{6\text{-}10}(s,c,a)$ 664, (*SPI*)
679 | | | | $\neg R_{8\text{-}1}(s,a,c) \land R_{8\text{-}1}(s,c,a)$ 664, (*SPI*)
680 | | | | $\neg R_{8\text{-}2}(s,a,c) \land R_{8\text{-}2}(s,c,a)$ 664, (*SPI*)
681 | | | | $\neg R_{8\text{-}3}(s,a,c) \land R_{8\text{-}3}(s,c,a)$ 664, (*SPI*)
682 | | | | $\neg R_{8\text{-}7}(s,a,c) \land R_{8\text{-}7}(s,c,a)$ 664, (*SPI*)
683 | | | | $\neg R_{8\text{-}10}(s,a,c) \land R_{8\text{-}10}(s,c,a)$ 664, (*SPI*)
684 | | | | $\neg R_{9\text{-}1}(s,a,c) \land R_{9\text{-}1}(s,c,a)$ 664, (*SPI*)
685 | | | | $\neg R_{9\text{-}2}(s,a,c) \land R_{9\text{-}2}(s,c,a)$ 664, (*SPI*)
686 | | | | $\neg R_{9\text{-}3}(s,a,c) \land R_{9\text{-}3}(s,c,a)$ 664, (*SPI*)
687 | | | | $\neg R_{9\text{-}7}(s,a,c) \land R_{9\text{-}7}(s,c,a)$ 664, (*SPI*)
688 | | | | $\neg R_{9\text{-}10}(s,a,c) \land R_{9\text{-}10}(s,c,a)$ 664, (*SPI*)
689 | | | | $\neg R_{6\text{-}11}(s,a,c) \land R_{6\text{-}11}(s,c,a)$ 293, 617, (*SPT*)
690 | | | | $\neg R_{4\text{-}11}(s,a,c) \land R_{4\text{-}11}(s,c,a)$ 689, (*SPI*)
691 | | | | $\neg R_{4\text{-}12}(s,a,c) \land R_{4\text{-}12}(s,c,a)$ 689, (*SPI*)
692 | | | | $\neg R_{4\text{-}13}(s,a,c) \land R_{4\text{-}13}(s,c,a)$ 689, (*SPI*)
693 | | | | $\neg R_{5\text{-}11}(s,a,c) \land R_{5\text{-}11}(s,c,a)$ 689, (*SPI*)
694 | | | | $\neg R_{5\text{-}12}(s,a,c) \land R_{5\text{-}12}(s,c,a)$ 689, (*SPI*)
695 | | | | $\neg R_{5\text{-}13}(s,a,c) \land R_{5\text{-}13}(s,c,a)$ 689, (*SPI*)
696 | | | | $\neg R_{6\text{-}12}(s,a,c) \land R_{6\text{-}12}(s,c,a)$ 689, (*SPI*)
697 | | | | $\neg R_{6\text{-}13}(s,a,c) \land R_{6\text{-}13}(s,c,a)$ 689, (*SPI*)
698 | | | | $\neg R_{8\text{-}11}(s,a,c) \land R_{8\text{-}11}(s,c,a)$ 689, (*SPI*)
699 | | | | $\neg R_{8\text{-}12}(s,a,c) \land R_{8\text{-}12}(s,c,a)$ 689, (*SPI*)
700 | | | | $\neg R_{8\text{-}13}(s,a,c) \land R_{8\text{-}13}(s,c,a)$ 689, (*SPI*)
701 | | | | $\neg R_{9\text{-}11}(s,a,c) \land R_{9\text{-}11}(s,c,a)$ 689, (*SPI*)
702 | | | | $\neg R_{9\text{-}12}(s,a,c) \land R_{9\text{-}12}(s,c,a)$ 689, (*SPI*)
703 | | | | $\neg R_{9\text{-}13}(s,a,c) \land R_{9\text{-}13}(s,c,a)$ 689, (*SPI*)
704 | | | | $\forall w \neg(H(w) \land \forall X(\boldsymbol{P}(X) \to \forall x \forall y((A(x) \land A(y)) \to ((X(w,x,y) \land \neg X(w,y,x)) \to (X(s,x,y) \land \neg X(s,y,x))))) \land \forall u(H(u) \to (\forall X(\boldsymbol{P}(X) \to \forall x \forall y((A(x) \land A(y)) \to ((X(u,x,y) \land \neg X(u,y,x)) \to (X(s,x,y) \land \neg X(s,y,x))))) \to u = w)))$ 178, (rep.)
705 | | | | $\neg(H(p) \land \forall X(\boldsymbol{P}(X) \to \forall x \forall y((A(x) \land A(y)) \to ((X(p,x,y) \land \neg X(p,y,x)) \to (X(s,x,y) \land \neg X(s,y,x))))) \land \forall u(H(u) \to (\forall X(\boldsymbol{P}(X) \to \forall x \forall y((A(x) \land A(y)) \to ((X(u,x,y) \land \neg X(u,y,x)) \to (X(s,x,y) \land \neg X(s,y,x))))) \to u = p)))$ 704, ($\forall E$)
706 | | | | $\neg H(p) \lor \neg \forall X(\boldsymbol{P}(X) \to \forall x \forall y((A(x) \land A(y)) \to ((X(p,x,y) \land \neg X(p,y,x)) \to (X(s,x,y) \land \neg X(s,y,x))))) \lor \neg \forall u(H(u) \to (\forall X(\boldsymbol{P}(X) \to \forall x \forall y((A(x) \land A(y)) \to ((X(u,x,y) \land \neg X(u,y,x)) \to (X(s,x,y) \land \neg X(s,y,x))))) \to u = p))$ 705, (rep.)
707 | | | | | $\neg H(p)$ prem.
708 | | | | | $H(p)$ 1, ($\land E$)
709 | | | | | $\bot$ 707, 708, ($\neg E$)
710 | | | | $\neg H(p) \to \bot$ 707, 709, ($\to I$)
711 | | | | | $\neg \forall X(\boldsymbol{P}(X) \to \forall x \forall y((A(x) \land A(y)) \to ((X(p,x,y) \land \neg X(p,y,x)) \to (X(s,x,y) \land \neg X(s,y,x)))))$ prem.
712 | | | | | | $\boldsymbol{P}(R_0)$ prem.
713 | | | | | | | $\neg \forall x \forall y((A(x) \land A(y)) \to ((R_0(p,x,y) \land \neg R_0(p,y,x)) \to (R_0(s,x,y) \land \neg R_0(s,y,x))))$ prem.
714 | | | | | | | $\forall X(\boldsymbol{P}(X) \to (X = R_{1\text{-}1} \lor \ldots \lor X = R_{13\text{-}13}))$ 3, ($\land E$)
715 | | | | | | | $\boldsymbol{P}(R_0) \to (R_0 = R_{1\text{-}1} \lor \ldots \lor R_0 = R_{13\text{-}13})$ 714, ($\forall E$)
716 | | | | | | | $R_0 = R_{1\text{-}1} \lor \ldots \lor R_0 = R_{13\text{-}13}$ 712, 715, ($\to E$)
717 | | | | | | | | $R_0 = R_{1\text{-}1}$ prem.
718 | | | | | | | | $\exists x \exists y \neg((A(x) \land A(y)) \to ((R_0(p,x,y) \land \neg R_0(p,y,x)) \to (R_0(s,x,y) \land \neg R_0(s,y,x))))$ 713, (rep.)
719 | | | | | | | | | $\neg((A(d) \land A(e)) \to ((R_0(p,d,e) \land \neg R_0(p,e,d)) \to (R_0(s,d,e) \land \neg R_0(s,e,d))))$ prem.
720 | | | | | | | | | $\neg((A(d) \land A(e)) \to ((R_{1\text{-}1}(p,d,e) \land \neg R_{1\text{-}1}(p,e,d)) \to (R_{1\text{-}1}(s,d,e) \land \neg R_{1\text{-}1}(s,e,d))))$ 717, 719, ($=E$)
721 | | | | | | | | | | $(A(d) \land A(e)$ prem.
722 | | | | | | | | | | | $R_{1\text{-}1}(p,d,e) \land \neg R_{1\text{-}1}(p,e,d)$ prem.
723 | | | | | | | | | | | | $\neg(R_{1\text{-}1}(s,d,e) \land \neg R_{1\text{-}1}(s,e,d))$ prem.
724 | | | | | | | | | | | | $\forall x(A(x) \to (x = a \lor x = b \lor x = c))$ 2, ($\land E$)
725 | | | | | | | | | | | | $A(d) \to (d = a \lor d = b \lor d = c)$ 724, ($\forall E$)
726 | | | | | | | | | | | | $A(e) \to (e = a \lor e = b \lor e = c)$ 724, ($\forall E$)
727 | | | | | | | | | | | | $A(d)$ 721, ($\land E$)
728 | | | | | | | | | | | | $d = a \lor d = b \lor d = c$ 725, 727, ($\to E$)
729 | | | | | | | | | | | | $A(e)$ 721, ($\land E$)
730 | | | | | | | | | | | | $e = a \lor e = b \lor e = c$ 726, 729, ($\land E$), ($\to E$)
731 | | | | | | | | | | | | | $d = a$ prem.
732 | | | | | | | | | | | | | $e = a \lor e = b \lor e = c$ 730, (rep.)
733 | | | | | | | | | | | | | | $e = a$ prem.
734 | | | | | | | | | | | | | | $R_{1\text{-}1}(p,a,a) \land \neg R_{1\text{-}1}(p,a,a)$ 722, 731, 733, ($=E$)
735 | | | | | | | | | | | | | | $\bot$ 734, ($\neg E$)
736 | | | | | | | | | | | | | $(e = a) \to \bot$ 733, 735, ($\to I$)
737 | | | | | | | | | | | | | | $e = b$ prem.
738 | | | | | | | | | | | | | | $\neg(R_{1\text{-}1}(s,a,b) \land \neg R_{1\text{-}1}(s,b,a))$ 723, 731, 737, ($=E$)
739 | | | | | | | | | | | | | | $R_{1\text{-}1}(s,a,b) \land \neg R_{1\text{-}1}(s,b,a)$ 205, (rep.)
740 | | | | | | | | | | | | | | $\bot$ 738, 739, ($\neg E$)
741 | | | | | | | | | | | | | $(e = b) \to \bot$ 737, 740, ($\to I$)
742 | | | | | | | | | | | | | | $e = c$ prem.
743 | | | | | | | | | | | | | | $\neg(R_{1\text{-}1}(s,a,c) \land \neg R_{1\text{-}1}(s,c,a))$ 723, 731, 742, ($=E$)
744 | | | | | | | | | | | | | | $R_{1\text{-}1}(s,a,c) \land \neg R_{1\text{-}1}(s,c,a)$ 207, (rep.)
745 | | | | | | | | | | | | | | $\bot$ 743, 744, ($\neg E$)

746 $(e = c) \to \bot$ 742, 745
747 $\bot$ 732, 736, 741, 746, $(\vee E)$
748 $(d = a) \to \bot$ 731, 747, $(\to I)$
749 $d = b$ prem.
750 $e = a \vee e = b \vee e = c$ 730, (rep.)
751 $e = a$ prem.
752 $R_{1\text{-}1}(p, d, e)$ 722, $(\wedge E)$
753 $R_{1\text{-}1}(p, b, a)$ 749, 751, 752, $(=E)$
754 $\neg R_{1\text{-}1}(p, b, a)$ 4, $(\wedge E)$
755 $\bot$ 753, 754, $(\neg E)$
756 $(e = a) \to \bot$ 751, 755, $(\to I)$
757 $e = b$ prem.
758 $R_{1\text{-}1}(p, b, b) \wedge \neg R_{1\text{-}1}(p, b, b)$ 722, 749, 757, $(=E)$
759 $\bot$ 758, $(\neg E)$
760 $(e = b) \to \bot$ 757, 759, $(\to I)$
761 $e = c$ prem.
762 $\neg(R_{1\text{-}1}(s, b, c) \wedge \neg R_{1\text{-}1}(s, c, b))$ 723, 749, 761, $(=E)$
763 $R_{1\text{-}1}(s, b, c) \wedge \neg R_{1\text{-}1}(s, c, b)$ 206, (rep.)
764 $\bot$ 762, 763, $(\neg E)$
765 $(e = c) \to \bot$ 761, 764, $(\to I)$
766 $\bot$ 750, 756, 760, 765, $(\vee E)$
767 $(d = b) \to \bot$ 749, 766, $(\to I)$
768 $d = c$ prem.
769 $e = a \vee e = b \vee e = c$ 730, (rep.)
770 $e = a$ prem.
771 $R_{1\text{-}1}(p, d, e)$ 722, $(\wedge E)$
772 $R_{1\text{-}1}(p, c, a)$ 768, 770, 771, $(=E)$
773 $\neg R_{1\text{-}1}(p, c, a)$ 4, $(\wedge E)$
774 $\bot$ 772, 773, $(\neg E)$
775 $(e = a) \to \bot$ 770, 774, $(\to I)$
776 $e = b$ prem.
777 $R_{1\text{-}1}(p, d, e)$ 722, $(\wedge E)$
778 $R_{1\text{-}1}(p, c, b)$ 768, 776, 777, $(=E)$
779 $\neg R_{1\text{-}1}(p, c, b)$ 4, $(\wedge E)$
780 $\bot$ 778, 779, $(\neg E)$
781 $(e = b) \to \bot$ 776, 780, $(\to I)$
782 $e = c$ prem.
783 $R_{1\text{-}1}(p, c, c) \wedge \neg R_{1\text{-}1}(p, c, c)$ 722, 768, 782, $(=E)$
784 $\bot$ 783, $(\neg E)$
785 $(e = c) \to \bot$ 782, 784, $(\to I)$
786 $\bot$ 769, 775, 781, 785, $(\vee E)$
787 $(d = c) \to \bot$ 768, 786, $(\to I)$
788 $\bot$ 728, 748, 767, 787, $(\vee E)$
789 $R_{1\text{-}1}(s, d, e) \wedge \neg R_{1\text{-}1}(s, e, d)$ 723, 788, $(\neg I)$
790 $(R_{1\text{-}1}(p, d, e) \wedge \neg R_{1\text{-}1}(p, e, d)) \to (R_{1\text{-}1}(s, d, e) \wedge \neg R_{1\text{-}1}(s, e, d))$ 722, 789, (DNE)
791 $(A(d) \wedge A(e)) \to ((R_{1\text{-}1}(p, d, e) \wedge \neg R_{1\text{-}1}(p, e, d)) \to (R_{1\text{-}1}(s, d, e) \wedge \neg R_{1\text{-}1}(s, e, d)))$ 721, 790, $(\to I)$
792 $\bot$ 720, 791, $(\neg E)$
793 $\bot$ 718, 792, $(\exists E)$
794 $(R_0 = R_{1\text{-}1}) \to \bot$ 717, 793, $(\to I)$
795 $R_0 = R_{1\text{-}2}$ prem.
796 $\bot$ 5, 208, 209, 362, 464, (similar procedure examining a whole profile I 718–793 [*SPW I*])
797 $(R_0 = R_{1\text{-}2}) \to \bot$ 795, 796, $(\to I)$
798 $R_0 = R_{1\text{-}3}$ prem.
799 $\bot$ 6, 210, 211, 515, (*SPW I*)
800 $(R_0 = R_{1\text{-}3}) \to \bot$ 798, 799, $(\to I)$
801 $R_0 = R_{1\text{-}4}$ prem.
802 $\bot$ 7, 212, 490, 516, (*SPW I*)
803 $(R_0 = R_{1\text{-}4}) \to \bot$ 801, 802, $(\to I)$
804 $R_0 = R_{1\text{-}5}$ prem.
805 $\bot$ 8, 213, 465, 489, (*SPW I*)
806 $(R_0 = R_{1\text{-}5}) \to \bot$ 804, 805, $(\to I)$
807 $R_0 = R_{1\text{-}6}$ prem.
808 $\bot$ 9, 466, 491, 517, (*SPW I*)
809 $(R_0 = R_{1\text{-}6}) \to \bot$ 807, 808, $(\to I)$
810 $R_0 = R_{1\text{-}7}$ prem.
811 $\bot$ 10, 214, 215, 555, (*SPW I*)
812 $(R_0 = R_{1\text{-}7}) \to \bot$ 810, 811, $(\to I)$
813 $R_0 = R_{1\text{-}8}$ prem.
814 $\bot$ 11, 467, 492, 556, (*SPW I*)
815 $(R_0 = R_{1\text{-}8}) \to \bot$ 813, 814, $(\to I)$
816 $R_0 = R_{1\text{-}9}$ prem.
817 $\bot$ 12, 493, 518, 570, (*SPW I*)
818 $(R_0 = R_{1\text{-}9}) \to \bot$ 816, 817, $(\to I)$
819 $R_0 = R_{1\text{-}10}$ prem.
820 $\bot$ 13, 216, 217, 571, (*SPW I*)
821 $(R_0 = R_{1\text{-}10}) \to \bot$ 819, 820, $(\to I)$
822 $R_0 = R_{1\text{-}11}$ prem.
823 $\bot$ 14, 218, 468, 539, (*SPW I*)
824 $(R_0 = R_{1\text{-}11}) \to \bot$ 822, 823, $(\to I)$
825 $R_0 = R_{1\text{-}12}$ prem.
826 $\bot$ 15, 219, 519, 540, (*SPW I*)
827 $(R_0 = R_{1\text{-}12}) \to \bot$ 825, 826, $(\to I)$
828 $R_0 = R_{1\text{-}13}$ prem.
829 $\bot$ 16, 541, 557, 572, (*SPW I*)
830 $(R_0 = R_{1\text{-}13}) \to \bot$ 828, 829, $(\to I)$
831 $R_0 = R_{2\text{-}1}$ prem.
832 $\bot$ 17, 220, 221, 625, (*SPW I*)
833 $(R_0 = R_{2\text{-}1}) \to \bot$ 831, 832, $(\to I)$
834 $R_0 = R_{2\text{-}2}$ prem.
835 $\bot$ 18, 222–224, (*SPW I*)
836 $(R_0 = R_{2\text{-}2}) \to \bot$ 834, 845, $(\to I)$
837 $R_0 = R_{2\text{-}3}$ prem.
838 $\bot$ 19, 225, 520, 626, (*SPW I*)
839 $(R_0 = R_{2\text{-}3}) \to \bot$ 837, 838, $(\to I)$
840 $R_0 = R_{2\text{-}4}$ prem.
841 $\bot$ 20, 494, 521, 627, (*SPW I*)
842 $(R_0 = R_{2\text{-}4}) \to \bot$ 840, 841, $(\to I)$
843 $R_0 = R_{2\text{-}5}$ prem.
844 $\bot$ 21, 226, 227, 495, (*SPW I*)
845 $(R_0 = R_{2\text{-}5}) \to \bot$ 843, 844, $(\to I)$
846 $R_0 = R_{2\text{-}6}$ prem.
847 $\bot$ 22, 228, 496, 514, (*SPW I*)
848 $(R_0 = R_{2\text{-}6}) \to \bot$ 846, 847, $(\to I)$
849 $R_0 = R_{2\text{-}7}$ prem.
850 $\bot$ 23, 229, 558, 628, (*SPW I*)
851 $(R_0 = R_{2\text{-}7}) \to \bot$ 849, 850, $(\to I)$
852 $R_0 = R_{2\text{-}8}$ prem.
853 $\bot$ 24, 230, 497, 554, (*SPW I*)
854 $(R_0 = R_{2\text{-}8}) \to \bot$ 852, 853, $(\to I)$
855 $R_0 = R_{2\text{-}9}$ prem.
856 $\bot$ 25, 498, 522, 650, (*SPW I*)
857 $(R_0 = R_{2\text{-}9}) \to \bot$ 855, 856, $(\to I)$
858 $R_0 = R_{2\text{-}10}$ prem.
859 $\bot$ 26, 231, 232, 651, (*SPW I*)
860 $(R_0 = R_{2\text{-}10}) \to \bot$ 858, 859, $(\to I)$
861 $R_0 = R_{2\text{-}11}$ prem.
862 $\bot$ 27, 233, 234, 542, (*SPW I*)
863 $(R_0 = R_{2\text{-}11}) \to \bot$ 861, 862, $(\to I)$
864 $R_0 = R_{2\text{-}12}$ prem.
865 $\bot$ 28, 523, 543, 629, (*SPW I*)
866 $(R_0 = R_{2\text{-}12}) \to \bot$ 864, 865, $(\to I)$
867 $R_0 = R_{2\text{-}13}$ prem.
868 $\bot$ 29, 559, 652, 544, (*SPW I*)
869 $(R_0 = R_{2\text{-}13}) \to \bot$ 867, 868, $(\to I)$
870 $R_0 = R_{3\text{-}1}$ prem.
871 $\bot$ 30, 235, 236, 585, (*SPW I*)
872 $(R_0 = R_{3\text{-}1}) \to \bot$ 870, 871, $(\to I)$
873 $R_0 = R_{3\text{-}2}$ prem.
874 $\bot$ 31, 237, 469, 586, (*SPW I*)
875 $(R_0 = R_{3\text{-}2}) \to \bot$ 873, 874, $(\to I)$
876 $R_0 = R_{3\text{-}3}$ prem.
877 $\bot$ 32, 238–240, (*SPW I*)
878 $(R_0 = R_{3\text{-}3}) \to \bot$ 876, 877, $(\to I)$
879 $R_0 = R_{3\text{-}4}$ prem.
880 $\bot$ 33, 241, 242, 499, (*SPW I*)
881 $(R_0 = R_{3\text{-}4}) \to \bot$ 879, 880, $(\to I)$
882 $R_0 = R_{3\text{-}5}$ prem.
883 $\bot$ 34, 470, 500, 587, (*SPW I*)
884 $(R_0 = R_{3\text{-}5}) \to \bot$ 882, 883, $(\to I)$
885 $R_0 = R_{3\text{-}6}$ prem.
886 $\bot$ 35, 243, 471, 501, (*SPW I*)
887 $(R_0 = R_{3\text{-}6}) \to \bot$ 885, 886, $(\to I)$
888 $R_0 = R_{3\text{-}7}$ prem.
889 $\bot$ 36, 244, 245, 610, (*SPW I*)
890 $(R_0 = R_{3\text{-}7}) \to \bot$ 888, 889, $(\to I)$
891 $R_0 = R_{3\text{-}8}$ prem.
892 $\bot$ 37, 472, 502, 611, (*SPW I*)
893 $(R_0 = R_{3\text{-}8}) \to \bot$ 891, 892, $(\to I)$
894 $R_0 = R_{3\text{-}9}$ prem.
895 $\bot$ 38, 246, 503, 569, (*SPW I*)
896 $(R_0 = R_{3\text{-}9}) \to \bot$ 894, 895, $(\to I)$

897 | | | | | | | | $R_0 = R_{3\text{-}10}$ prem.
898 | | | | | | | | ⊥ 39, 247, 573, 588, (*SPW I*)
899 | | | | | | | $(R_0 = R_{3\text{-}10}) \to \bot$ 897, 898, ($\to I$)
900 | | | | | | | | $R_0 = R_{3\text{-}11}$ prem.
901 | | | | | | | | ⊥ 40, 473, 545, 589, (*SPW I*)
902 | | | | | | | $(R_0 = R_{3\text{-}11}) \to \bot$ 900, 901, ($\to I$)
903 | | | | | | | | $R_0 = R_{3\text{-}12}$ prem.
904 | | | | | | | | ⊥ 41, 248, 249, 546, (*SPW I*)
905 | | | | | | | $(R_0 = R_{3\text{-}12}) \to \bot$ 903, 904, ($\to I$)
906 | | | | | | | | $R_0 = R_{3\text{-}13}$ prem.
907 | | | | | | | | ⊥ 42, 547, 574, 612, (*SPW I*)
908 | | | | | | | $(R_0 = R_{3\text{-}13}) \to \bot$ 906, 907, ($\to I$)
909 | | | | | | | | $R_0 = R_{4\text{-}1}$ prem.
910 | | | | | | | | ⊥ 43, 250, 590, 665, (*SPW I*)
911 | | | | | | | $(R_0 = R_{4\text{-}1}) \to \bot$ 909, 910, ($\to I$)
912 | | | | | | | | $R_0 = R_{4\text{-}2}$ prem.
913 | | | | | | | | ⊥ 44, 474, 591, 666, (*SPW I*)
914 | | | | | | | $(R_0 = R_{4\text{-}2}) \to \bot$ 912, 913, ($\to I$)
915 | | | | | | | | $R_0 = R_{4\text{-}3}$ prem.
916 | | | | | | | | ⊥ 45, 251, 252, 667, (*SPW I*)
917 | | | | | | | $(R_0 = R_{4\text{-}3}) \to \bot$ 915, 916, ($\to I$)
918 | | | | | | | | $R_0 = R_{4\text{-}4}$ prem.
919 | | | | | | | | ⊥ 46, 253–255, (*SPW I*)
920 | | | | | | | $(R_0 = R_{4\text{-}4}) \to \bot$ 918, 919, ($\to I$)
921 | | | | | | | | $R_0 = R_{4\text{-}5}$ prem.
922 | | | | | | | | ⊥ 47, 256, 475, 584, (*SPW I*)
923 | | | | | | | $(R_0 = R_{4\text{-}5}) \to \bot$ 921, 922, ($\to I$)
924 | | | | | | | | $R_0 = R_{4\text{-}6}$ prem.
925 | | | | | | | | ⊥ 48, 257, 258, 476, (*SPW I*)
926 | | | | | | | $(R_0 = R_{4\text{-}6}) \to \bot$ 924, 925, ($\to I$)
927 | | | | | | | | $R_0 = R_{4\text{-}7}$ prem.
928 | | | | | | | | ⊥ 49, 259, 613, 668, (*SPW I*)
929 | | | | | | | $(R_0 = R_{4\text{-}7}) \to \bot$ 927, 928, ($\to I$)
930 | | | | | | | | $R_0 = R_{4\text{-}8}$ prem.
931 | | | | | | | | ⊥ 50, 260, 477, 609, (*SPW I*)
932 | | | | | | | $(R_0 = R_{4\text{-}8}) \to \bot$ 930, 931, ($\to I$)
933 | | | | | | | | $R_0 = R_{4\text{-}9}$ prem.
934 | | | | | | | | ⊥ 51, 261, 262, 575, (*SPW I*)
935 | | | | | | | $(R_0 = R_{4\text{-}9}) \to \bot$ 933, 934, ($\to I$)
936 | | | | | | | | $R_0 = R_{4\text{-}10}$ prem.
937 | | | | | | | | ⊥ 52, 576, 592, 669, (*SPW I*)
938 | | | | | | | $(R_0 = R_{4\text{-}10}) \to \bot$ 936, 937, ($\to I$)
939 | | | | | | | | $R_0 = R_{4\text{-}11}$ prem.
940 | | | | | | | | ⊥ 53, 478, 593, 690, (*SPW I*)
941 | | | | | | | $(R_0 = R_{4\text{-}11}) \to \bot$ 939, 940, ($\to I$)
942 | | | | | | | | $R_0 = R_{4\text{-}12}$ prem.
943 | | | | | | | | ⊥ 54, 263, 264, 691, (*SPW I*)
944 | | | | | | | $(R_0 = R_{4\text{-}12}) \to \bot$ 942, 943, ($\to I$)
945 | | | | | | | | $R_0 = R_{4\text{-}13}$ prem.
946 | | | | | | | | ⊥ 55, 577, 614, 692, (*SPW I*)
947 | | | | | | | $(R_0 = R_{4\text{-}13}) \to \bot$ 945, 946, ($\to I$)
948 | | | | | | | | $R_0 = R_{5\text{-}1}$ prem.
949 | | | | | | | | ⊥ 56, 265, 630, 670, (*SPW I*)
950 | | | | | | | $(R_0 = R_{5\text{-}1}) \to \bot$ 948, 949, ($\to I$)
951 | | | | | | | | $R_0 = R_{5\text{-}2}$ prem.
952 | | | | | | | | ⊥ 57, 266, 267, 671, (*SPW I*)
953 | | | | | | | $(R_0 = R_{5\text{-}2}) \to \bot$ 951, 952, ($\to I$)
954 | | | | | | | | $R_0 = R_{5\text{-}3}$ prem.
955 | | | | | | | | ⊥ 58, 524, 631, 672, (*SPW I*)
956 | | | | | | | $(R_0 = R_{5\text{-}3}) \to \bot$ 954, 955, ($\to I$)
957 | | | | | | | | $R_0 = R_{5\text{-}4}$ prem.
958 | | | | | | | | ⊥ 59, 268, 525, 624, (*SPW I*)
959 | | | | | | | $(R_0 = R_{5\text{-}4}) \to \bot$ 957, 958, ($\to I$)
960 | | | | | | | | $R_0 = R_{5\text{-}5}$ prem.
961 | | | | | | | | ⊥ 60, 269–271, (*SPW I*)
962 | | | | | | | $(R_0 = R_{5\text{-}5}) \to \bot$ 960, 961, ($\to I$)
963 | | | | | | | | $R_0 = R_{5\text{-}6}$ prem.
964 | | | | | | | | ⊥ 61, 272, 273, 526, (*SPW I*)
965 | | | | | | | $(R_0 = R_{5\text{-}6}) \to \bot$ 963, 964, ($\to I$)
966 | | | | | | | | $R_0 = R_{5\text{-}7}$ prem.
967 | | | | | | | | ⊥ 62, 560, 632, 673, (*SPW I*)
968 | | | | | | | $(R_0 = R_{5\text{-}7}) \to \bot$ 966, 967, ($\to I$)
969 | | | | | | | | $R_0 = R_{5\text{-}8}$ prem.
970 | | | | | | | | ⊥ 63, 274, 275, 561, (*SPW I*)
971 | | | | | | | $(R_0 = R_{5\text{-}8}) \to \bot$ 969, 970, ($\to I$)
972 | | | | | | | | $R_0 = R_{5\text{-}9}$ prem.
973 | | | | | | | | ⊥ 64, 276, 527, 649, (*SPW I*)
974 | | | | | | | $(R_0 = R_{5\text{-}9}) \to \bot$ 972, 973, ($\to I$)
975 | | | | | | | | $R_0 = R_{5\text{-}10}$ prem.
976 | | | | | | | | ⊥ 65, 277, 653, 674, (*SPW I*)
977 | | | | | | | $(R_0 = R_{5\text{-}10}) \to \bot$ 975, 976, ($\to I$)
978 | | | | | | | | $R_0 = R_{5\text{-}11}$ prem.
979 | | | | | | | | ⊥ 66, 278, 279, 693, (*SPW I*)
980 | | | | | | | $(R_0 = R_{5\text{-}11}) \to \bot$ 978, 979, ($\to I$)
981 | | | | | | | | $R_0 = R_{5\text{-}12}$ prem.
982 | | | | | | | | ⊥ 67, 528, 633, 694, (*SPW I*)
983 | | | | | | | $(R_0 = R_{5\text{-}12}) \to \bot$ 981, 982, ($\to I$)
984 | | | | | | | | $R_0 = R_{5\text{-}13}$ prem.
985 | | | | | | | | ⊥ 68, 562, 654, 695, (*SPW I*)
986 | | | | | | | $(R_0 = R_{5\text{-}13}) \to \bot$ 984, 985, ($\to I$)
987 | | | | | | | | $R_0 = R_{6\text{-}1}$ prem.
988 | | | | | | | | ⊥ 69, 594, 634, 664, (*SPW I*)
989 | | | | | | | $(R_0 = R_{6\text{-}1}) \to \bot$ 987, 988, ($\to I$)
990 | | | | | | | | $R_0 = R_{6\text{-}2}$ prem.
991 | | | | | | | | ⊥ 70, 280, 595, 675, (*SPW I*)
992 | | | | | | | $(R_0 = R_{6\text{-}2}) \to \bot$ 990, 991, ($\to I$)
993 | | | | | | | | $R_0 = R_{6\text{-}3}$ prem.
994 | | | | | | | | ⊥ 71, 281, 635, 676, (*SPW I*)
995 | | | | | | | $(R_0 = R_{6\text{-}3}) \to \bot$ 993, 994, ($\to I$)
996 | | | | | | | | $R_0 = R_{6\text{-}4}$ prem.
997 | | | | | | | | ⊥ 72, 282, 283, 636, (*SPW I*)
998 | | | | | | | $(R_0 = R_{6\text{-}4}) \to \bot$ 996, 997, ($\to I$)
999 | | | | | | | | $R_0 = R_{6\text{-}5}$ prem.
1000 | | | | | | | | ⊥ 73, 284, 285, 596, (*SPW I*)
1001 | | | | | | | $(R_0 = R_{6\text{-}5}) \to \bot$ 999, 1000, ($\to I$)
1002 | | | | | | | | $R_0 = R_{6\text{-}6}$ prem.
1003 | | | | | | | | ⊥ 74, 286–288, (*SPW I*)
1004 | | | | | | | $(R_0 = R_{6\text{-}6}) \to \bot$ 1002, 1003, ($\to I$)
1005 | | | | | | | | $R_0 = R_{6\text{-}7}$ prem.
1006 | | | | | | | | ⊥ 75, 615, 637, 677, (*SPW I*)
1007 | | | | | | | $(R_0 = R_{6\text{-}7}) \to \bot$ 1005, 1006, ($\to I$)
1008 | | | | | | | | $R_0 = R_{6\text{-}8}$ prem.
1009 | | | | | | | | ⊥ 76, 289, 290, 616, (*SPW I*)
1010 | | | | | | | $(R_0 = R_{6\text{-}8}) \to \bot$ 1008, 1009, ($\to I$)
1011 | | | | | | | | $R_0 = R_{6\text{-}9}$ prem.
1012 | | | | | | | | ⊥ 77, 291, 292, 655, (*SPW I*)
1013 | | | | | | | $(R_0 = R_{6\text{-}9}) \to \bot$ 1011, 1012, ($\to I$)
1014 | | | | | | | | $R_0 = R_{6\text{-}10}$ prem.
1015 | | | | | | | | ⊥ 78, 597, 656, 678, (*SPW I*)
1016 | | | | | | | $(R_0 = R_{6\text{-}10}) \to \bot$ 1014, 1015, ($\to I$)
1017 | | | | | | | | $R_0 = R_{6\text{-}11}$ prem.
1018 | | | | | | | | ⊥ 79, 293, 598, 689, (*SPW I*)
1019 | | | | | | | $(R_0 = R_{6\text{-}11}) \to \bot$ 1017, 1018, ($\to I$)
1020 | | | | | | | | $R_0 = R_{6\text{-}12}$ prem.
1021 | | | | | | | | ⊥ 80, 294, 638, 696, (*SPW I*)
1022 | | | | | | | $(R_0 = R_{6\text{-}12}) \to \bot$ 1020, 1021, ($\to I$)
1023 | | | | | | | | $R_0 = R_{6\text{-}13}$ prem.
1024 | | | | | | | | ⊥ 81, 617, 657, 697, (*SPW I*)
1025 | | | | | | | $(R_0 = R_{6\text{-}13}) \to \bot$ 1023, 1024, ($\to I$)
1026 | | | | | | | | $R_0 = R_{7\text{-}1}$ prem.
1027 | | | | | | | | $\exists x \exists y \neg((A(x) \wedge A(y)) \to ((R_0(p,x,y) \wedge \neg R_0(p,y,x)) \to (R_0(s,x,y) \wedge \neg R_0(s,y,x))))$ 713, (rep.)
1028 | | | | | | | | | $\neg((A(d) \wedge A(e)) \to ((R_0(p,d,e) \wedge \neg R_0(p,e,d)) \to (R_0(s,d,e) \wedge \neg R_0(s,e,d))))$ prem.
1029 | | | | | | | | | $\neg((A(d) \wedge A(e)) \to ((R_{7\text{-}1}(p,d,e) \wedge \neg R_{7\text{-}1}(p,e,d)) \to (R_{7\text{-}1}(s,d,e) \wedge \neg R_{7\text{-}1}(s,e,d))))$ 1026, 1028, ($=E$)
1030 | | | | | | | | | | $A(d) \wedge A(e)$ prem.
1031 | | | | | | | | | | | $R_{7\text{-}1}(p,d,e) \wedge \neg R_{7\text{-}1}(p,e,d)$ prem.
1032 | | | | | | | | | | | | $\neg(R_{7\text{-}1}(s,d,e) \wedge \neg R_{7\text{-}1}(s,e,d))$ prem.
1033 | | | | | | | | | | | | $\forall x(A(x) \to (x=a \vee x=b \vee x=c))$ 2, ($\wedge E$)
1034 | | | | | | | | | | | | $A(d) \to (d=a \vee d=b \vee d=c)$ 1033, ($\forall E$)
1035 | | | | | | | | | | | | $A(e) \to (e=a \vee e=b \vee e=c)$ 1033, ($\forall E$)
1036 | | | | | | | | | | | | $A(d)$ 1030, ($\wedge E$)
1037 | | | | | | | | | | | | $d=a \vee d=b \vee d=c$ 1034, 1036, ($\to E$)
1038 | | | | | | | | | | | | $A(e)$ 1030, ($\wedge E$)
1039 | | | | | | | | | | | | $e=a \vee e=b \vee e=c$ 1035, 1038, ($\wedge E$), ($\to E$)
1040 | | | | | | | | | | | | | $d=a$ prem.
1041 | | | | | | | | | | | | | $e=a \vee e=b \vee e=c$ 1039, (rep.)
1042 | | | | | | | | | | | | | | $e=a$ prem.
1043 | | | | | | | | | | | | | | $R_{7\text{-}1}(p,a,a) \wedge \neg R_{7\text{-}1}(p,a,a)$ 1031, 1040, 1042, ($=E$)
1044 | | | | | | | | | | | | | | ⊥ 1043, ($\neg E$)
1045 | | | | | | | | | | | | | $(e=a) \to \bot$ 1042, 1044, ($\to I$)
1046 | | | | | | | | | | | | | | $e=b$ prem.
1047 | | | | | | | | | | | | | | $\neg R_{7\text{-}1}(p,e,d)$ 1031, ($\wedge E$)

1048 | | | | | | | | | | | | | | $\neg R_{7-1}(p, b, a)$ 1040, 1046, 1047, ($=E$)
1049 | | | | | | | | | | | | | | $R_{7-1}(p, b, a)$ 82, ($\wedge E$)
1050 | | | | | | | | | | | | | | $\bot$ 1048, 1049, ($\neg E$)
1051 | | | | | | | | | | | | | $(e = b) \to \bot$ 1046, 1050, ($\to I$)
1052 | | | | | | | | | | | | | | $e = c$ prem.
1053 | | | | | | | | | | | | | | $\neg(R_{7-1}(s, a, c) \wedge \neg R_{7-1}(s, c, a))$ 1032, 1040, 1052, ($=E$)
1054 | | | | | | | | | | | | | | $R_{7-1}(s, a, c) \wedge \neg R_{7-1}(s, c, a)$ 296, (rep.)
1055 | | | | | | | | | | | | | | $\bot$ 1053, 1054, ($\neg E$)
1056 | | | | | | | | | | | | | $(e = c) \to \bot$ 1052, 1055, ($\to I$)
1057 | | | | | | | | | | | | | $\bot$ 1041, 1045, 1051, 1056, ($\vee E$)
1058 | | | | | | | | | | | | $(d = a) \to \bot$ 1040, 1057, ($\to I$)
1059 | | | | | | | | | | | | | $d = b$ prem.
1060 | | | | | | | | | | | | | $e = a \vee e = b \vee e = c$ 1039, (rep.)
1061 | | | | | | | | | | | | | | $e = a$ prem.
1062 | | | | | | | | | | | | | | $\neg R_{7-1}(p, e, d)$ 1031, ($\wedge E$)
1063 | | | | | | | | | | | | | | $\neg R_{7-1}(p, a, b)$ 1059, 1061, 1062, ($=E$)
1064 | | | | | | | | | | | | | | $R_{7-1}(p, a, b)$ 82, ($\wedge E$)
1065 | | | | | | | | | | | | | | $\bot$ 1063, 1064, ($\neg E$)
1066 | | | | | | | | | | | | | $(e = a) \to \bot$ 1061, 1065, ($\to I$)
1067 | | | | | | | | | | | | | | $e = b$ prem.
1068 | | | | | | | | | | | | | | $R_{7-1}(p, b, b) \wedge \neg R_{7-1}(p, b, b)$ 1031, 1059, 1067, ($=E$)
1069 | | | | | | | | | | | | | | $\bot$ 1068, ($\neg E$)
1070 | | | | | | | | | | | | | $(e = b) \to \bot$ 1067, 1069, ($\to I$)
1071 | | | | | | | | | | | | | | $e = c$ prem.
1072 | | | | | | | | | | | | | | $\neg(R_{7-1}(s, b, c) \wedge \neg R_{7-1}(s, c, b))$ 1032, 1059, 1071, ($=E$)
1073 | | | | | | | | | | | | | | $R_{7-1}(s, b, c) \wedge \neg R_{7-1}(s, c, b)$ 295, (rep.)
1074 | | | | | | | | | | | | | | $\bot$ 1072, 1073, ($\neg E$)
1075 | | | | | | | | | | | | | $(e = c) \to \bot$ 1071, 1074, ($\to I$)
1076 | | | | | | | | | | | | | $\bot$ 1060, 1066, 1070, 1075, ($\vee E$)
1077 | | | | | | | | | | | | $(d = b) \to \bot$ 1059, 1076, ($\to I$)
1078 | | | | | | | | | | | | | $d = c$ prem.
1079 | | | | | | | | | | | | | $e = a \vee e = b \vee e = c$ 1039, (rep.)
1080 | | | | | | | | | | | | | | $e = a$ prem.
1081 | | | | | | | | | | | | | | $R_{7-1}(p, d, e)$ 1031, ($\wedge E$)
1082 | | | | | | | | | | | | | | $R_{7-1}(p, c, a)$ 1078, 1080, 1081, ($=E$)
1083 | | | | | | | | | | | | | | $\neg R_{7-1}(p, c, a)$ 82, ($\wedge E$)
1084 | | | | | | | | | | | | | | $\bot$ 1082, 1083, ($\neg E$)
1085 | | | | | | | | | | | | | $(e = a) \to \bot$ 1080, 1084, ($\to I$)
1086 | | | | | | | | | | | | | | $e = b$ prem.
1087 | | | | | | | | | | | | | | $R_{7-1}(p, d, e)$ 1031, ($\wedge E$)
1088 | | | | | | | | | | | | | | $R_{7-1}(p, c, b)$ 1078, 1086, 1087, ($=E$)
1089 | | | | | | | | | | | | | | $\neg R_{7-1}(p, c, b)$ 82, ($\wedge E$)
1090 | | | | | | | | | | | | | | $\bot$ 1088, 1089, ($\neg E$)
1091 | | | | | | | | | | | | | $(e = b) \to \bot$ 1086, 1090, ($\to I$)
1092 | | | | | | | | | | | | | | $e = c$ prem.
1093 | | | | | | | | | | | | | | $R_{7-1}(p, c, c) \wedge \neg R_{7-1}(p, c, c)$ 1031, 1078, 1092, ($=E$)
1094 | | | | | | | | | | | | | | $\bot$ 1093, ($\neg E$)
1095 | | | | | | | | | | | | | $(e = c) \to \bot$ 1092, 1094, ($\to I$)
1096 | | | | | | | | | | | | | $\bot$ 1079, 1085, 1091, 1095, ($\vee E$)
1097 | | | | | | | | | | | | $(d = c) \to \bot$ 1078, 1096, ($\to I$)
1098 | | | | | | | | | | | | $\bot$ 1037, 1058, 1077, 1097, ($\vee E$)
1099 | | | | | | | | | | | $R_{7-1}(s, d, e) \wedge \neg R_{7-1}(s, e, d)$ 1032, 1098, (DNE)
1100 | | | | | | | | | | $(R_{7-1}(p, d, e) \wedge \neg R_{7-1}(p, e, d)) \to (R_{7-1}(s, d, e) \wedge \neg R_{7-1}(s, e, d))$ 1031, 1099, ($\to I$)
1101 | | | | | | | | | $(A(d) \wedge A(e)) \to ((R_{7-1}(p, d, e) \wedge \neg R_{7-1}(p, e, d)) \to (R_{7-1}(s, d, e) \wedge \neg R_{7-1}(s, e, d)))$ 1030, 1100, ($\to I$)
1102 | | | | | | | | | $\bot$ 1029, 1101, ($\neg E$)
1103 | | | | | | | | $\bot$ 1027, 1102, ($\exists E$)
1104 | | | | | | | $(R_0 = R_{7-1}) \to \bot$ 1026, 1103, ($\to I$)
1105 | | | | | | | | $R_0 = R_{7-2}$ prem.
1106 | | | | | | | | $\bot$ 83, 297, 479, (similar procedure examining a whole profile II 1027–1103 [*SPW II*])
1107 | | | | | | | $(R_0 = R_{7-2}) \to \bot$ 1105, 1106, ($\to I$)
1108 | | | | | | | | $R_0 = R_{7-3}$ prem.
1109 | | | | | | | | $\bot$ 84, 298, 299, (*SPW II*)
1110 | | | | | | | $(R_0 = R_{7-3}) \to \bot$ 1108, 1109, ($\to I$)
1111 | | | | | | | | $R_0 = R_{7-4}$ prem.
1112 | | | | | | | | $\bot$ 85, 300, 504, (*SPW II*)
1113 | | | | | | | $(R_0 = R_{7-4}) \to \bot$ 1111, 1112, ($\to I$)
1114 | | | | | | | | $R_0 = R_{7-5}$ prem.
1115 | | | | | | | | $\bot$ 86, 480, 505, (*SPW II*)
1116 | | | | | | | $(R_0 = R_{7-5}) \to \bot$ 1114, 1115, ($\to I$)
1117 | | | | | | | | $R_0 = R_{7-6}$ prem.
1118 | | | | | | | | $\bot$ 87, 481, 506, (*SPW II*)
1119 | | | | | | | $(R_0 = R_{7-6}) \to \bot$ 1117, 1118, ($\to I$)
1120 | | | | | | | | $R_0 = R_{7-7}$ prem.
1121 | | | | | | | | $\bot$ 88, 301, 302, (*SPW II*)
1122 | | | | | | | $(R_0 = R_{7-7}) \to \bot$ 1120, 1121, ($\to I$)
1123 | | | | | | | | $R_0 = R_{7-8}$ prem.
1124 | | | | | | | | $\bot$ 89, 482, 507, (*SPW II*)
1125 | | | | | | | $(R_0 = R_{7-8}) \to \bot$ 1123, 1124, ($\to I$)
1126 | | | | | | | | $R_0 = R_{7-9}$ prem.
1127 | | | | | | | | $\bot$ 90, 508, 578, (*SPW II*)
1128 | | | | | | | $(R_0 = R_{7-9}) \to \bot$ 1126, 1127, ($\to I$)
1129 | | | | | | | | $R_0 = R_{7-10}$ prem.
1130 | | | | | | | | $\bot$ 91, 303, 579, (*SPW II*)
1131 | | | | | | | $(R_0 = R_{7-10}) \to \bot$ 1129, 1130, ($\to I$)
1132 | | | | | | | | $R_0 = R_{7-11}$ prem.
1133 | | | | | | | | $\bot$ 92, 483, 548, (*SPW II*)
1134 | | | | | | | $(R_0 = R_{7-11}) \to \bot$ 1132, 1133, ($\to I$)
1135 | | | | | | | | $R_0 = R_{7-12}$ prem.
1136 | | | | | | | | $\bot$ 93, 304, 549, (*SPW II*)
1137 | | | | | | | $(R_0 = R_{7-12}) \to \bot$ 1135, 1136, ($\to I$)
1138 | | | | | | | | $R_0 = R_{7-13}$ prem.
1139 | | | | | | | | $\bot$ 94, 550, 580, (*SPW II*)
1140 | | | | | | | $(R_0 = R_{7-13}) \to \bot$ 1138, 1139, ($\to I$)
1141 | | | | | | | | $R_0 = R_{8-1}$ prem.
1142 | | | | | | | | $\bot$ 95, 639, 679, (*SPW II*)
1143 | | | | | | | $(R_0 = R_{8-1}) \to \bot$ 1141, 1142, ($\to I$)
1144 | | | | | | | | $R_0 = R_{8-2}$ prem.
1145 | | | | | | | | $\bot$ 96, 305, 680, (*SPW II*)
1146 | | | | | | | $(R_0 = R_{8-2}) \to \bot$ 1144, 1145, ($\to I$)
1147 | | | | | | | | $R_0 = R_{8-3}$ prem.
1148 | | | | | | | | $\bot$ 97, 640, 681, (*SPW II*)
1149 | | | | | | | $(R_0 = R_{8-3}) \to \bot$ 1147, 1148, ($\to I$)
1150 | | | | | | | | $R_0 = R_{8-4}$ prem.
1151 | | | | | | | | $\bot$ 98, 306, 641, (*SPW II*)
1152 | | | | | | | $(R_0 = R_{8-4}) \to \bot$ 1150, 1151, ($\to I$)
1153 | | | | | | | | $R_0 = R_{8-5}$ prem.
1154 | | | | | | | | $\bot$ 99, 307, 308, (*SPW II*)
1155 | | | | | | | $(R_0 = R_{8-5}) \to \bot$ 1153, 1154, ($\to I$)
1156 | | | | | | | | $R_0 = R_{8-6}$ prem.
1157 | | | | | | | | $\bot$ 100, 309, 310, (*SPW II*)
1158 | | | | | | | $(R_0 = R_{8-6}) \to \bot$ 1156, 1157, ($\to I$)
1159 | | | | | | | | $R_0 = R_{8-7}$ prem.
1160 | | | | | | | | $\bot$ 101, 642, 682, (*SPW II*)
1161 | | | | | | | $(R_0 = R_{8-7}) \to \bot$ 1159, 1160, ($\to I$)
1162 | | | | | | | | $R_0 = R_{8-8}$ prem.
1163 | | | | | | | | $\bot$ 102, 311, 312, (*SPW II*)
1164 | | | | | | | $(R_0 = R_{8-8}) \to \bot$ 1162, 1163, ($\to I$)
1165 | | | | | | | | $R_0 = R_{8-9}$ prem.
1166 | | | | | | | | $\bot$ 103, 313, 658, (*SPW II*)
1167 | | | | | | | $(R_0 = R_{8-9}) \to \bot$ 1165, 1166, ($\to I$)
1168 | | | | | | | | $R_0 = R_{8-10}$ prem.
1169 | | | | | | | | $\bot$ 104, 659, 683, (*SPW II*)
1170 | | | | | | | $(R_0 = R_{8-10}) \to \bot$ 1168, 1169, ($\to I$)
1171 | | | | | | | | $R_0 = R_{8-11}$ prem.
1172 | | | | | | | | $\bot$ 105, 314, 698, (*SPW II*)
1173 | | | | | | | $(R_0 = R_{8-11}) \to \bot$ 1171, 1172, ($\to I$)
1174 | | | | | | | | $R_0 = R_{8-12}$ prem.
1175 | | | | | | | | $\bot$ 106, 643, 699, (*SPW II*)
1176 | | | | | | | $(R_0 = R_{8-12}) \to \bot$ 1174, 1175, ($\to I$)
1177 | | | | | | | | $R_0 = R_{8-13}$ prem.
1178 | | | | | | | | $\bot$ 107, 660, 700, (*SPW II*)
1179 | | | | | | | $(R_0 = R_{8-13}) \to \bot$ 1177, 1178, ($\to I$)
1180 | | | | | | | | $R_0 = R_{9-1}$ prem.
1181 | | | | | | | | $\bot$ 108, 599, 684, (*SPW II*)
1182 | | | | | | | $(R_0 = R_{9-1}) \to \bot$ 1180, 1181, ($\to I$)
1183 | | | | | | | | $R_0 = R_{9-2}$ prem.
1184 | | | | | | | | $\bot$ 109, 600, 685, (*SPW II*)
1185 | | | | | | | $(R_0 = R_{9-2}) \to \bot$ 1183, 1184, ($\to I$)
1186 | | | | | | | | $R_0 = R_{9-3}$ prem.
1187 | | | | | | | | $\bot$ 110, 315, 686, (*SPW II*)
1188 | | | | | | | $(R_0 = R_{9-3}) \to \bot$ 1186, 1187, ($\to I$)
1189 | | | | | | | | $R_0 = R_{9-4}$ prem.
1190 | | | | | | | | $\bot$ 111, 316, 317, (*SPW II*)
1191 | | | | | | | $(R_0 = R_{9-4}) \to \bot$ 1189, 1190, ($\to I$)
1192 | | | | | | | | $R_0 = R_{9-5}$ prem.
1193 | | | | | | | | $\bot$ 112, 318, 601, (*SPW II*)
1194 | | | | | | | $(R_0 = R_{9-5}) \to \bot$ 1192, 1193, ($\to I$)
1195 | | | | | | | | $R_0 = R_{9-6}$ prem.
1196 | | | | | | | | $\bot$ 113, 319, 320, (*SPW II*)
1197 | | | | | | | $(R_0 = R_{9-6}) \to \bot$ 1195, 1194, ($\to I$)
1198 | | | | | | | | $R_0 = R_{9-7}$ prem.

1199 | | | | | | | | $\bot$ 114, 618, 687, (*SPW II*)
1200 | | | | | | | $(R_0 = R_{9\text{-}7}) \rightarrow \bot$ 1198, 1199, ($\rightarrow I$)
1201 | | | | | | | | $R_0 = R_{9\text{-}8}$ prem.
1202 | | | | | | | | $\bot$ 115, 321, 619, (*SPW II*)
1203 | | | | | | | $(R_0 = R_{9\text{-}8}) \rightarrow \bot$ 1201, 1202, ($\rightarrow I$)
1204 | | | | | | | | $R_0 = R_{9\text{-}9}$ prem.
1205 | | | | | | | | $\bot$ 116, 322, 323, (*SPW II*)
1206 | | | | | | | $(R_0 = R_{9\text{-}9}) \rightarrow \bot$ 1204, 1205, ($\rightarrow I$)
1207 | | | | | | | | $R_0 = R_{9\text{-}10}$ prem.
1208 | | | | | | | | $\bot$ 117, 602, 688, (*SPW II*)
1209 | | | | | | | $(R_0 = R_{9\text{-}10}) \rightarrow \bot$ 1207, 1208, ($\rightarrow I$)
1210 | | | | | | | | $R_0 = R_{9\text{-}11}$ prem.
1211 | | | | | | | | $\bot$ 118, 603, 701, (*SPW II*)
1212 | | | | | | | $(R_0 = R_{9\text{-}11}) \rightarrow \bot$ 1210, 1211, ($\rightarrow I$)
1213 | | | | | | | | $R_0 = R_{9\text{-}12}$ prem.
1214 | | | | | | | | $\bot$ 119, 324, 702, (*SPW II*)
1215 | | | | | | | $(R_0 = R_{9\text{-}12}) \rightarrow \bot$ 1213, 1214, ($\rightarrow I$)
1216 | | | | | | | | $R_0 = R_{9\text{-}13}$ prem.
1217 | | | | | | | | $\bot$ 120, 620, 703, (*SPW II*)
1218 | | | | | | | $(R_0 = R_{9\text{-}13}) \rightarrow \bot$ 1216, 1217, ($\rightarrow I$)
1219 | | | | | | | | $R_0 = R_{10\text{-}1}$ prem.
1220 | | | | | | | | $\bot$ 121, 325, 326, (*SPW II*)
1221 | | | | | | | $(R_0 = R_{10\text{-}1}) \rightarrow \bot$ 1219, 1220, ($\rightarrow I$)
1222 | | | | | | | | $R_0 = R_{10\text{-}2}$ prem.
1223 | | | | | | | | $\bot$ 122, 327, 328, (*SPW II*)
1224 | | | | | | | $(R_0 = R_{10\text{-}2}) \rightarrow \bot$ 1222, 1223, ($\rightarrow I$)
1225 | | | | | | | | $R_0 = R_{10\text{-}3}$ prem.
1226 | | | | | | | | $\bot$ 123, 329, 529, (*SPW II*)
1227 | | | | | | | $(R_0 = R_{10\text{-}3}) \rightarrow \bot$ 1225, 1226, ($\rightarrow I$)
1228 | | | | | | | | $R_0 = R_{10\text{-}4}$ prem.
1229 | | | | | | | | $\bot$ 124, 509, 530, (*SPW II*)
1230 | | | | | | | $(R_0 = R_{10\text{-}4}) \rightarrow \bot$ 1228, 1229, ($\rightarrow I$)
1231 | | | | | | | | $R_0 = R_{10\text{-}5}$ prem.
1232 | | | | | | | | $\bot$ 125, 330, 510, (*SPW II*)
1233 | | | | | | | $(R_0 = R_{10\text{-}5}) \rightarrow \bot$ 1231, 1232, ($\rightarrow I$)
1234 | | | | | | | | $R_0 = R_{10\text{-}6}$ prem.
1235 | | | | | | | | $\bot$ 126, 511, 531, (*SPW II*)
1236 | | | | | | | $(R_0 = R_{10\text{-}6}) \rightarrow \bot$ 1234, 1235, ($\rightarrow I$)
1237 | | | | | | | | $R_0 = R_{10\text{-}7}$ prem.
1238 | | | | | | | | $\bot$ 127, 331, 563, (*SPW II*)
1239 | | | | | | | $(R_0 = R_{10\text{-}7}) \rightarrow \bot$ 1237, 1238, ($\rightarrow I$)
1240 | | | | | | | | $R_0 = R_{10\text{-}8}$ prem.
1241 | | | | | | | | $\bot$ 128, 512, 564, (*SPW II*)
1242 | | | | | | | $(R_0 = R_{10\text{-}8}) \rightarrow \bot$ 1240, 1241, ($\rightarrow I$)
1243 | | | | | | | | $R_0 = R_{10\text{-}9}$ prem.
1244 | | | | | | | | $\bot$ 129, 513, 532, (*SPW II*)
1245 | | | | | | | $(R_0 = R_{10\text{-}9}) \rightarrow \bot$ 1243, 1244, ($\rightarrow I$)
1246 | | | | | | | | $R_0 = R_{10\text{-}10}$ prem.
1247 | | | | | | | | $\bot$ 130, 332, 333, (*SPW II*)
1248 | | | | | | | $(R_0 = R_{10\text{-}10}) \rightarrow \bot$ 1246, 1247, ($\rightarrow I$)
1249 | | | | | | | | $R_0 = R_{10\text{-}11}$ prem.
1250 | | | | | | | | $\bot$ 131, 334, 551, (*SPW II*)
1251 | | | | | | | $(R_0 = R_{10\text{-}11}) \rightarrow \bot$ 1249, 1250, ($\rightarrow I$)
1252 | | | | | | | | $R_0 = R_{10\text{-}12}$ prem.
1253 | | | | | | | | $\bot$ 132, 533, 552, (*SPW II*)
1254 | | | | | | | $(R_0 = R_{10\text{-}12}) \rightarrow \bot$ 1252, 1253, ($\rightarrow I$)
1255 | | | | | | | | $R_0 = R_{10\text{-}13}$ prem.
1256 | | | | | | | | $\bot$ 133, 553, 565, (*SPW II*)
1257 | | | | | | | $(R_0 = R_{10\text{-}13}) \rightarrow \bot$ 1255, 1256, ($\rightarrow I$)
1258 | | | | | | | | $R_0 = R_{11\text{-}1}$ prem.
1259 | | | | | | | | $\bot$ 134, 335, 644, (*SPW II*)
1260 | | | | | | | $(R_0 = R_{11\text{-}1}) \rightarrow \bot$ 1258, 1259, ($\rightarrow I$)
1261 | | | | | | | | $R_0 = R_{11\text{-}2}$ prem.
1262 | | | | | | | | $\bot$ 135, 336, 337, (*SPW II*)
1263 | | | | | | | $(R_0 = R_{11\text{-}2}) \rightarrow \bot$ 1261, 1262, ($\rightarrow I$)
1264 | | | | | | | | $R_0 = R_{11\text{-}3}$ prem.
1265 | | | | | | | | $\bot$ 136, 534, 645, (*SPW II*)
1266 | | | | | | | $(R_0 = R_{11\text{-}3}) \rightarrow \bot$ 1264, 1265, ($\rightarrow I$)
1267 | | | | | | | | $R_0 = R_{11\text{-}4}$ prem.
1268 | | | | | | | | $\bot$ 137, 535, 646, (*SPW II*)
1269 | | | | | | | $(R_0 = R_{11\text{-}4}) \rightarrow \bot$ 1267, 1268, ($\rightarrow I$)
1270 | | | | | | | | $R_0 = R_{11\text{-}5}$ prem.
1271 | | | | | | | | $\bot$ 138, 338, 339, (*SPW II*)
1272 | | | | | | | $(R_0 = R_{11\text{-}5}) \rightarrow \bot$ 1270, 1271, ($\rightarrow I$)
1273 | | | | | | | | $R_0 = R_{11\text{-}6}$ prem.
1274 | | | | | | | | $\bot$ 139, 340, 536, (*SPW II*)
1275 | | | | | | | $(R_0 = R_{11\text{-}6}) \rightarrow \bot$ 1273, 1274, ($\rightarrow I$)
1276 | | | | | | | | $R_0 = R_{11\text{-}7}$ prem.
1277 | | | | | | | | $\bot$ 140, 566, 647, (*SPW II*)
1278 | | | | | | | $(R_0 = R_{11\text{-}7}) \rightarrow \bot$ 1276, 1277, ($\rightarrow I$)
1279 | | | | | | | | $R_0 = R_{11\text{-}8}$ prem.
1280 | | | | | | | | $\bot$ 141, 341, 567, (*SPW II*)
1281 | | | | | | | $(R_0 = R_{11\text{-}8}) \rightarrow \bot$ 1279, 1280, ($\rightarrow I$)
1282 | | | | | | | | $R_0 = R_{11\text{-}9}$ prem.
1283 | | | | | | | | $\bot$ 142, 537, 661, (*SPW II*)
1284 | | | | | | | $(R_0 = R_{11\text{-}9}) \rightarrow \bot$ 1282, 1283, ($\rightarrow I$)
1285 | | | | | | | | $R_0 = R_{11\text{-}10}$ prem.
1286 | | | | | | | | $\bot$ 143, 342, 662, (*SPW II*)
1287 | | | | | | | $(R_0 = R_{11\text{-}10}) \rightarrow \bot$ 1285, 1286, ($\rightarrow I$)
1288 | | | | | | | | $R_0 = R_{11\text{-}11}$ prem.
1289 | | | | | | | | $\bot$ 144, 343, 344, (*SPW II*)
1290 | | | | | | | $(R_0 = R_{11\text{-}11}) \rightarrow \bot$ 1288, 1289, ($\rightarrow I$)
1291 | | | | | | | | $R_0 = R_{11\text{-}12}$ prem.
1292 | | | | | | | | $\bot$ 145, 538, 648, (*SPW II*)
1293 | | | | | | | $(R_0 = R_{11\text{-}12}) \rightarrow \bot$ 1291, 1292, ($\rightarrow I$)
1294 | | | | | | | | $R_0 = R_{11\text{-}13}$ prem.
1295 | | | | | | | | $\bot$ 146, 568, 663, (*SPW II*)
1296 | | | | | | | $(R_0 = R_{11\text{-}13}) \rightarrow \bot$ 1294, 1295, ($\rightarrow I$)
1297 | | | | | | | | $R_0 = R_{12\text{-}1}$ prem.
1298 | | | | | | | | $\bot$ 147, 345, 604, (*SPW II*)
1299 | | | | | | | $(R_0 = R_{12\text{-}1}) \rightarrow \bot$ 1297, 1299, ($\rightarrow I$)
1300 | | | | | | | | $R_0 = R_{12\text{-}2}$ prem.
1301 | | | | | | | | $\bot$ 148, 484, 605, (*SPW II*)
1302 | | | | | | | $(R_0 = R_{12\text{-}2}) \rightarrow \bot$ 1300, 1301, ($\rightarrow I$)
1303 | | | | | | | | $R_0 = R_{12\text{-}3}$ prem.
1304 | | | | | | | | $\bot$ 149, 346, 347, (*SPW II*)
1305 | | | | | | | $(R_0 = R_{12\text{-}3}) \rightarrow \bot$ 1303, 1304, ($\rightarrow I$)
1306 | | | | | | | | $R_0 = R_{12\text{-}4}$ prem.
1307 | | | | | | | | $\bot$ 150, 348, 349, (*SPW II*)
1308 | | | | | | | $(R_0 = R_{12\text{-}4}) \rightarrow \bot$ 1306, 1307, ($\rightarrow I$)
1309 | | | | | | | | $R_0 = R_{12\text{-}5}$ prem.
1310 | | | | | | | | $\bot$ 151, 485, 606, (*SPW II*)
1311 | | | | | | | $(R_0 = R_{12\text{-}5}) \rightarrow \bot$ 1309, 1310, ($\rightarrow I$)
1312 | | | | | | | | $R_0 = R_{12\text{-}6}$ prem.
1313 | | | | | | | | $\bot$ 152, 350, 486, (*SPW II*)
1314 | | | | | | | $(R_0 = R_{12\text{-}6}) \rightarrow \bot$ 1312, 1313, ($\rightarrow I$)
1315 | | | | | | | | $R_0 = R_{12\text{-}7}$ prem.
1316 | | | | | | | | $\bot$ 153, 351, 621, (*SPW II*)
1317 | | | | | | | $(R_0 = R_{12\text{-}7}) \rightarrow \bot$ 1315, 1316, ($\rightarrow I$)
1318 | | | | | | | | $R_0 = R_{12\text{-}8}$ prem.
1319 | | | | | | | | $\bot$ 154, 487, 622, (*SPW II*)
1320 | | | | | | | $(R_0 = R_{12\text{-}8}) \rightarrow \bot$ 1318, 1319, ($\rightarrow I$)
1321 | | | | | | | | $R_0 = R_{12\text{-}9}$ prem.
1322 | | | | | | | | $\bot$ 155, 352, 581, (*SPW II*)
1323 | | | | | | | $(R_0 = R_{12\text{-}9}) \rightarrow \bot$ 1321, 1322, ($\rightarrow I$)
1324 | | | | | | | | $R_0 = R_{12\text{-}10}$ prem.
1325 | | | | | | | | $\bot$ 156, 582, 607, (*SPW II*)
1326 | | | | | | | $(R_0 = R_{12\text{-}10}) \rightarrow \bot$ 1324, 1325, ($\rightarrow I$)
1327 | | | | | | | | $R_0 = R_{12\text{-}11}$ prem.
1328 | | | | | | | | $\bot$ 157, 488, 608, (*SPW II*)
1329 | | | | | | | $(R_0 = R_{12\text{-}11}) \rightarrow \bot$ 1327, 1328, ($\rightarrow I$)
1330 | | | | | | | | $R_0 = R_{12\text{-}12}$ prem.
1331 | | | | | | | | $\bot$ 158, 353, 354, (*SPW II*)
1332 | | | | | | | $(R_0 = R_{12\text{-}12}) \rightarrow \bot$ 1330, 1331, ($\rightarrow I$)
1333 | | | | | | | | $R_0 = R_{12\text{-}13}$ prem.
1334 | | | | | | | | $\bot$ 159, 583, 623, (*SPW II*)
1335 | | | | | | | $(R_0 = R_{12\text{-}13}) \rightarrow \bot$ 1333, 1334, ($\rightarrow I$)
1336 | | | | | | | | $R_0 = R_{13\text{-}1}$ prem.
1337 | | | | | | | | $\exists x \exists y \neg((A(x) \wedge A(y)) \rightarrow ((R_0(p,x,y) \wedge \neg R_0(p,y,x)) \rightarrow (R_0(s,x,y) \wedge \neg R_0(s,y,x)))))$ 713, (rep.)
1338 | | | | | | | | | $\neg((A(d) \wedge A(e)) \rightarrow ((R_0(p,d,e) \wedge \neg R_0(p,e,d)) \rightarrow (R_0(s,d,e) \wedge \neg R_0(s,e,d)))))$ prem.
1339 | | | | | | | | | $\neg((A(d) \wedge A(e)) \rightarrow ((R_{13\text{-}1}(p,d,e) \wedge \neg R_{13\text{-}1}(p,e,d)) \rightarrow (R_{13\text{-}1}(s,d,e) \wedge \neg R_{13\text{-}1}(s,e,d)))))$ 1336, 1338, ($=E$)
1340 | | | | | | | | | | $(A(d) \wedge A(e))$ prem.
1341 | | | | | | | | | | | $R_{13\text{-}1}(p,d,e) \wedge \neg R_{13\text{-}1}(p,e,d)$ prem.
1342 | | | | | | | | | | | | $\neg(R_{13\text{-}1}(s,d,e) \wedge \neg R_{13\text{-}1}(s,e,d))$ prem.
1343 | | | | | | | | | | | | $\forall x(A(x) \rightarrow (x = a \vee x = b \vee x = c))$ 2, ($\wedge E$)
1344 | | | | | | | | | | | | $A(d) \rightarrow (d = a \vee d = b \vee d = c)$ 1343, ($\forall E$)
1345 | | | | | | | | | | | | $A(e) \rightarrow (e = a \vee e = b \vee e = c)$ 1343, ($\forall E$)
1346 | | | | | | | | | | | | $A(d)$ 1340, ($\wedge E$)
1347 | | | | | | | | | | | | $d = a \vee d = b \vee d = c$ 1344, 1346, ($\rightarrow E$)
1348 | | | | | | | | | | | | $A(e)$ 1340, ($\wedge E$)
1349 | | | | | | | | | | | | $e = a \vee e = b \vee e = c$ 1345, 1348, ($\wedge E$), ($\rightarrow E$)

1350 $d = a$ prem.
1351 $e = a \vee e = b \vee e = c$ 1349, (rep.)
1352 $e = a$ prem.
1353 $R_{13-1}(p, a, a) \wedge \neg R_{13-1}(p, a, a)$ 1341, 1350, 1352, $(=E)$
1354 $\bot$ 1353, $(\neg E)$
1355 $(e = a) \to \bot$ 1352, 1354, $(\to I)$
1356 $e = b$ prem.
1357 $\neg R_{13-1}(p, e, d))$ 1341, $(\wedge E)$
1358 $\neg R_{13-1}(p, b, a)$ 1350, 1356, 1357, $(=E)$
1359 $R_{13-1}(p, b, a)$ 160, $(\wedge E)$
1360 $\bot$ 1358, 1359, $(\neg E)$
1361 $(e = b) \to \bot$ 1356, 1360, $(\to I)$
1362 $e = c$ prem.
1363 $\neg R_{13-1}(p, e, d))$ 1341, $(\wedge E)$
1364 $\neg R_{13-1}(p, c, a)$ 1350, 1362, 1363, $(=E)$
1365 $R_{13-1}(s, c, a)$ 160, $(\wedge E)$
1366 $\bot$ 1364, 1365, $(\neg E)$
1367 $(e = c) \to \bot$ 1362, 1366, $(\to I)$
1368 $\bot$ 1351, 1355, 1361, 1367, $(\vee E)$
1369 $(d = a) \to \bot$ 1350, 1368, $(\to I)$
1370 $d = b$ prem.
1371 $e = a \vee e = b \vee e = c$ 1349, (rep.)
1372 $e = a$ prem.
1373 $\neg R_{13-1}(p, e, d)$ 1341, $(\wedge E)$
1374 $\neg R_{13-1}(p, a, b)$ 1370, 1372, 1373 $(=E)$
1375 $R_{13-1}(p, a, b)$ 160, $(\wedge E)$
1376 $\bot$ 1374, 1375, $(\neg E)$
1377 $(e = a) \to \bot$ 1372, 1376, $(\to I)$
1378 $e = b$ prem.
1379 $R_{13-1}(p, b, b) \wedge \neg R_{13-1}(p, b, b)$ 1341, 1370, 1378, $(=E)$
1380 $\bot$ 1379, $(\neg E)$
1381 $(e = b) \to \bot$ 1378, 1380, $(\to I)$
1382 $e = c$ prem.
1383 $\neg R_{13-1}(p, e, d)$ 1341, $(\wedge E)$
1384 $\neg R_{13-1}(p, c, b)$ 1370, 1382, 1383 $(=E)$
1385 $R_{13-1}(p, c, b)$ 160, $(\wedge E)$
1386 $\bot$ 1384, 1385, $(\neg E)$
1387 $(e = c) \to \bot$ 1382, 1386, $(\to I)$
1388 $\bot$ 1371, 1377, 1381, 1387, $(\vee E)$
1389 $(d = b) \to \bot$ 1370, 1388, $(\to I)$
1390 $d = c$ prem.
1391 $e = a \vee e = b \vee e = c$ 1349, (rep.)
1392 $e = a$ prem.
1393 $\neg R_{13-1}(p, e, d)$ 1341, $(\wedge E)$
1394 $\neg R_{13-1}(p, a, c)$ 1390, 1392, 1393, $(=E)$
1395 $R_{13-1}(p, c, a)$ 160, $(\wedge E)$
1396 $\bot$ 1394, 1395, $(\neg E)$
1397 $(e = a) \to \bot$ 1392, 1396, $(\to I)$
1398 $e = b$ prem.
1399 $\neg R_{13-1}(p, e, d)$ 1341, $(\wedge E)$
1400 $\neg R_{13-1}(p, b, c)$ 1390, 1398, 1399, $(=E)$
1401 $R_{13-1}(p, b, c)$ 160, $(\wedge E)$
1402 $\bot$ 1400, 1401, $(\neg E)$
1403 $(e = b) \to \bot$ 1398, 1402, $(\to I)$
1404 $e = c$ prem.
1405 $R_{13-1}(p, c, c) \wedge \neg R_{13-1}(p, c, c)$ 1341, 1390, 1404, $(=E)$
1406 $\bot$ 1405, $(\neg E)$
1407 $(e = c) \to \bot$ 1404, 1406, $(\to I)$
1408 $\bot$ 1391, 1397, 1403, 1407, $(\vee E)$
1409 $(d = c) \to \bot$ 1390, 1408, $(\to I)$
1410 $\bot$ 1347, 1369, 1389, 1409, $(\vee E)$
1411 $R_{13-1}(s, d, e) \wedge \neg R_{13-1}(s, e, d)$ 1342, 1410, (DNE)
1412 $(R_{13-1}(p, d, e) \wedge \neg R_{13-1}(p, e, d)) \to (R_{13-1}(s, d, e) \wedge \neg R_{13-1}(s, e, d))$ 1341, 1411, $(\to I)$
1413 $(A(d) \wedge A(e)) \to ((R_{13-1}(p, d, e) \wedge \neg R_{13-1}(p, e, d)) \to (R_{13-1}(s, d, e) \wedge \neg R_{13-1}(s, e, d)))$ 1340, 1412, $(\to I)$
1414 $\bot$ 1339, 1413, $(\neg E)$
1415 $\bot$ 1337, 1414, $(\exists E)$
1416 $(R_0 = R_{13-1}) \to \bot$ 1336, 1415, $(\to I)$
1417 $R_0 = R_{13-2}$ prem.
1418 $\bot$ 161, (similar procedure examining a whole profile III 1337–1415 [*SPW III*])
1419 $(R_0 = R_{13-2}) \to \bot$ 1417, 1418, $(\to I)$
1420 $R_0 = R_{13-3}$ prem.
1421 $\bot$ 162, (*SPW III*)
1422 $(R_0 = R_{13-3}) \to \bot$ 1420, 1421, $(\to I)$
1423 $R_0 = R_{13-4}$ prem.
1424 $\bot$ 163, (*SPW III*)
1425 $(R_0 = R_{13-4}) \to \bot$ 1423, 1424, $(\to I)$
1426 $R_0 = R_{13-5}$ prem.
1427 $\bot$ 164, (*SPW III*)
1428 $(R_0 = R_{13-5}) \to \bot$ 1426, 1427, $(\to I)$
1429 $R_0 = R_{13-6}$ prem.
1430 $\bot$ 165, (*SPW III*)
1431 $(R_0 = R_{13-6}) \to \bot$ 1429, 1430, $(\to I)$
1432 $R_0 = R_{13-7}$ prem.
1433 $\bot$ 166, (*SPW III*)
1434 $(R_0 = R_{13-7}) \to \bot$ 1432, 1433, $(\to I)$
1435 $R_0 = R_{13-8}$ prem.
1436 $\bot$ 167, (*SPW III*)
1437 $(R_0 = R_{13-8}) \to \bot$ 1435, 1436, $(\to I)$
1438 $R_0 = R_{13-9}$ prem.
1439 $\bot$ 168, (*SPW III*)
1440 $(R_0 = R_{13-9}) \to \bot$ 1438, 1439, $(\to I)$
1441 $R_0 = R_{13-10}$ prem.
1442 $\bot$ 169, (*SPW III*)
1443 $(R_0 = R_{13-10}) \to \bot$ 1441, 1442, $(\to I)$
1444 $R_0 = R_{13-11}$ prem.
1445 $\bot$ 170, (*SPW III*)
1446 $(R_0 = R_{13-11}) \to \bot$ 1444, 1445, $(\to I)$
1447 $R_0 = R_{13-12}$ prem.
1448 $\bot$ 171, (*SPW III*)
1449 $(R_0 = R_{13-12}) \to \bot$ 1447, 1448, $(\to I)$
1450 $R_0 = R_{13-13}$ prem.
1451 $\bot$ 172, (*SPW III*)
1452 $(R_0 = R_{13-13}) \to \bot$ 1450, 1451, $(\to I)$
1453 $\bot$ 716, formulas $(R_0 = R_{1-1}) \to \bot, \ldots, (R_0 = R_{13-13}) \to \bot$from 794 to 1452, $(\vee E)$
1454 $\forall x \forall y((A(x) \wedge A(y)) \to ((R_0(p, x, y) \wedge \neg R_0(p, y, x)) \to (R_0(s, x, y) \wedge \neg R_0(s, y, x)))))$ 713, 1453, (DNE)
1455 $\boldsymbol{P}(R_0) \to \forall x \forall y((A(x) \wedge A(y)) \to ((R_0(p, x, y) \wedge \neg R_0(p, y, x)) \to (R_0(s, x, y) \wedge \neg R_0(s, y, x)))))$ 712, 1454, $(\to I)$
1456 $\forall X(\boldsymbol{P}(X) \to \forall x \forall y((A(x) \wedge A(y)) \to ((X(p, x, y) \wedge \neg X(p, y, x)) \to (X(s, x, y) \wedge \neg X(s, y, x)))))$ 1455, $(\forall I)$
1457 $\bot$ 711, 1456, $(\neg E)$
1458 $\neg \forall X(\boldsymbol{P}(X) \to \forall x \forall y((A(x) \wedge A(y)) \to ((X(p, x, y) \wedge \neg X(p, y, x)) \to (X(s, x, y) \wedge \neg X(s, y, x))))) \to \bot$ 711, 1457, $(\to I)$
1459 $\neg \forall u(H(u) \to (\forall X(\boldsymbol{P}(X) \to \forall x \forall y((A(x) \wedge A(y)) \to ((X(u, x, y) \wedge \neg X(u, y, x)) \to (X(s, x, y) \wedge \neg X(s, y, x))))) \to u = p))$ prem.
1460 $H(h)$ prem.
1461 $\forall X(\boldsymbol{P}(X) \to \forall x \forall y((A(x) \wedge A(y)) \to ((X(h, x, y) \wedge \neg X(h, y, x)) \to (X(s, x, y) \wedge \neg X(s, y, x)))))$ prem.
1462 $h \neq p$ prem.
1463 $\forall x(H(x) \to (x = p \vee x = q))$ 1, $(\wedge E)$
1464 $H(h) \to (h = p \vee h = q)$ 1463, $(\forall E)$
1465 $h = p \vee h = q$ 1460, 1464, $(\to E)$
1466 $h = p$ prem.
1467 $p \neq p$ 1462, 1466, $(=E)$
1468 $\bot$ 1467, $(\neg E)$
1469 $(h = p) \to \bot$ 1466, 1468, $(\to I)$
1470 $h = q$ prem.
1471 $\forall X(\boldsymbol{P}(X) \to \forall x \forall y((A(x) \wedge A(y)) \to ((X(q, x, y) \wedge \neg X(q, y, x)) \to (X(s, x, y) \wedge \neg X(s, y, x)))))$ 1461, 1470, $(=E)$
1472 $\boldsymbol{P}(R_{1-2}) \to \forall x \forall y((A(x) \wedge A(y)) \to ((R_{1-2}(q, x, y) \wedge \neg R_{1-2}(q, y, x)) \to (R_{1-2}(s, x, y) \wedge \neg R_{1-2}(s, y, x))))$ 1471, $(\forall E)$
1473 $\boldsymbol{P}(R_{1-2})$ 3, $(\wedge E)$
1474 $\forall x \forall y((A(x) \wedge A(y)) \to ((R_{1-2}(q, x, y) \wedge \neg R_{1-2}(q, y, x)) \to (R_{1-2}(s, x, y) \wedge \neg R_{1-2}(s, y, x))))$ 1472, 1473, $(\to E)$
1475 $(A(c) \wedge A(b)) \to ((R_{1-2}(q, c, b) \wedge \neg R_{1-2}(q, b, c)) \to (R_{1-2}(s, c, b) \wedge \neg R_{1-2}(s, b, c)))$ 1474, $(\forall E)$
1476 $A(c) \wedge A(b)$ 2, $(\wedge E)$
1477 $(R_{1-2}(q, c, b) \wedge \neg R_{1-2}(q, b, c)) \to (R_{1-2}(s, c, b) \wedge \neg R_{1-2}(s, b, c))$ 1475, 1476, $(\to E)$
1478 $R_{1-2}(q, c, b) \wedge \neg R_{1-2}(q, b, c)$ 5, $(\wedge E)$
1479 $R_{1-2}(s, c, b) \wedge \neg R_{1-2}(s, b, c)$ 1477, 1478, $(\to E)$
1480 $R_{1-2}(s, c, b)$ 1479, $(\wedge E)$
1481 $\bot$ 464, 1480, $(\neg E)$
1482 $(h = q) \to \bot$ 1470, 1481, $(\to I)$
1483 $\bot$ 1465, 1469, 1482, $(\vee E)$
1484 $h = p$ 1462, 1483, (DNE)
1485 $\forall X(\boldsymbol{P}(X) \to \forall x \forall y((A(x) \wedge A(y)) \to ((X(h, x, y) \wedge \neg X(h, y, x)) \to (X(s, x, y) \wedge \neg X(s, y, x))))) \to h = p$ 1461, 1484, $(\to I)$
1486 $H(h) \to (\forall X(\boldsymbol{P}(X) \to \forall x \forall y((A(x) \wedge A(y)) \to ((X(h, x, y) \wedge \neg X(h, y, x)) \to (X(s, x, y) \wedge \neg X(s, y, x))))) \to h = p)$ 1460, 1485, $(\to I)$
1487 $\forall u(H(u) \to (\forall X(\boldsymbol{P}(X) \to \forall x \forall y((A(x) \wedge A(y)) \to ((X(u, x, y) \wedge \neg X(u, y, x)) \to (X(s, x, y) \wedge \neg X(s, y, x))))) \to u = p))$ 1486, $(\forall I)$
1488 $\bot$ 1459, 1487, $(\neg E)$
1489 $\neg \forall u(H(u) \to (\forall X(\boldsymbol{P}(X) \to \forall x \forall y((A(x) \wedge A(y)) \to ((X(u, x, y) \wedge \neg X(u, y, x)) \to (X(s, x, y) \wedge \neg X(s, y, x))))) \to u = p)) \to \bot$ 1459, 1488, $(\to I)$
1490 $\bot$ 706, 710, 1458, 1489, $(\vee E)$
1491 $\neg R_{1-2}(s, c, b) \to \bot$ 464, 1490, $(\to I)$
1492 $\bot$ 363, 463, 1491, $(\vee E)$
1493 $R_{1-2}(s, b, c) \to \bot$ 362, $(\to I)$
1494 $\neg R_{1-2}(s, b, c)$ prem.
1495 $R_{1-2}(s, c, b) \vee \neg R_{1-2}(s, c, b)$ 360, $(\wedge E)$
1496 $R_{1-2}(s, c, b)$ prem.
1497 $\neg R_{1-5}(s, b, c) \wedge R_{1-5}(s, c, b)$ 1494, 1496, (SPI)
1498 $\neg R_{1-6}(s, b, c) \wedge R_{1-6}(s, c, b)$ 1494, 1496, (SPI)
1499 $\neg R_{1-8}(s, b, c) \wedge R_{1-8}(s, c, b)$ 1494, 1496, (SPI)
1500 $\neg R_{1-11}(s, b, c) \wedge R_{1-11}(s, c, b)$ 1494, 1496, (SPI)

1501 | | | | $\neg R_{3-2}(s,b,c) \wedge R_{3-2}(s,c,b)$ 1494, 1496, (*SPI*)
1502 | | | | $\neg R_{3-5}(s,b,c) \wedge R_{3-5}(s,c,b)$ 1494, 1496, (*SPI*)
1503 | | | | $\neg R_{3-6}(s,b,c) \wedge R_{3-6}(s,c,b)$ 1494, 1496, (*SPI*)
1504 | | | | $\neg R_{3-8}(s,b,c) \wedge R_{3-8}(s,c,b)$ 1494, 1496, (*SPI*)
1505 | | | | $\neg R_{3-11}(s,b,c) \wedge R_{3-11}(s,c,b)$ 1494, 1496, (*SPI*)
1506 | | | | $\neg R_{4-2}(s,b,c) \wedge R_{4-2}(s,c,b)$ 1494, 1496, (*SPI*)
1507 | | | | $\neg R_{4-5}(s,b,c) \wedge R_{4-5}(s,c,b)$ 1494, 1496, (*SPI*)
1508 | | | | $\neg R_{4-6}(s,b,c) \wedge R_{4-6}(s,c,b)$ 1494, 1496, (*SPI*)
1509 | | | | $\neg R_{4-8}(s,b,c) \wedge R_{4-8}(s,c,b)$ 1494, 1496, (*SPI*)
1510 | | | | $\neg R_{4-11}(s,b,c) \wedge R_{4-11}(s,c,b)$ 1494, 1496, (*SPI*)
1511 | | | | $\neg R_{7-2}(s,b,c) \wedge R_{7-2}(s,c,b)$ 1494, 1496, (*SPI*)
1512 | | | | $\neg R_{7-5}(s,b,c) \wedge R_{7-5}(s,c,b)$ 1494, 1496, (*SPI*)
1513 | | | | $\neg R_{7-6}(s,b,c) \wedge R_{7-6}(s,c,b)$ 1494, 1496, (*SPI*)
1514 | | | | $\neg R_{7-8}(s,b,c) \wedge R_{7-8}(s,c,b)$ 1494, 1496, (*SPI*)
1515 | | | | $\neg R_{7-11}(s,b,c) \wedge R_{7-11}(s,c,b)$ 1494, 1496, (*SPI*)
1516 | | | | $\neg R_{12-2}(s,b,c) \wedge R_{12-2}(s,c,b)$ 1494, 1496, (*SPI*)
1517 | | | | $\neg R_{12-5}(s,b,c) \wedge R_{12-5}(s,c,b)$ 1494, 1496, (*SPI*)
1518 | | | | $\neg R_{12-6}(s,b,c) \wedge R_{12-6}(s,c,b)$ 1494, 1496, (*SPI*)
1519 | | | | $\neg R_{12-8}(s,b,c) \wedge R_{12-8}(s,c,b)$ 1494, 1496, (*SPI*)
1520 | | | | $\neg R_{12-11}(s,b,c) \wedge R_{12-11}(s,c,b)$ 1494, 1496, (*SPI*)
1521 | | | | $R_{3-2}(s,a,b) \wedge \neg R_{3-2}(s,b,a)$ 237, 1501, (*SPT*)
1522 | | | | $R_{3-1}(s,a,b) \wedge \neg R_{3-1}(s,b,a)$ 1521, (*SPI*)
1523 | | | | $R_{3-5}(s,a,b) \wedge \neg R_{3-5}(s,b,a)$ 1521, (*SPI*)
1524 | | | | $R_{3-10}(s,a,b) \wedge \neg R_{3-10}(s,b,a)$ 1521, (*SPI*)
1525 | | | | $R_{3-11}(s,a,b) \wedge \neg R_{3-11}(s,b,a)$ 1521, (*SPI*)
1526 | | | | $R_{4-1}(s,a,b) \wedge \neg R_{4-1}(s,b,a)$ 1521, (*SPI*)
1527 | | | | $R_{4-2}(s,a,b) \wedge \neg R_{4-2}(s,b,a)$ 1521, (*SPI*)
1528 | | | | $R_{4-5}(s,a,b) \wedge \neg R_{4-5}(s,b,a)$ 1521, (*SPI*)
1529 | | | | $R_{4-10}(s,a,b) \wedge \neg R_{4-10}(s,b,a)$ 1521, (*SPI*)
1530 | | | | $R_{4-11}(s,a,b) \wedge \neg R_{4-11}(s,b,a)$ 1521, (*SPI*)
1531 | | | | $R_{6-1}(s,a,b) \wedge \neg R_{6-1}(s,b,a)$ 1521, (*SPI*)
1532 | | | | $R_{6-2}(s,a,b) \wedge \neg R_{6-2}(s,b,a)$ 1521, (*SPI*)
1533 | | | | $R_{6-5}(s,a,b) \wedge \neg R_{6-5}(s,b,a)$ 1521, (*SPI*)
1534 | | | | $R_{6-10}(s,a,b) \wedge \neg R_{6-10}(s,b,a)$ 1521, (*SPI*)
1535 | | | | $R_{6-11}(s,a,b) \wedge \neg R_{6-11}(s,b,a)$ 1521, (*SPI*)
1536 | | | | $R_{9-1}(s,a,b) \wedge \neg R_{9-1}(s,b,a)$ 1521, (*SPI*)
1537 | | | | $R_{9-2}(s,a,b) \wedge \neg R_{9-2}(s,b,a)$ 1521, (*SPI*)
1538 | | | | $R_{9-5}(s,a,b) \wedge \neg R_{9-5}(s,b,a)$ 1521, (*SPI*)
1539 | | | | $R_{9-10}(s,a,b) \wedge \neg R_{9-10}(s,b,a)$ 1521, (*SPI*)
1540 | | | | $R_{9-11}(s,a,b) \wedge \neg R_{9-11}(s,b,a)$ 1521, (*SPI*)
1541 | | | | $R_{12-1}(s,a,b) \wedge \neg R_{12-1}(s,b,a)$ 1521, (*SPI*)
1542 | | | | $R_{12-2}(s,a,b) \wedge \neg R_{12-2}(s,b,a)$ 1521, (*SPI*)
1543 | | | | $R_{12-5}(s,a,b) \wedge \neg R_{12-5}(s,b,a)$ 1521, (*SPI*)
1544 | | | | $R_{12-10}(s,a,b) \wedge \neg R_{12-10}(s,b,a)$ 1521, (*SPI*)
1545 | | | | $R_{12-11}(s,a,b) \wedge \neg R_{12-11}(s,b,a)$ 1521, (*SPI*)
1546 | | | | $\neg R_{3-6}(s,a,c) \wedge R_{3-6}(s,c,a)$ 243, 1503, (*SPT*)
1547 | | | | $\neg R_{1-4}(s,a,c) \wedge R_{1-4}(s,c,a)$ 1546, (*SPI*)
1548 | | | | $\neg R_{1-5}(s,a,c) \wedge R_{1-5}(s,c,a)$ 1546, (*SPI*)
1549 | | | | $\neg R_{1-6}(s,a,c) \wedge R_{1-6}(s,c,a)$ 1546, (*SPI*)
1550 | | | | $\neg R_{1-8}(s,a,c) \wedge R_{1-8}(s,c,a)$ 1546, (*SPI*)
1551 | | | | $\neg R_{1-9}(s,a,c) \wedge R_{1-9}(s,c,a)$ 1546, (*SPI*)
1552 | | | | $\neg R_{2-4}(s,a,c) \wedge R_{2-4}(s,c,a)$ 1546, (*SPI*)
1553 | | | | $\neg R_{2-5}(s,a,c) \wedge R_{2-5}(s,c,a)$ 1546, (*SPI*)
1554 | | | | $\neg R_{2-6}(s,a,c) \wedge R_{2-6}(s,c,a)$ 1546, (*SPI*)
1555 | | | | $\neg R_{2-8}(s,a,c) \wedge R_{2-8}(s,c,a)$ 1546, (*SPI*)
1556 | | | | $\neg R_{2-9}(s,a,c) \wedge R_{2-9}(s,c,a)$ 1546, (*SPI*)
1557 | | | | $\neg R_{3-4}(s,a,c) \wedge R_{3-4}(s,c,a)$ 1546, (*SPI*)
1558 | | | | $\neg R_{3-5}(s,a,c) \wedge R_{3-5}(s,c,a)$ 1546, (*SPI*)
1559 | | | | $\neg R_{3-8}(s,a,c) \wedge R_{3-8}(s,c,a)$ 1546, (*SPI*)
1560 | | | | $\neg R_{3-9}(s,a,c) \wedge R_{3-9}(s,c,a)$ 1546, (*SPI*)
1561 | | | | $\neg R_{7-4}(s,a,c) \wedge R_{7-4}(s,c,a)$ 1546, (*SPI*)
1562 | | | | $\neg R_{7-5}(s,a,c) \wedge R_{7-5}(s,c,a)$ 1546, (*SPI*)
1563 | | | | $\neg R_{7-6}(s,a,c) \wedge R_{7-6}(s,c,a)$ 1546, (*SPI*)
1564 | | | | $\neg R_{7-8}(s,a,c) \wedge R_{7-8}(s,c,a)$ 1546, (*SPI*)
1565 | | | | $\neg R_{7-9}(s,a,c) \wedge R_{7-9}(s,c,a)$ 1546, (*SPI*)
1566 | | | | $\neg R_{10-4}(s,a,c) \wedge R_{10-4}(s,c,a)$ 1546, (*SPI*)
1567 | | | | $\neg R_{10-5}(s,a,c) \wedge R_{10-5}(s,c,a)$ 1546, (*SPI*)
1568 | | | | $\neg R_{10-6}(s,a,c) \wedge R_{10-6}(s,c,a)$ 1546, (*SPI*)
1569 | | | | $\neg R_{10-8}(s,a,c) \wedge R_{10-8}(s,c,a)$ 1546, (*SPI*)
1570 | | | | $\neg R_{10-9}(s,a,c) \wedge R_{10-9}(s,c,a)$ 1546, (*SPI*)
1571 | | | | $R_{4-1}(s,a,c) \wedge \neg R_{4-1}(s,c,a)$ 250, 1526, (*SPT*)
1572 | | | | $R_{4-2}(s,a,c) \wedge \neg R_{4-2}(s,c,a)$ 1571, (*SPI*)
1573 | | | | $R_{4-3}(s,a,c) \wedge \neg R_{4-3}(s,c,a)$ 1571, (*SPI*)
1574 | | | | $R_{4-7}(s,a,c) \wedge \neg R_{4-7}(s,c,a)$ 1571, (*SPI*)
1575 | | | | $R_{4-10}(s,a,c) \wedge \neg R_{4-10}(s,c,a)$ 1571, (*SPI*)
1576 | | | | $R_{5-1}(s,a,c) \wedge \neg R_{5-1}(s,c,a)$ 1571, (*SPI*)
1577 | | | | $R_{5-2}(s,a,c) \wedge \neg R_{5-2}(s,c,a)$ 1571, (*SPI*)
1578 | | | | $R_{5-3}(s,a,c) \wedge \neg R_{5-3}(s,c,a)$ 1571, (*SPI*)
1579 | | | | $R_{5-7}(s,a,c) \wedge \neg R_{5-7}(s,c,a)$ 1571, (*SPI*)
1580 | | | | $R_{5-10}(s,a,c) \wedge \neg R_{5-10}(s,c,a)$ 1571, (*SPI*)
1581 | | | | $R_{6-1}(s,a,c) \wedge \neg R_{6-1}(s,c,a)$ 1571, (*SPI*)
1582 | | | | $R_{6-2}(s,a,c) \wedge \neg R_{6-2}(s,c,a)$ 1571, (*SPI*)
1583 | | | | $R_{6-3}(s,a,c) \wedge \neg R_{6-3}(s,c,a)$ 1571, (*SPI*)
1584 | | | | $R_{6-7}(s,a,c) \wedge \neg R_{6-7}(s,c,a)$ 1571, (*SPI*)
1585 | | | | $R_{6-10}(s,a,c) \wedge \neg R_{6-10}(s,c,a)$ 1571, (*SPI*)
1586 | | | | $R_{8-1}(s,a,c) \wedge \neg R_{8-1}(s,c,a)$ 1571, (*SPI*)
1587 | | | | $R_{8-2}(s,a,c) \wedge \neg R_{8-2}(s,c,a)$ 1571, (*SPI*)
1588 | | | | $R_{8-3}(s,a,c) \wedge \neg R_{8-3}(s,c,a)$ 1571, (*SPI*)
1589 | | | | $R_{8-7}(s,a,c) \wedge \neg R_{8-7}(s,c,a)$ 1571, (*SPI*)
1590 | | | | $R_{8-10}(s,a,c) \wedge \neg R_{8-10}(s,c,a)$ 1571, (*SPI*)
1591 | | | | $R_{9-1}(s,a,c) \wedge \neg R_{9-1}(s,c,a)$ 1571, (*SPI*)
1592 | | | | $R_{9-2}(s,a,c) \wedge \neg R_{9-2}(s,c,a)$ 1571, (*SPI*)
1593 | | | | $R_{9-3}(s,a,c) \wedge \neg R_{9-3}(s,c,a)$ 1571, (*SPI*)
1594 | | | | $R_{9-7}(s,a,c) \wedge \neg R_{9-7}(s,c,a)$ 1571, (*SPI*)
1595 | | | | $R_{9-10}(s,a,c) \wedge \neg R_{9-10}(s,c,a)$ 1571, (*SPI*)
1596 | | | | $R_{6-3}(s,b,c) \wedge \neg R_{6-3}(s,c,b)$ 281, 1583, (*SPT*)
1597 | | | | $R_{2-1}(s,b,c) \wedge \neg R_{2-1}(s,c,b)$ 1596, (*SPI*)
1598 | | | | $R_{2-3}(s,b,c) \wedge \neg R_{2-3}(s,c,b)$ 1596, (*SPI*)
1599 | | | | $R_{2-4}(s,b,c) \wedge \neg R_{2-4}(s,c,b)$ 1596, (*SPI*)
1600 | | | | $R_{2-7}(s,b,c) \wedge \neg R_{2-7}(s,c,b)$ 1596, (*SPI*)
1601 | | | | $R_{2-12}(s,b,c) \wedge \neg R_{2-12}(s,c,b)$ 1596, (*SPI*)
1602 | | | | $R_{5-1}(s,b,c) \wedge \neg R_{5-1}(s,c,b)$ 1596, (*SPI*)
1603 | | | | $R_{5-3}(s,b,c) \wedge \neg R_{5-3}(s,c,b)$ 1596, (*SPI*)
1604 | | | | $R_{5-4}(s,b,c) \wedge \neg R_{5-4}(s,c,b)$ 1596, (*SPI*)
1605 | | | | $R_{5-7}(s,b,c) \wedge \neg R_{5-7}(s,c,b)$ 1596, (*SPI*)
1606 | | | | $R_{5-12}(s,b,c) \wedge \neg R_{5-12}(s,c,b)$ 1596, (*SPI*)
1607 | | | | $R_{6-1}(s,b,c) \wedge \neg R_{6-1}(s,c,b)$ 1596, (*SPI*)
1608 | | | | $R_{6-4}(s,b,c) \wedge \neg R_{6-4}(s,c,b)$ 1596, (*SPI*)
1609 | | | | $R_{6-7}(s,b,c) \wedge \neg R_{6-7}(s,c,b)$ 1596, (*SPI*)
1610 | | | | $R_{6-12}(s,b,c) \wedge \neg R_{6-12}(s,c,b)$ 1596, (*SPI*)
1611 | | | | $R_{8-1}(s,b,c) \wedge \neg R_{8-1}(s,c,b)$ 1596, (*SPI*)
1612 | | | | $R_{8-3}(s,b,c) \wedge \neg R_{8-3}(s,c,b)$ 1596, (*SPI*)
1613 | | | | $R_{8-4}(s,b,c) \wedge \neg R_{8-4}(s,c,b)$ 1596, (*SPI*)
1614 | | | | $R_{8-7}(s,b,c) \wedge \neg R_{8-7}(s,c,b)$ 1596, (*SPI*)
1615 | | | | $R_{8-12}(s,b,c) \wedge \neg R_{8-12}(s,c,b)$ 1596, (*SPI*)
1616 | | | | $R_{11-1}(s,b,c) \wedge \neg R_{11-1}(s,c,b)$ 1596, (*SPI*)
1617 | | | | $R_{11-3}(s,b,c) \wedge \neg R_{11-3}(s,c,b)$ 1596, (*SPI*)
1618 | | | | $R_{11-4}(s,b,c) \wedge \neg R_{11-4}(s,c,b)$ 1596, (*SPI*)
1619 | | | | $R_{11-7}(s,b,c) \wedge \neg R_{11-7}(s,c,b)$ 1596, (*SPI*)
1620 | | | | $R_{11-12}(s,b,c) \wedge \neg R_{11-12}(s,c,b)$ 1596, (*SPI*)
1621 | | | | $R_{7-2}(s,a,b) \wedge \neg R_{7-2}(s,b,a)$ 297, 1511, (*SPT*)
1622 | | | | $R_{7-1}(s,a,b) \wedge \neg R_{7-1}(s,b,a)$ 1621, (*SPI*)
1623 | | | | $R_{7-5}(s,a,b) \wedge \neg R_{7-5}(s,b,a)$ 1621, (*SPI*)
1624 | | | | $R_{7-10}(s,a,b) \wedge \neg R_{7-10}(s,b,a)$ 1621, (*SPI*)
1625 | | | | $R_{7-11}(s,a,b) \wedge \neg R_{7-11}(s,b,a)$ 1621, (*SPI*)
1626 | | | | $R_{8-1}(s,a,b) \wedge \neg R_{8-1}(s,b,a)$ 1621, (*SPI*)
1627 | | | | $R_{8-2}(s,a,b) \wedge \neg R_{8-2}(s,b,a)$ 1621, (*SPI*)
1628 | | | | $R_{8-5}(s,a,b) \wedge \neg R_{8-5}(s,b,a)$ 1621, (*SPI*)
1629 | | | | $R_{8-10}(s,a,b) \wedge \neg R_{8-10}(s,b,a)$ 1621, (*SPI*)
1630 | | | | $R_{8-11}(s,a,b) \wedge \neg R_{8-11}(s,b,a)$ 1621, (*SPI*)
1631 | | | | $R_{13-1}(s,a,b) \wedge \neg R_{13-1}(s,b,a)$ 1621, (*SPI*)
1632 | | | | $R_{13-2}(s,a,b) \wedge \neg R_{13-2}(s,b,a)$ 1621, (*SPI*)
1633 | | | | $R_{13-5}(s,a,b) \wedge \neg R_{13-5}(s,b,a)$ 1621, (*SPI*)
1634 | | | | $R_{13-10}(s,a,b) \wedge \neg R_{13-10}(s,b,a)$ 1621, (*SPI*)
1635 | | | | $R_{13-11}(s,a,b) \wedge \neg R_{13-11}(s,b,a)$ 1621, (*SPI*)
1636 | | | | $\neg R_{7-4}(s,a,b) \wedge R_{7-4}(s,b,a)$ 300, 1561, (*SPT*)
1637 | | | | $\neg R_{7-3}(s,a,b) \wedge R_{7-3}(s,b,a)$ 1636, (*SPI*)
1638 | | | | $\neg R_{7-6}(s,a,b) \wedge R_{7-6}(s,b,a)$ 1636, (*SPI*)
1639 | | | | $\neg R_{7-9}(s,a,b) \wedge R_{7-9}(s,b,a)$ 1636, (*SPI*)
1640 | | | | $\neg R_{7-12}(s,a,b) \wedge R_{7-12}(s,b,a)$ 1636, (*SPI*)
1641 | | | | $\neg R_{8-3}(s,a,b) \wedge R_{8-3}(s,b,a)$ 1636, (*SPI*)
1642 | | | | $\neg R_{8-4}(s,a,b) \wedge R_{8-4}(s,b,a)$ 1636, (*SPI*)
1643 | | | | $\neg R_{8-6}(s,a,b) \wedge R_{8-6}(s,b,a)$ 1636, (*SPI*)
1644 | | | | $\neg R_{8-9}(s,a,b) \wedge R_{8-9}(s,b,a)$ 1636, (*SPI*)
1645 | | | | $\neg R_{8-12}(s,a,b) \wedge R_{8-12}(s,b,a)$ 1636, (*SPI*)
1646 | | | | $\neg R_{13-3}(s,a,b) \wedge R_{13-3}(s,b,a)$ 1636, (*SPI*)
1647 | | | | $\neg R_{13-4}(s,a,b) \wedge R_{13-4}(s,b,a)$ 1636, (*SPI*)
1648 | | | | $\neg R_{13-6}(s,a,b) \wedge R_{13-6}(s,b,a)$ 1636, (*SPI*)
1649 | | | | $\neg R_{13-9}(s,a,b) \wedge R_{13-9}(s,b,a)$ 1636, (*SPI*)
1650 | | | | $\neg R_{13-12}(s,a,b) \wedge R_{13-12}(s,b,a)$ 1636, (*SPI*)
1651 | | | | $R_{9-3}(s,b,c) \wedge \neg R_{9-3}(s,c,b)$ 315, 1593, (*SPT*)

1652 | | | | $R_{9-1}(s,b,c) \land \neg R_{9-1}(s,c,b)$ 1651, (*SPI*)
1653 | | | | $R_{9-4}(s,b,c) \land \neg R_{9-4}(s,c,b)$ 1651, (*SPI*)
1654 | | | | $R_{9-7}(s,b,c) \land \neg R_{9-7}(s,c,b)$ 1651, (*SPI*)
1655 | | | | $R_{9-12}(s,b,c) \land \neg R_{9-12}(s,c,b)$ 1651, (*SPI*)
1656 | | | | $R_{10-1}(s,b,c) \land \neg R_{10-1}(s,c,b)$ 1651, (*SPI*)
1657 | | | | $R_{10-3}(s,b,c) \land \neg R_{10-3}(s,c,b)$ 1651, (*SPI*)
1658 | | | | $R_{10-4}(s,b,c) \land \neg R_{10-4}(s,c,b)$ 1651, (*SPI*)
1659 | | | | $R_{10-7}(s,b,c) \land \neg R_{10-7}(s,c,b)$ 1651, (*SPI*)
1660 | | | | $R_{10-12}(s,b,c) \land \neg R_{10-12}(s,c,b)$ 1651, (*SPI*)
1661 | | | | $R_{13-1}(s,b,c) \land \neg R_{13-1}(s,c,b)$ 1651, (*SPI*)
1662 | | | | $R_{13-3}(s,b,c) \land \neg R_{13-3}(s,c,b)$ 1651, (*SPI*)
1663 | | | | $R_{13-4}(s,b,c) \land \neg R_{13-4}(s,c,b)$ 1651, (*SPI*)
1664 | | | | $R_{13-7}(s,b,c) \land \neg R_{13-7}(s,c,b)$ 1651, (*SPI*)
1665 | | | | $R_{13-12}(s,b,c) \land \neg R_{13-12}(s,c,b)$ 1651, (*SPI*)
1666 | | | | $\neg R_{9-5}(s,b,c) \land R_{9-5}(s,c,b)$ 318, 1538, (*SPT*)
1667 | | | | $\neg R_{9-2}(s,b,c) \land R_{9-2}(s,c,b)$ 1666, (*SPI*)
1668 | | | | $\neg R_{9-6}(s,b,c) \land R_{9-6}(s,c,b)$ 1666, (*SPI*)
1669 | | | | $\neg R_{9-8}(s,b,c) \land R_{9-8}(s,c,b)$ 1666, (*SPI*)
1670 | | | | $\neg R_{9-11}(s,b,c) \land R_{9-11}(s,c,b)$ 1666, (*SPI*)
1671 | | | | $\neg R_{10-2}(s,b,c) \land R_{10-2}(s,c,b)$ 1666, (*SPI*)
1672 | | | | $\neg R_{10-5}(s,b,c) \land R_{10-5}(s,c,b)$ 1666, (*SPI*)
1673 | | | | $\neg R_{10-6}(s,b,c) \land R_{10-6}(s,c,b)$ 1666, (*SPI*)
1674 | | | | $\neg R_{10-8}(s,b,c) \land R_{10-8}(s,c,b)$ 1666, (*SPI*)
1675 | | | | $\neg R_{10-11}(s,b,c) \land R_{10-11}(s,c,b)$ 1666, (*SPI*)
1676 | | | | $\neg R_{13-2}(s,b,c) \land R_{13-2}(s,c,b)$ 1666, (*SPI*)
1677 | | | | $\neg R_{13-5}(s,b,c) \land R_{13-5}(s,c,b)$ 1666, (*SPI*)
1678 | | | | $\neg R_{13-6}(s,b,c) \land R_{13-6}(s,c,b)$ 1666, (*SPI*)
1679 | | | | $\neg R_{13-8}(s,b,c) \land R_{13-8}(s,c,b)$ 1666, (*SPI*)
1680 | | | | $\neg R_{13-11}(s,b,c) \land R_{13-11}(s,c,b)$ 1666, (*SPI*)
1681 | | | | $\neg R_{10-4}(s,a,b) \land R_{10-4}(s,b,a)$ 1566, 1658, (SPT)
1682 | | | | $\neg R_{1-3}(s,a,b) \land R_{1-3}(s,b,a)$ 1681, (*SPI*)
1683 | | | | $\neg R_{1-4}(s,a,b) \land R_{1-4}(s,b,a)$ 1681, (*SPI*)
1684 | | | | $\neg R_{1-6}(s,a,b) \land R_{1-6}(s,b,a)$ 1681, (*SPI*)
1685 | | | | $\neg R_{1-9}(s,a,b) \land R_{1-9}(s,b,a)$ 1681, (*SPI*)
1686 | | | | $\neg R_{1-12}(s,a,b) \land R_{1-12}(s,b,a)$ 1681, (*SPI*)
1687 | | | | $\neg R_{2-3}(s,a,b) \land R_{2-3}(s,b,a)$ 1681, (*SPI*)
1688 | | | | $\neg R_{2-4}(s,a,b) \land R_{2-4}(s,b,a)$ 1681, (*SPI*)
1689 | | | | $\neg R_{2-6}(s,a,b) \land R_{2-6}(s,b,a)$ 1681, (*SPI*)
1690 | | | | $\neg R_{2-9}(s,a,b) \land R_{2-9}(s,b,a)$ 1681, (*SPI*)
1691 | | | | $\neg R_{2-12}(s,a,b) \land R_{2-12}(s,b,a)$ 1681, (*SPI*)
1692 | | | | $\neg R_{5-3}(s,a,b) \land R_{5-3}(s,b,a)$ 1681, (*SPI*)
1693 | | | | $\neg R_{5-4}(s,a,b) \land R_{5-4}(s,b,a)$ 1681, (*SPI*)
1694 | | | | $\neg R_{5-6}(s,a,b) \land R_{5-6}(s,b,a)$ 1681, (*SPI*)
1695 | | | | $\neg R_{5-9}(s,a,b) \land R_{5-9}(s,b,a)$ 1681, (*SPI*)
1696 | | | | $\neg R_{5-12}(s,a,b) \land R_{5-12}(s,b,a)$ 1681, (*SPI*)
1697 | | | | $\neg R_{10-3}(s,a,b) \land R_{10-3}(s,b,a)$ 1681, (*SPI*)
1698 | | | | $\neg R_{10-6}(s,a,b) \land R_{10-6}(s,b,a)$ 1681, (*SPI*)
1699 | | | | $\neg R_{10-9}(s,a,b) \land R_{10-9}(s,b,a)$ 1681, (*SPI*)
1700 | | | | $\neg R_{10-12}(s,a,b) \land R_{10-12}(s,b,a)$ 1681, (*SPI*)
1701 | | | | $\neg R_{11-3}(s,a,b) \land R_{11-3}(s,b,a)$ 1681, (*SPI*)
1702 | | | | $\neg R_{11-4}(s,a,b) \land R_{11-4}(s,b,a)$ 1681, (*SPI*)
1703 | | | | $\neg R_{11-6}(s,a,b) \land R_{11-6}(s,b,a)$ 1681, (*SPI*)
1704 | | | | $\neg R_{11-9}(s,a,b) \land R_{11-9}(s,b,a)$ 1681, (*SPI*)
1705 | | | | $\neg R_{11-12}(s,a,b) \land R_{11-12}(s,b,a)$ 1681, (*SPI*)
1706 | | | | $R_{11-1}(s,a,c) \land \neg R_{11-1}(s,c,a)$ 335, 1616, (*SPT*)
1707 | | | | $R_{11-2}(s,a,c) \land \neg R_{11-2}(s,c,a)$ 1706, (*SPI*)
1708 | | | | $R_{11-3}(s,a,c) \land \neg R_{11-3}(s,c,a)$ 1706, (*SPI*)
1709 | | | | $R_{11-7}(s,a,c) \land \neg R_{11-7}(s,c,a)$ 1706, (*SPI*)
1710 | | | | $R_{11-10}(s,a,c) \land \neg R_{11-10}(s,c,a)$ 1706, (*SPI*)
1711 | | | | $R_{12-1}(s,a,c) \land \neg R_{12-1}(s,c,a)$ 1706, (*SPI*)
1712 | | | | $R_{12-2}(s,a,c) \land \neg R_{12-2}(s,c,a)$ 1706, (*SPI*)
1713 | | | | $R_{12-3}(s,a,c) \land \neg R_{12-3}(s,c,a)$ 1706, (*SPI*)
1714 | | | | $R_{12-7}(s,a,c) \land \neg R_{12-7}(s,c,a)$ 1706, (*SPI*)
1715 | | | | $R_{12-10}(s,a,c) \land \neg R_{12-10}(s,c,a)$ 1706, (*SPI*)
1716 | | | | $R_{13-1}(s,a,c) \land \neg R_{13-1}(s,c,a)$ 1706, (*SPI*)
1717 | | | | $R_{13-2}(s,a,c) \land \neg R_{13-2}(s,c,a)$ 1706, (*SPI*)
1718 | | | | $R_{13-3}(s,a,c) \land \neg R_{13-3}(s,c,a)$ 1706, (*SPI*)
1719 | | | | $R_{13-7}(s,a,c) \land \neg R_{13-7}(s,c,a)$ 1706, (*SPI*)
1720 | | | | $R_{13-10}(s,a,c) \land \neg R_{13-10}(s,c,a)$ 1706, (*SPI*)
1721 | | | | $\neg R_{11-6}(s,a,c) \land R_{11-6}(s,c,a)$ 340, 1703, (*SPT*)
1722 | | | | $\neg R_{11-4}(s,a,c) \land R_{11-4}(s,c,a)$ 1721, (*SPI*)
1723 | | | | $\neg R_{11-5}(s,a,c) \land R_{11-5}(s,c,a)$ 1721, (*SPI*)
1724 | | | | $\neg R_{11-8}(s,a,c) \land R_{11-8}(s,c,a)$ 1721, (*SPI*)
1725 | | | | $\neg R_{11-9}(s,a,c) \land R_{11-9}(s,c,a)$ 1721, (*SPI*)
1726 | | | | $\neg R_{12-4}(s,a,c) \land R_{12-4}(s,c,a)$ 1721, (*SPI*)
1727 | | | | $\neg R_{12-5}(s,a,c) \land R_{12-5}(s,c,a)$ 1721, (*SPI*)
1728 | | | | $\neg R_{12-6}(s,a,c) \land R_{12-6}(s,c,a)$ 1721, (*SPI*)
1729 | | | | $\neg R_{12-8}(s,a,c) \land R_{12-8}(s,c,a)$ 1721, (*SPI*)
1730 | | | | $\neg R_{12-9}(s,a,c) \land R_{12-9}(s,c,a)$ 1721, (*SPI*)
1731 | | | | $\neg R_{13-4}(s,a,c) \land R_{13-4}(s,c,a)$ 1721, (*SPI*)
1732 | | | | $\neg R_{13-5}(s,a,c) \land R_{13-5}(s,c,a)$ 1721, (*SPI*)
1733 | | | | $\neg R_{13-6}(s,a,c) \land R_{13-6}(s,c,a)$ 1721, (*SPI*)
1734 | | | | $\neg R_{13-8}(s,a,c) \land R_{13-8}(s,c,a)$ 1721, (*SPI*)
1735 | | | | $\neg R_{13-9}(s,a,c) \land R_{13-9}(s,c,a)$ 1721, (*SPI*)
1736 | | | | $\forall w \neg(H(w) \land \forall X(\boldsymbol{P}(X) \to \forall x \forall y((A(x) \land A(y)) \to ((X(w,x,y) \land \neg X(w,y,x)) \to (X(s,x,y) \land \neg X(s,y,x))))) \land \forall u(H(u) \to (\forall X(\boldsymbol{P}(X) \to \forall x \forall y((A(x) \land A(y)) \to ((X(u,x,y) \land \neg X(u,y,x)) \to (X(s,x,y) \land \neg X(s,y,x))))) \to u = w)))$ 178, (rep.)
1737 | | | | $\neg(H(q) \land \forall X(\boldsymbol{P}(X) \to \forall x \forall y((A(x) \land A(y)) \to ((X(q,x,y) \land \neg X(q,y,x)) \to (X(s,x,y) \land \neg X(s,y,x))))) \land \forall u(H(u) \to (\forall X(\boldsymbol{P}(X) \to \forall x \forall y((A(x) \land A(y)) \to ((X(u,x,y) \land \neg X(u,y,x)) \to (X(s,x,y) \land \neg X(s,y,x))))) \to u = q)))$ 1736, ($\forall E$)
1738 | | | | $\neg H(q) \lor \neg \forall X(\boldsymbol{P}(X) \to \forall x \forall y((A(x) \land A(y)) \to ((X(q,x,y) \land \neg X(q,y,x)) \to (X(s,x,y) \land \neg X(s,y,x))))) \lor \neg \forall u(H(u) \to (\forall X(\boldsymbol{P}(X) \to \forall x \forall y((A(x) \land A(y)) \to ((X(u,x,y) \land \neg X(u,y,x)) \to (X(s,x,y) \land \neg X(s,y,x))))) \to u = q))$ 1737, (rep.)
1739 | | | | | $\neg H(q)$ prem.
1740 | | | | | $H(q)$ 1, ($\land E$)
1741 | | | | | $\bot$ 1739, 1740, ($\neg E$)
1742 | | | | $\neg H(q) \to \bot$ 1739, 1741, ($\to I$)
1743 | | | | | $\neg \forall X(\boldsymbol{P}(X) \to \forall x \forall y((A(x) \land A(y)) \to ((X(q,x,y) \land \neg X(q,y,x)) \to (X(s,x,y) \land \neg X(s,y,x)))))$ prem.
1744 | | | | | | $\boldsymbol{P}(R_0)$ prem.
1745 | | | | | | | $\neg \forall x \forall y((A(x) \land A(y)) \to ((R_0(q,x,y) \land \neg R_0(q,y,x)) \to (R_0(s,x,y) \land \neg R_0(s,y,x))))$ prem.
1746 | | | | | | | $\forall X(\boldsymbol{P}(X) \to (X = R_{1-1} \lor \ldots \lor X = R_{13-13}))$ 3, ($\land E$)
1747 | | | | | | | $\boldsymbol{P}(R_0) \to (R_0 = R_{1-1} \lor \ldots \lor R_0 = R_{13-13})$ 1746, ($\forall E$)
1748 | | | | | | | $R_0 = R_{1-1} \lor \ldots \lor R_0 = R_{13-13}$ 1744, 1747, ($\to E$)
1749 | | | | | | | | $R_0 = R_{1-1}$ prem.
1750 | | | | | | | | $\bot$ 4, 205–207, (*SPW I*)
1751 | | | | | | | $(R_0 = R_{1-1}) \to \bot$ 1749, 1750, ($\to I$)
1752 | | | | | | | | $R_0 = R_{1-2}$ prem.
1753 | | | | | | | | $\bot$ 5, 208, 209, 1494, 1496, (*SPW I*)
1754 | | | | | | | $(R_0 = R_{1-2}) \to \bot$ 1752, 1753, ($\to I$)
1755 | | | | | | | | $R_0 = R_{1-3}$ prem.
1756 | | | | | | | | $\bot$ 6, 210, 211, 1682, (*SPW I*)
1757 | | | | | | | $(R_0 = R_{1-3}) \to \bot$ 1755, 1756, ($\to I$)
1758 | | | | | | | | $R_0 = R_{1-4}$ prem.
1759 | | | | | | | | $\bot$ 7, 212, 1683, 1547, (*SPW I*)
1760 | | | | | | | $(R_0 = R_{1-4}) \to \bot$ 1758, 1759, ($\to I$)
1761 | | | | | | | | $R_0 = R_{1-5}$ prem.
1762 | | | | | | | | $\bot$ 8, 213, 1497, 1548, (*SPW I*)
1763 | | | | | | | $(R_0 = R_{1-5}) \to \bot$ 1761, 1762, ($\to I$)
1764 | | | | | | | | $R_0 = R_{1-6}$ prem.
1765 | | | | | | | | $\bot$ 9, 1498, 1684, 1549, (*SPW I*)
1766 | | | | | | | $(R_0 = R_{1-6}) \to \bot$ 1764, 1765, ($\to I$)
1767 | | | | | | | | $R_0 = R_{1-7}$ prem.
1768 | | | | | | | | $\bot$ 10, 214, 215, (*SPW II*)
1769 | | | | | | | $(R_0 = R_{1-7}) \to \bot$ 1767, 1768, ($\to I$)
1770 | | | | | | | | $R_0 = R_{1-8}$ prem.
1771 | | | | | | | | $\bot$ 11, 1499, 1550, (*SPW II*)
1772 | | | | | | | $(R_0 = R_{1-8}) \to \bot$ 1770, 1771, ($\to I$)
1773 | | | | | | | | $R_0 = R_{1-9}$ prem.
1774 | | | | | | | | $\bot$ 12, 1685, 1551, (*SPW II*)
1775 | | | | | | | $(R_0 = R_{1-9}) \to \bot$ 1773, 1774, ($\to I$)
1776 | | | | | | | | $R_0 = R_{1-10}$ prem.
1777 | | | | | | | | $\bot$ 13, 216, 217, (*SPW II*)
1778 | | | | | | | $(R_0 = R_{1-10}) \to \bot$ 1776, 1777, ($\to I$)
1779 | | | | | | | | $R_0 = R_{1-11}$ prem.
1780 | | | | | | | | $\bot$ 14, 218, 1500, (*SPW II*)
1781 | | | | | | | $(R_0 = R_{1-11}) \to \bot$ 1779, 1780, ($\to I$)
1782 | | | | | | | | $R_0 = R_{1-12}$ prem.
1783 | | | | | | | | $\bot$ 15, 219, 1686, (*SPW II*)
1784 | | | | | | | $(R_0 = R_{1-12}) \to \bot$ 1782, 1783, ($\to I$)
1785 | | | | | | | | $R_0 = R_{1-13}$ prem.
1786 | | | | | | | | $\bot$ 16, (*SPW III*)
1787 | | | | | | | $(R_0 = R_{1-13}) \to \bot$ 1785, 1786, ($\to I$)
1788 | | | | | | | | $R_0 = R_{2-1}$ prem.
1789 | | | | | | | | $\bot$ 17, 220, 221, 1597, (*SPW I*)
1790 | | | | | | | $(R_0 = R_{2-1}) \to \bot$ 1788, 1789, ($\to I$)
1791 | | | | | | | | $R_0 = R_{2-2}$ prem.
1792 | | | | | | | | $\bot$ 18, 222–224, (*SPW I*)
1793 | | | | | | | $(R_0 = R_{2-2}) \to \bot$ 1791, 1792, ($\to I$)
1794 | | | | | | | | $R_0 = R_{2-3}$ prem.
1795 | | | | | | | | $\bot$ 19, 225, 1598, 1687, (*SPW I*)
1796 | | | | | | | $(R_0 = R_{2-3}) \to \bot$ 1794, 1795, ($\to I$)
1797 | | | | | | | | $R_0 = R_{2-4}$ prem.
1798 | | | | | | | | $\bot$ 20, 1552, 1599, 1688, (*SPW I*)
1799 | | | | | | | $(R_0 = R_{2-4}) \to \bot$ 1797, 1798, ($\to I$)
1800 | | | | | | | | $R_0 = R_{2-5}$ prem.
1801 | | | | | | | | $\bot$ 21, 226, 227, 1553, (*SPW I*)
1802 | | | | | | | $(R_0 = R_{2-5}) \to \bot$ 1800, 1801, ($\to I$)

1803 | | | | | | | | $R_0 = R_{2\text{-}6}$ prem.
1804 | | | | | | | | $\bot$ 22, 228, 1554, 1689, (*SPW I*)
1805 | | | | | | | $(R_0 = R_{2\text{-}6}) \rightarrow \bot$ 1803, 1804, ($\rightarrow I$)
1806 | | | | | | | | $R_0 = R_{2\text{-}7}$ prem.
1807 | | | | | | | | $\bot$ 23, 229, 1600, (*SPW II*)
1808 | | | | | | | $(R_0 = R_{2\text{-}7}) \rightarrow \bot$ 1806, 1807, ($\rightarrow I$)
1809 | | | | | | | | $R_0 = R_{2\text{-}8}$ prem.
1810 | | | | | | | | $\bot$ 24, 230, 1555, (*SPW II*)
1811 | | | | | | | $(R_0 = R_{2\text{-}8}) \rightarrow \bot$ 1809, 1810, ($\rightarrow I$)
1812 | | | | | | | | $R_0 = R_{2\text{-}9}$ prem.
1813 | | | | | | | | $\bot$ 25, 1556, 1690, (*SPW II*)
1814 | | | | | | | $(R_0 = R_{2\text{-}9}) \rightarrow \bot$ 1812, 1813, ($\rightarrow I$)
1815 | | | | | | | | $R_0 = R_{2\text{-}10}$ prem.
1816 | | | | | | | | $\bot$ 26, 231, 232, (*SPW II*)
1817 | | | | | | | $(R_0 = R_{2\text{-}10}) \rightarrow \bot$ 1815, 1816, ($\rightarrow I$)
1818 | | | | | | | | $R_0 = R_{2\text{-}11}$ prem.
1819 | | | | | | | | $\bot$ 27, 233, 234, (*SPW II*)
1820 | | | | | | | $(R_0 = R_{2\text{-}11}) \rightarrow \bot$ 1818, 1819, ($\rightarrow I$)
1821 | | | | | | | | $R_0 = R_{2\text{-}12}$ prem.
1822 | | | | | | | | $\bot$ 28, 1601, 1691, (*SPW II*)
1823 | | | | | | | $(R_0 = R_{2\text{-}12}) \rightarrow \bot$ 1821, 1822, ($\rightarrow I$)
1824 | | | | | | | | $R_0 = R_{2\text{-}13}$ prem.
1825 | | | | | | | | $\bot$ 29, (*SPW III*)
1826 | | | | | | | $(R_0 = R_{2\text{-}13}) \rightarrow \bot$ 1824, 1825, ($\rightarrow I$)
1827 | | | | | | | | $R_0 = R_{3\text{-}1}$ prem.
1828 | | | | | | | | $\bot$ 30, 235, 236, 1522, (*SPW I*)
1829 | | | | | | | $(R_0 = R_{3\text{-}1}) \rightarrow \bot$ 1827, 1828, ($\rightarrow I$)
1830 | | | | | | | | $R_0 = R_{3\text{-}2}$ prem.
1831 | | | | | | | | $\bot$ 31, 237, 1501, 1521, (*SPW I*)
1832 | | | | | | | $(R_0 = R_{3\text{-}2}) \rightarrow \bot$ 1830, 1831, ($\rightarrow I$)
1833 | | | | | | | | $R_0 = R_{3\text{-}3}$ prem.
1834 | | | | | | | | $\bot$ 32, 238–240, (*SPW I*)
1835 | | | | | | | $(R_0 = R_{3\text{-}3}) \rightarrow \bot$ 1833, 1834, ($\rightarrow I$)
1836 | | | | | | | | $R_0 = R_{3\text{-}4}$ prem.
1837 | | | | | | | | $\bot$ 33, 241, 242, 1557, (*SPW I*)
1838 | | | | | | | $(R_0 = R_{3\text{-}4}) \rightarrow \bot$ 1836, 1837, ($\rightarrow I$)
1839 | | | | | | | | $R_0 = R_{3\text{-}5}$ prem.
1840 | | | | | | | | $\bot$ 34, 1502, 1523, 1558, (*SPW I*)
1841 | | | | | | | $(R_0 = R_{3\text{-}5}) \rightarrow \bot$ 1839, 1840, ($\rightarrow I$)
1842 | | | | | | | | $R_0 = R_{3\text{-}6}$ prem.
1843 | | | | | | | | $\bot$ 35, 243, 1503, 1546, (*SPW I*)
1844 | | | | | | | $(R_0 = R_{3\text{-}6}) \rightarrow \bot$ 1842, 1843, ($\rightarrow I$)
1845 | | | | | | | | $R_0 = R_{3\text{-}7}$ prem.
1846 | | | | | | | | $\bot$ 36, 244, 245, (*SPW II*)
1847 | | | | | | | $(R_0 = R_{3\text{-}7}) \rightarrow \bot$ 1845, 1846, ($\rightarrow I$)
1848 | | | | | | | | $R_0 = R_{3\text{-}8}$ prem.
1849 | | | | | | | | $\bot$ 37, 1504, 1559, (*SPW II*)
1850 | | | | | | | $(R_0 = R_{3\text{-}8}) \rightarrow \bot$ 1848, 1849, ($\rightarrow I$)
1851 | | | | | | | | $R_0 = R_{3\text{-}9}$ prem.
1852 | | | | | | | | $\bot$ 38, 246, 1560, (*SPW II*)
1853 | | | | | | | $(R_0 = R_{3\text{-}9}) \rightarrow \bot$ 1851, 1852, ($\rightarrow I$)
1854 | | | | | | | | $R_0 = R_{3\text{-}10}$ prem.
1855 | | | | | | | | $\bot$ 39, 247, 1524, (*SPW II*)
1856 | | | | | | | $(R_0 = R_{3\text{-}10}) \rightarrow \bot$ 1,854 1855, ($\rightarrow I$)
1857 | | | | | | | | $R_0 = R_{3\text{-}11}$ prem.
1858 | | | | | | | | $\bot$ 40, 1505, 1525, (*SPW II*)
1859 | | | | | | | $(R_0 = R_{3\text{-}11}) \rightarrow \bot$ 1857, 1858, ($\rightarrow I$)
1860 | | | | | | | | $R_0 = R_{3\text{-}12}$ prem.
1861 | | | | | | | | $\bot$ 41, 248, 249, (*SPW II*)
1862 | | | | | | | $(R_0 = R_{3\text{-}12}) \rightarrow \bot$ 1860, 1861, ($\rightarrow I$)
1863 | | | | | | | | $R_0 = R_{3\text{-}13}$ prem.
1864 | | | | | | | | $\bot$ 42, (*SPW III*)
1865 | | | | | | | $(R_0 = R_{3\text{-}13}) \rightarrow \bot$ 1863, 1864, ($\rightarrow I$)
1866 | | | | | | | | $R_0 = R_{4\text{-}1}$ prem.
1867 | | | | | | | | $\bot$ 43, 250, 1526, 1571, (*SPW I*)
1868 | | | | | | | $(R_0 = R_{4\text{-}1}) \rightarrow \bot$ 1866, 1867, ($\rightarrow I$)
1869 | | | | | | | | $R_0 = R_{4\text{-}2}$ prem.
1870 | | | | | | | | $\bot$ 44, 1506, 1527, 1572, (*SPW I*)
1871 | | | | | | | $(R_0 = R_{4\text{-}2}) \rightarrow \bot$ 1869, 1870, ($\rightarrow I$)
1872 | | | | | | | | $R_0 = R_{4\text{-}3}$ prem.
1873 | | | | | | | | $\bot$ 45, 251, 252, 1573, (*SPW I*)
1874 | | | | | | | $(R_0 = R_{4\text{-}3}) \rightarrow \bot$ 1872, 1873, ($\rightarrow I$)
1875 | | | | | | | | $R_0 = R_{4\text{-}4}$ prem.
1876 | | | | | | | | $\bot$ 46, 253–255, (*SPW I*)
1877 | | | | | | | $(R_0 = R_{4\text{-}4}) \rightarrow \bot$ 1875, 1876, ($\rightarrow I$)
1878 | | | | | | | | $R_0 = R_{4\text{-}5}$ prem.
1879 | | | | | | | | $\bot$ 47, 256, 1507, 1528, (*SPW I*)
1880 | | | | | | | $(R_0 = R_{4\text{-}5}) \rightarrow \bot$ 1878, 1879, ($\rightarrow I$)
1881 | | | | | | | | $R_0 = R_{4\text{-}6}$ prem.
1882 | | | | | | | | $\bot$ 48, 257, 258, 1508, (*SPW I*)
1883 | | | | | | | $(R_0 = R_{4\text{-}6}) \rightarrow \bot$ 1881, 1882, ($\rightarrow I$)
1884 | | | | | | | | $R_0 = R_{4\text{-}7}$ prem.
1885 | | | | | | | | $\bot$ 49, 259, 1574, (*SPW II*)
1886 | | | | | | | $(R_0 = R_{4\text{-}7}) \rightarrow \bot$ 1884, 1885, ($\rightarrow I$)
1887 | | | | | | | | $R_0 = R_{4\text{-}8}$ prem.
1888 | | | | | | | | $\bot$ 50, 260, 1509, (*SPW II*)
1889 | | | | | | | $(R_0 = R_{4\text{-}8}) \rightarrow \bot$ 1887, 1888, ($\rightarrow I$)
1890 | | | | | | | | $R_0 = R_{4\text{-}9}$ prem.
1891 | | | | | | | | $\bot$ 51, 261, 262, (*SPW II*)
1892 | | | | | | | $(R_0 = R_{4\text{-}9}) \rightarrow \bot$ 1890, 1891, ($\rightarrow I$)
1893 | | | | | | | | $R_0 = R_{4\text{-}10}$ prem.
1894 | | | | | | | | $\bot$ 52, 1529, 1575, (*SPW II*)
1895 | | | | | | | $(R_0 = R_{4\text{-}10}) \rightarrow \bot$ 1893, 1894, ($\rightarrow I$)
1896 | | | | | | | | $R_0 = R_{4\text{-}11}$ prem.
1897 | | | | | | | | $\bot$ 53, 1510, 1530, (*SPW II*)
1898 | | | | | | | $(R_0 = R_{4\text{-}11}) \rightarrow \bot$ 1896, 1897, ($\rightarrow I$)
1899 | | | | | | | | $R_0 = R_{4\text{-}12}$ prem.
1900 | | | | | | | | $\bot$ 54, 263, 264, (*SPW II*)
1901 | | | | | | | $(R_0 = R_{4\text{-}12}) \rightarrow \bot$ 1899, 1900, ($\rightarrow I$)
1902 | | | | | | | | $R_0 = R_{4\text{-}13}$ prem.
1903 | | | | | | | | $\bot$ 55, (*SPW III*)
1904 | | | | | | | $(R_0 = R_{4\text{-}13}) \rightarrow \bot$ 1902, 1903, ($\rightarrow I$)
1905 | | | | | | | | $R_0 = R_{5\text{-}1}$ prem.
1906 | | | | | | | | $\bot$ 56, 265, 1576, 1602, (*SPW I*)
1907 | | | | | | | $(R_0 = R_{5\text{-}1}) \rightarrow \bot$ 1905, 1906 ($\rightarrow I$)
1908 | | | | | | | | $R_0 = R_{5\text{-}2}$ prem.
1909 | | | | | | | | $\bot$ 57, 266, 267, 1577, (*SPW I*)
1910 | | | | | | | $(R_0 = R_{5\text{-}2}) \rightarrow \bot$ 1908, 1909, ($\rightarrow I$)
1911 | | | | | | | | $R_0 = R_{5\text{-}3}$ prem.
1912 | | | | | | | | $\bot$ 58, 1578, 1603, 1692, (*SPW I*)
1913 | | | | | | | $(R_0 = R_{5\text{-}3}) \rightarrow \bot$ 1911, 1912, ($\rightarrow I$)
1914 | | | | | | | | $R_0 = R_{5\text{-}4}$ prem.
1915 | | | | | | | | $\bot$ 59, 268, 1604, 1693, (*SPW I*)
1916 | | | | | | | $(R_0 = R_{5\text{-}4}) \rightarrow \bot$ 1914, 1915, ($\rightarrow I$)
1917 | | | | | | | | $R_0 = R_{5\text{-}5}$ prem.
1918 | | | | | | | | $\bot$ 60, 269–271, (*SPW I*)
1919 | | | | | | | $(R_0 = R_{5\text{-}5}) \rightarrow \bot$ 1917, 1918, ($\rightarrow I$)
1920 | | | | | | | | $R_0 = R_{5\text{-}6}$ prem.
1921 | | | | | | | | $\bot$ 61, 272, 273, 1694, (*SPW I*)
1922 | | | | | | | $(R_0 = R_{5\text{-}6}) \rightarrow \bot$ 1920, 1921, ($\rightarrow I$)
1923 | | | | | | | | $R_0 = R_{5\text{-}7}$ prem.
1924 | | | | | | | | $\bot$ 62, 1579, 1605, (*SPW II*)
1925 | | | | | | | $(R_0 = R_{5\text{-}7}) \rightarrow \bot$ 1923, 1924, ($\rightarrow I$)
1926 | | | | | | | | $R_0 = R_{5\text{-}8}$ prem.
1927 | | | | | | | | $\bot$ 63, 274, 275, (*SPW II*)
1928 | | | | | | | $(R_0 = R_{5\text{-}8}) \rightarrow \bot$ 1926, 1927, ($\rightarrow I$)
1929 | | | | | | | | $R_0 = R_{5\text{-}9}$ prem.
1930 | | | | | | | | $\bot$ 64, 276, 1695, (*SPW II*)
1931 | | | | | | | $(R_0 = R_{5\text{-}9}) \rightarrow \bot$ 1929, 1930, ($\rightarrow I$)
1932 | | | | | | | | $R_0 = R_{5\text{-}10}$ prem.
1933 | | | | | | | | $\bot$ 65, 277, 1580, (*SPW II*)
1934 | | | | | | | $(R_0 = R_{5\text{-}10}) \rightarrow \bot$ 1932, 1933, ($\rightarrow I$)
1935 | | | | | | | | $R_0 = R_{5\text{-}11}$ prem.
1936 | | | | | | | | $\bot$ 66, 278, 279, (*SPW II*)
1937 | | | | | | | $(R_0 = R_{5\text{-}11}) \rightarrow \bot$ 1935, 1936, ($\rightarrow I$)
1938 | | | | | | | | $R_0 = R_{5\text{-}12}$ prem.
1939 | | | | | | | | $\bot$ 67, 1606, 1696, (*SPW II*)
1940 | | | | | | | $(R_0 = R_{5\text{-}12}) \rightarrow \bot$ 1938, 1939, ($\rightarrow I$)
1941 | | | | | | | | $R_0 = R_{5\text{-}13}$ prem.
1942 | | | | | | | | $\bot$ 68, (*SPW III*)
1943 | | | | | | | $(R_0 = R_{5\text{-}13}) \rightarrow \bot$ 1941, 1942, ($\rightarrow I$)
1944 | | | | | | | | $R_0 = R_{6\text{-}1}$ prem.
1945 | | | | | | | | $\bot$ 69, 1531, 1581, 1607, (*SPW I*)
1946 | | | | | | | $(R_0 = R_{6\text{-}1}) \rightarrow \bot$ 1944, 1945, ($\rightarrow I$)
1947 | | | | | | | | $R_0 = R_{6\text{-}2}$ prem.
1948 | | | | | | | | $\bot$ 70, 280, 1532, 1582, (*SPW I*)
1949 | | | | | | | $(R_0 = R_{6\text{-}2}) \rightarrow \bot$ 1947, 1948, ($\rightarrow I$)
1950 | | | | | | | | $R_0 = R_{6\text{-}3}$ prem.
1951 | | | | | | | | $\bot$ 71, 281, 1583, 1596, (*SPW I*)
1952 | | | | | | | $(R_0 = R_{6\text{-}3}) \rightarrow \bot$ 1950, 1951, ($\rightarrow I$)
1953 | | | | | | | | $R_0 = R_{6\text{-}4}$ prem.

1954 | | | | | | | | $\bot$ 72, 282, 283, 1608, (*SPW I*)
1955 | | | | | | | $(R_0 = R_{6-4}) \to \bot$ 1953, 1954, (→*I*)
1956 | | | | | | | | $R_0 = R_{6-5}$ prem.
1957 | | | | | | | | $\bot$ 73, 284, 285, 1533, (*SPW I*)
1958 | | | | | | | $(R_0 = R_{6-5}) \to \bot$ 1956, 1957, (→*I*)
1959 | | | | | | | | $R_0 = R_{6-6}$ prem.
1960 | | | | | | | | $\bot$ 74, 286–288, (*SPW I*)
1961 | | | | | | | $(R_0 = R_{6-6}) \to \bot$ 1959, 1960, (→*I*)
1962 | | | | | | | | $R_0 = R_{6-7}$ prem.
1963 | | | | | | | | $\bot$ 75, 1584, 1609, (*SPW II*)
1964 | | | | | | | $(R_0 = R_{6-7}) \to \bot$ 1962, 1963, (→*I*)
1965 | | | | | | | | $R_0 = R_{6-8}$ prem.
1966 | | | | | | | | $\bot$ 76, 289, 290, (*SPW II*)
1967 | | | | | | | $(R_0 = R_{6-8}) \to \bot$ 1965, 1966, (→*I*)
1968 | | | | | | | | $R_0 = R_{6-9}$ prem.
1969 | | | | | | | | $\bot$ 77, 291, 292, (*SPW II*)
1970 | | | | | | | $(R_0 = R_{6-9}) \to \bot$ 1968, 1969, (→*I*)
1971 | | | | | | | | $R_0 = R_{6-10}$ prem.
1972 | | | | | | | | $\bot$ 78, 1534, 1585, (*SPW II*)
1973 | | | | | | | $(R_0 = R_{6-10}) \to \bot$ 1971, 1972, (→*I*)
1974 | | | | | | | | $R_0 = R_{6-11}$ prem.
1975 | | | | | | | | $\bot$ 79, 293, 1535, (*SPW II*)
1976 | | | | | | | $(R_0 = R_{6-11}) \to \bot$ 1974, 1975, (→*I*)
1977 | | | | | | | | $R_0 = R_{6-12}$ prem.
1978 | | | | | | | | $\bot$ 80, 294, 1610, (*SPW II*)
1979 | | | | | | | $(R_0 = R_{6-12}) \to \bot$ 1977, 1978, (→*I*)
1980 | | | | | | | | $R_0 = R_{6-13}$ prem.
1981 | | | | | | | | $\bot$ 81, (*SPW III*)
1982 | | | | | | | $(R_0 = R_{6-13}) \to \bot$ 1980, 1981, (→*I*)
1983 | | | | | | | | $R_0 = R_{7-1}$ prem.
1984 | | | | | | | | $\bot$ 82, 295, 296, 1622, (*SPW I*)
1985 | | | | | | | $(R_0 = R_{7-1}) \to \bot$ 1983, 1984, (→*I*)
1986 | | | | | | | | $R_0 = R_{7-2}$ prem.
1987 | | | | | | | | $\bot$ 83, 297, 1511, 1621, (*SPW I*)
1988 | | | | | | | $(R_0 = R_{7-2}) \to \bot$ 1986, 1987, (→*I*)
1989 | | | | | | | | $R_0 = R_{7-3}$ prem.
1990 | | | | | | | | $\bot$ 84, 298, 299, 1637, (*SPW I*)
1991 | | | | | | | $(R_0 = R_{7-3}) \to \bot$ 1989, 1990, (→*I*)
1992 | | | | | | | | $R_0 = R_{7-4}$ prem.
1993 | | | | | | | | $\bot$ 85, 300, 1561, 1636, (*SPW I*)
1994 | | | | | | | $(R_0 = R_{7-4}) \to \bot$ 1992, 1993, (→*I*)
1995 | | | | | | | | $R_0 = R_{7-5}$ prem.
1996 | | | | | | | | $\bot$ 86, 1512, 1562, 1623, (*SPW I*)
1997 | | | | | | | $(R_0 = R_{7-5}) \to \bot$ 1995, 1996, (→*I*)
1998 | | | | | | | | $R_0 = R_{7-6}$ prem.
1999 | | | | | | | | $\bot$ 87, 1513, 1563, 1638, (*SPW I*)
2000 | | | | | | | $(R_0 = R_{7-6}) \to \bot$ 1998, 1999, (→*I*)
2001 | | | | | | | | $R_0 = R_{7-7}$ prem.
2002 | | | | | | | | $\bot$ 88, 301, 302, (*SPW II*)
2003 | | | | | | | $(R_0 = R_{7-7}) \to \bot$ 2001, 2002, (→*I*)
2004 | | | | | | | | $R_0 = R_{7-8}$ prem.
2005 | | | | | | | | $\bot$ 89, 1514, 1564, (*SPW II*)
2006 | | | | | | | $(R_0 = R_{7-8}) \to \bot$ 2004, 2005, (→*I*)
2007 | | | | | | | | $R_0 = R_{7-9}$ prem.
2008 | | | | | | | | $\bot$ 90, 1565, 1639, (*SPW II*)
2009 | | | | | | | $(R_0 = R_{7-9}) \to \bot$ 2007, 2008, (→*I*)
2010 | | | | | | | | $R_0 = R_{7-10}$ prem.
2011 | | | | | | | | $\bot$ 91, 303, 1624, (*SPW II*)
2012 | | | | | | | $(R_0 = R_{7-10}) \to \bot$ 2010, 2011, (→*I*)
2013 | | | | | | | | $R_0 = R_{7-11}$ prem.
2014 | | | | | | | | $\bot$ 92, 1515, 1625, (*SPW II*)
2015 | | | | | | | $(R_0 = R_{7-11}) \to \bot$ 2013, 2014, (→*I*)
2016 | | | | | | | | $R_0 = R_{7-12}$ prem.
2017 | | | | | | | | $\bot$ 93, 304, 1640, (*SPW II*)
2018 | | | | | | | $(R_0 = R_{7-12}) \to \bot$ 2016, 2017, (→*I*)
2019 | | | | | | | | $R_0 = R_{7-13}$ prem.
2020 | | | | | | | | $\bot$ 94, (*SPW III*)
2021 | | | | | | | $(R_0 = R_{7-13}) \to \bot$ 2019, 2020, (→*I*)
2022 | | | | | | | | $R_0 = R_{8-1}$ prem.
2023 | | | | | | | | $\bot$ 95, 1586, 1611, 1626, (*SPW I*)
2024 | | | | | | | $(R_0 = R_{8-1}) \to \bot$ 2022, 2023, (→*I*)
2025 | | | | | | | | $R_0 = R_{8-2}$ prem.
2026 | | | | | | | | $\bot$ 96, 305, 1587, 1627, (*SPW I*)
2027 | | | | | | | $(R_0 = R_{8-2}) \to \bot$ 2025, 2026, (→*I*)
2028 | | | | | | | | $R_0 = R_{8-3}$ prem.
2029 | | | | | | | | $\bot$ 97, 1588, 1612, 1641, (*SPW I*)
2030 | | | | | | | $(R_0 = R_{8-3}) \to \bot$ 2028, 2029, (→*I*)
2031 | | | | | | | | $R_0 = R_{8-4}$ prem.
2032 | | | | | | | | $\bot$ 98, 306, 1613, 1642, (*SPW I*)
2033 | | | | | | | $(R_0 = R_{8-4}) \to \bot$ 2031, 2032, (→*I*)
2034 | | | | | | | | $R_0 = R_{8-5}$ prem.
2035 | | | | | | | | $\bot$ 99, 307, 308, 1628, (*SPW I*)
2036 | | | | | | | $(R_0 = R_{8-5}) \to \bot$ 2034, 2035, (→*I*)
2037 | | | | | | | | $R_0 = R_{8-6}$ prem.
2038 | | | | | | | | $\bot$ 100, 309, 310, 1643, (*SPW I*)
2039 | | | | | | | $(R_0 = R_{8-6}) \to \bot$ 2037, 2038, (→*I*)
2040 | | | | | | | | $R_0 = R_{8-7}$ prem.
2041 | | | | | | | | $\bot$ 101, 1589, 1614, (*SPW II*)
2042 | | | | | | | $(R_0 = R_{8-7}) \to \bot$ 2040, 2041, (→*I*)
2043 | | | | | | | | $R_0 = R_{8-8}$ prem.
2044 | | | | | | | | $\bot$ 102, 311, 312, (*SPW II*)
2045 | | | | | | | $(R_0 = R_{8-8}) \to \bot$ 2043, 2044, (→*I*)
2046 | | | | | | | | $R_0 = R_{8-9}$ prem.
2047 | | | | | | | | $\bot$ 103, 313, 1644, (*SPW II*)
2048 | | | | | | | $(R_0 = R_{8-9}) \to \bot$ 2046, 2047, (→*I*)
2049 | | | | | | | | $R_0 = R_{8-10}$ prem.
2050 | | | | | | | | $\bot$ 104, 1590, 1629, (*SPW II*)
2051 | | | | | | | $(R_0 = R_{8-10}) \to \bot$ 2049, 2050, (→*I*)
2052 | | | | | | | | $R_0 = R_{8-11}$ prem.
2053 | | | | | | | | $\bot$ 105, 314, 1630, (*SPW II*)
2054 | | | | | | | $(R_0 = R_{8-11}) \to \bot$ 2052, 2053, (→*I*)
2055 | | | | | | | | $R_0 = R_{8-12}$ prem.
2056 | | | | | | | | $\bot$ 106, 1615, 1645, (*SPW II*)
2057 | | | | | | | $(R_0 = R_{8-12}) \to \bot$ 2055, 2056, (→*I*)
2058 | | | | | | | | $R_0 = R_{8-13}$ prem.
2059 | | | | | | | | $\bot$ 107, (*SPW III*)
2060 | | | | | | | $(R_0 = R_{8-13}) \to \bot$ 2058, 2059, (→*I*)
2061 | | | | | | | | $R_0 = R_{9-1}$ prem.
2062 | | | | | | | | $\bot$ 108, 1536, 1591, 1652, (*SPW I*)
2063 | | | | | | | $(R_0 = R_{9-1}) \to \bot$ 2061 2062, (→*I*)
2064 | | | | | | | | $R_0 = R_{9-2}$ prem.
2065 | | | | | | | | $\bot$ 109, 1537, 1592, 1667, (*SPW I*)
2066 | | | | | | | $(R_0 = R_{9-2}) \to \bot$ 2064, 2065, (→*I*)
2067 | | | | | | | | $R_0 = R_{9-3}$ prem.
2068 | | | | | | | | $\bot$ 110, 315, 1593, 1651, (*SPW I*)
2069 | | | | | | | $(R_0 = R_{9-3}) \to \bot$ 2067, 2068, (→*I*)
2070 | | | | | | | | $R_0 = R_{9-4}$ prem.
2071 | | | | | | | | $\bot$ 111, 316, 317, 1653, (*SPW I*)
2072 | | | | | | | $(R_0 = R_{9-4}) \to \bot$ 2070, 2071, (→*I*)
2073 | | | | | | | | $R_0 = R_{9-5}$ prem.
2074 | | | | | | | | $\bot$ 112, 318, 1538, 1666, (*SPW I*)
2075 | | | | | | | $(R_0 = R_{9-5}) \to \bot$ 2073, 2074, (→*I*)
2076 | | | | | | | | $R_0 = R_{9-6}$ prem.
2077 | | | | | | | | $\bot$ 113, 319, 320, 1668, (*SPW I*)
2078 | | | | | | | $(R_0 = R_{9-6}) \to \bot$ 2076, 2077, (→*I*)
2079 | | | | | | | | $R_0 = R_{9-7}$ prem.
2080 | | | | | | | | $\bot$ 114, 1594, 1654, (*SPW II*)
2081 | | | | | | | $(R_0 = R_{9-7}) \to \bot$ 2079, 2080, (→*I*)
2082 | | | | | | | | $R_0 = R_{9-8}$ prem.
2083 | | | | | | | | $\bot$ 115, 321, 1669, (*SPW II*)
2084 | | | | | | | $(R_0 = R_{9-8}) \to \bot$ 2082, 2083, (→*I*)
2085 | | | | | | | | $R_0 = R_{9-9}$ prem.
2086 | | | | | | | | $\bot$ 116, 322, 323, (*SPW II*)
2087 | | | | | | | $(R_0 = R_{9-9}) \to \bot$ 2085, 2086, (→*I*)
2088 | | | | | | | | $R_0 = R_{9-10}$ prem.
2089 | | | | | | | | $\bot$ 117, 1539, 1595, (*SPW II*)
2090 | | | | | | | $(R_0 = R_{9-10}) \to \bot$ 2088, 2089, (→*I*)
2091 | | | | | | | | $R_0 = R_{9-11}$ prem.
2092 | | | | | | | | $\bot$ 118, 1540, 1670, (*SPW II*)
2093 | | | | | | | $(R_0 = R_{9-11}) \to \bot$ 2091, 2092, (→*I*)
2094 | | | | | | | | $R_0 = R_{9-12}$ prem.
2095 | | | | | | | | $\bot$ 119, 324, 1655, (*SPW II*)
2096 | | | | | | | $(R_0 = R_{9-12}) \to \bot$ 2094, 2095, (→*I*)
2097 | | | | | | | | $R_0 = R_{9-13}$ prem.
2098 | | | | | | | | $\bot$ 120, (*SPW III*)
2099 | | | | | | | $(R_0 = R_{9-13}) \to \bot$ 2097, 2098, (→*I*)
2100 | | | | | | | | $R_0 = R_{10-1}$ prem.
2101 | | | | | | | | $\bot$ 121, 325, 326, 1656, (*SPW I*)
2102 | | | | | | | $(R_0 = R_{10-1}) \to \bot$ 2100, 2101, (→*I*)
2103 | | | | | | | | $R_0 = R_{10-2}$ prem.
2104 | | | | | | | | $\bot$ 122, 327, 328, 1671, (*SPW I*)

2105 | | | | | | | $(R_0 = R_{10-2}) \to \bot$ 2103, 2104, (→*I*)
2106 | | | | | | | | $R_0 = R_{10-3}$ prem.
2107 | | | | | | | | $\bot$ 123, 329, 1657, 1697, (*SPW I*)
2108 | | | | | | | $(R_0 = R_{10-3}) \to \bot$ 2106, 2107, (→*I*)
2109 | | | | | | | | $R_0 = R_{10-4}$ prem.
2110 | | | | | | | | $\bot$ 124, 1566, 1658, 1681, (*SPW I*)
2111 | | | | | | | $(R_0 = R_{10-4}) \to \bot$ 2109, 2110, (→*I*)
2112 | | | | | | | | $R_0 = R_{10-5}$ prem.
2113 | | | | | | | | $\bot$ 125, 330, 1567, 1672, (*SPW I*)
2114 | | | | | | | $(R_0 = R_{10-5}) \to \bot$ 2112, 2113, (→*I*)
2115 | | | | | | | | $R_0 = R_{10-6}$ prem.
2116 | | | | | | | | $\bot$ 126, 1568, 1673, 1698, (*SPW I*)
2117 | | | | | | | $(R_0 = R_{10-6}) \to \bot$ 2115, 2116, (→*I*)
2118 | | | | | | | | $R_0 = R_{10-7}$ prem.
2119 | | | | | | | | $\bot$ 127, 331, 1659, (*SPW II*)
2120 | | | | | | | $(R_0 = R_{10-7}) \to \bot$ 2118, 2119, (→*I*)
2121 | | | | | | | | $R_0 = R_{10-8}$ prem.
2122 | | | | | | | | $\bot$ 128, 1569, 1674, (*SPW II*)
2123 | | | | | | | $(R_0 = R_{10-8}) \to \bot$ 2121, 2122, (→*I*)
2124 | | | | | | | | $R_0 = R_{10-9}$ prem.
2125 | | | | | | | | $\bot$ 129, 1570, 1699, (*SPW II*)
2126 | | | | | | | $(R_0 = R_{10-9}) \to \bot$ 2124, 2125, (→*I*)
2127 | | | | | | | | $R_0 = R_{10-10}$ prem.
2128 | | | | | | | | $\bot$ 130, 332, 333, (*SPW II*)
2129 | | | | | | | $(R_0 = R_{10-10}) \to \bot$ 2127, 2128, (→*I*)
2130 | | | | | | | | $R_0 = R_{10-11}$ prem.
2131 | | | | | | | | $\bot$ 131, 334, 1675, (*SPW II*)
2132 | | | | | | | $(R_0 = R_{10-11}) \to \bot$ 2130, 2131, (→*I*)
2133 | | | | | | | | $R_0 = R_{10-12}$ prem.
2134 | | | | | | | | $\bot$ 132, 1660, 1700, (*SPW II*)
2135 | | | | | | | $(R_0 = R_{10-12}) \to \bot$ 2133, 2134, (→*I*)
2136 | | | | | | | | $R_0 = R_{10-13}$ prem.
2137 | | | | | | | | $\bot$ 133, (*SPW III*)
2138 | | | | | | | $(R_0 = R_{10-13}) \to \bot$ 2136, 2137, (→*I*)
2139 | | | | | | | | $R_0 = R_{11-1}$ prem.
2140 | | | | | | | | $\bot$ 134, 335, 1616, 1706, (*SPW I*)
2141 | | | | | | | $(R_0 = R_{11-1}) \to \bot$ 2139, 2140, (→*I*)
2142 | | | | | | | | $R_0 = R_{11-2}$ prem.
2143 | | | | | | | | $\bot$ 135, 336, 337, 1707, (*SPW I*)
2144 | | | | | | | $(R_0 = R_{11-2}) \to \bot$ 2142, 2143, (→*I*)
2145 | | | | | | | | $R_0 = R_{11-3}$ prem.
2146 | | | | | | | | $\bot$ 136, 1617, 1701, 1708, (*SPW I*)
2147 | | | | | | | $(R_0 = R_{11-3}) \to \bot$ 2145, 2146, (→*I*)
2148 | | | | | | | | $R_0 = R_{11-4}$ prem.
2149 | | | | | | | | $\bot$ 137, 1618, 1702, 1722, (*SPW I*)
2150 | | | | | | | $(R_0 = R_{11-4}) \to \bot$ 2148, 2149, (→*I*)
2151 | | | | | | | | $R_0 = R_{11-5}$ prem.
2152 | | | | | | | | $\bot$ 138, 338, 339, 1723, (*SPW I*)
2153 | | | | | | | $(R_0 = R_{11-5}) \to \bot$ 2151, 2152, (→*I*)
2154 | | | | | | | | $R_0 = R_{11-6}$ prem.
2155 | | | | | | | | $\bot$ 139, 340, 1703, 1721, (*SPW I*)
2156 | | | | | | | $(R_0 = R_{11-6}) \to \bot$ 2154, 2155, (→*I*)
2157 | | | | | | | | $R_0 = R_{11-7}$ prem.
2158 | | | | | | | | $\bot$ 140, 1619, 1709, (*SPW II*)
2159 | | | | | | | $(R_0 = R_{11-7}) \to \bot$ 2157, 2158, (→*I*)
2160 | | | | | | | | $R_0 = R_{11-8}$ prem.
2161 | | | | | | | | $\bot$ 141, 341, 1724, (*SPW II*)
2162 | | | | | | | $(R_0 = R_{11-8}) \to \bot$ 2160, 2161, (→*I*)
2163 | | | | | | | | $R_0 = R_{11-9}$ prem.
2164 | | | | | | | | $\bot$ 142, 1704, 1725, (*SPW II*)
2165 | | | | | | | $(R_0 = R_{11-9}) \to \bot$ 2163, 2164, (→*I*)
2166 | | | | | | | | $R_0 = R_{11-10}$ prem.
2167 | | | | | | | | $\bot$ 143, 342, 1710, (*SPW II*)
2168 | | | | | | | $(R_0 = R_{11-10}) \to \bot$ 2166, 2167, (→*I*)
2169 | | | | | | | | $R_0 = R_{11-11}$ prem.
2170 | | | | | | | | $\bot$ 144, 343, 344, (*SPW II*)
2171 | | | | | | | $(R_0 = R_{11-11}) \to \bot$ 2169, 2170, (→*I*)
2172 | | | | | | | | $R_0 = R_{11-12}$ prem.
2173 | | | | | | | | $\bot$ 145, 1620, 1705, (*SPW II*)
2174 | | | | | | | $(R_0 = R_{11-12}) \to \bot$ 2172, 2173, (→*I*)
2175 | | | | | | | | $R_0 = R_{11-13}$ prem.
2176 | | | | | | | | $\bot$ 146, (*SPW III*)
2177 | | | | | | | $(R_0 = R_{11-13}) \to \bot$ 2175, 2176, (→*I*)
2178 | | | | | | | | $R_0 = R_{12-1}$ prem.
2179 | | | | | | | | $\bot$ 147, 345, 1541, 1711, (*SPW I*)
2180 | | | | | | | $(R_0 = R_{12-1}) \to \bot$ 2178 2179, (→*I*)
2181 | | | | | | | | $R_0 = R_{12-2}$ prem.
2182 | | | | | | | | $\bot$ 148, 1516, 1542, 1712, (*SPW I*)
2183 | | | | | | | $(R_0 = R_{12-2}) \to \bot$ 2181, 2182, (→*I*)
2184 | | | | | | | | $R_0 = R_{12-3}$ prem.
2185 | | | | | | | | $\bot$ 149, 346, 347, 1713, (*SPW I*)
2186 | | | | | | | $(R_0 = R_{12-3}) \to \bot$ 2184, 2185, (→*I*)
2187 | | | | | | | | $R_0 = R_{12-4}$ prem.
2188 | | | | | | | | $\bot$ 150, 348, 349, 1726,(*SPW I*)
2189 | | | | | | | $(R_0 = R_{12-4}) \to \bot$ 2187, 2188, (→*I*)
2190 | | | | | | | | $R_0 = R_{12-5}$ prem.
2191 | | | | | | | | $\bot$ 151, 1517, 1543, 1727, (*SPW I*)
2192 | | | | | | | $(R_0 = R_{12-5}) \to \bot$ 2190, 2191, (→*I*)
2193 | | | | | | | | $R_0 = R_{12-6}$ prem.
2194 | | | | | | | | $\bot$ 152, 350, 1518, 1728, (*SPW I*)
2195 | | | | | | | $(R_0 = R_{12-6}) \to \bot$ 2193, 2194, (→*I*)
2196 | | | | | | | | $R_0 = R_{12-7}$ prem.
2197 | | | | | | | | $\bot$ 153, 351, 1714, (*SPW II*)
2198 | | | | | | | $(R_0 = R_{12-7}) \to \bot$ 2196, 2197, (→*I*)
2199 | | | | | | | | $R_0 = R_{12-8}$ prem.
2200 | | | | | | | | $\bot$ 154, 1519, 1729, (*SPW II*)
2201 | | | | | | | $(R_0 = R_{12-8}) \to \bot$ 2199, 2200, (→*I*)
2202 | | | | | | | | $R_0 = R_{12-9}$ prem.
2203 | | | | | | | | $\bot$ 155, 352, 1730, (*SPW II*)
2204 | | | | | | | $(R_0 = R_{12-9}) \to \bot$ 2202, 2203, (→*I*)
2205 | | | | | | | | $R_0 = R_{12-10}$ prem.
2206 | | | | | | | | $\bot$ 156, 1544, 1715, (*SPW II*)
2207 | | | | | | | $(R_0 = R_{12-10}) \to \bot$ 2205 2206, (→*I*)
2208 | | | | | | | | $R_0 = R_{12-11}$ prem.
2209 | | | | | | | | $\bot$ 157, 1520, 1545, (*SPW II*)
2210 | | | | | | | $(R_0 = R_{12-11}) \to \bot$ 2208, 2209, (→*I*)
2211 | | | | | | | | $R_0 = R_{12-12}$ prem.
2212 | | | | | | | | $\bot$ 158, 353, 354, (*SPW II*)
2213 | | | | | | | $(R_0 = R_{12-12}) \to \bot$ 2211, 2212, (→*I*)
2214 | | | | | | | | $R_0 = R_{12-13}$ prem.
2215 | | | | | | | | $\bot$ 159, (*SPW III*)
2216 | | | | | | | $(R_0 = R_{12-13}) \to \bot$ 2214, 2215, (→*I*)
2217 | | | | | | | | $R_0 = R_{13-1}$ prem.
2218 | | | | | | | | $\bot$ 160, 1631, 1661, 1716, (*SPW I*)
2219 | | | | | | | $(R_0 = R_{13-1}) \to \bot$ 2217, 2218, (→*I*)
2220 | | | | | | | | $R_0 = R_{13-2}$ prem.
2221 | | | | | | | | $\bot$ 161, 1632, 1676, 1717, (*SPW I*)
2222 | | | | | | | $(R_0 = R_{13-2}) \to \bot$ 2220, 2221, (→*I*)
2223 | | | | | | | | $R_0 = R_{13-3}$ prem.
2224 | | | | | | | | $\bot$ 162, 1646, 1662, 1718, (*SPW I*)
2225 | | | | | | | $(R_0 = R_{13-3}) \to \bot$ 2223, 2224, (→*I*)
2226 | | | | | | | | $R_0 = R_{13-4}$ prem.
2227 | | | | | | | | $\bot$ 163, 1647, 1663, 1731, (*SPW I*)
2228 | | | | | | | $(R_0 = R_{13-4}) \to \bot$ 2226, 2227, (→*I*)
2229 | | | | | | | | $R_0 = R_{13-5}$ prem.
2230 | | | | | | | | $\bot$ 164, 1633, 1677, 1732, (*SPW I*)
2231 | | | | | | | $(R_0 = R_{13-5}) \to \bot$ 2229, 2230, (→*I*)
2232 | | | | | | | | $R_0 = R_{13-6}$ prem.
2233 | | | | | | | | $\bot$ 165, 1648, 1678, 1733, (*SPW I*)
2234 | | | | | | | $(R_0 = R_{13-6}) \to \bot$ 2232, 2233, (→*I*)
2235 | | | | | | | | $R_0 = R_{13-7}$ prem.
2236 | | | | | | | | $\bot$ 166, 1664, 1719, (*SPW II*)
2237 | | | | | | | $(R_0 = R_{13-7}) \to \bot$ 2235, 2236, (→*I*)
2238 | | | | | | | | $R_0 = R_{13-8}$ prem.
2239 | | | | | | | | $\bot$ 167, 1679, 1734, (*SPW II*)
2240 | | | | | | | $(R_0 = R_{13-8}) \to \bot$ 2238, 2239, (→*I*)
2241 | | | | | | | | $R_0 = R_{13-9}$ prem.
2242 | | | | | | | | $\bot$ 168, 1649, 1735, (*SPW II*)
2243 | | | | | | | $(R_0 = R_{13-9}) \to \bot$ 2241, 2242, (→*I*)
2244 | | | | | | | | $R_0 = R_{13-10}$ prem.
2245 | | | | | | | | $\bot$ 169, 1634, 1720, (*SPW II*)
2246 | | | | | | | $(R_0 = R_{13-10}) \to \bot$ 2244, 2245, (→*I*)
2247 | | | | | | | | $R_0 = R_{13-11}$ prem.
2248 | | | | | | | | $\bot$ 170, 1635, 1680, (*SPW II*)
2249 | | | | | | | $(R_0 = R_{13-11}) \to \bot$ 2247, 2248, (→*I*)
2250 | | | | | | | | $R_0 = R_{13-12}$ prem.
2251 | | | | | | | | $\bot$ 171, 1650, 1665, (*SPW II*)
2252 | | | | | | | $(R_0 = R_{13-12}) \to \bot$ 2250, 2251, (→*I*)
2253 | | | | | | | | $R_0 = R_{13-13}$ prem.
2254 | | | | | | | | $\bot$ 172, (*SPW III*)
2255 | | | | | | | $(R_0 = R_{13-13}) \to \bot$ 2253, 2254, (→*I*)

2256 | | | | | | | $\bot$ 1748, formulas $(R_0 = R_{1-1}) \rightarrow \bot, \ldots, (R_0 = R_{13-13}) \rightarrow \bot$ from 1751 to 2255, $(\vee E)$
2257 | | | | | | $\forall x \forall y((A(x) \wedge A(y)) \rightarrow ((R_0(q,x,y) \wedge \neg R_0(q,y,x)) \rightarrow (R_0(s,x,y) \wedge \neg R_0(s,y,x)))))$ 1745, 2256, (DNE)
2258 | | | | | $\boldsymbol{P}(R_0) \rightarrow \forall x \forall y((A(x) \wedge A(y)) \rightarrow ((R_0(q,x,y) \wedge \neg R_0(q,y,x)) \rightarrow (R_0(s,x,y) \wedge \neg R_0(s,y,x)))))$ 1744, 2257, $(\rightarrow I)$
2259 | | | | | $\forall X(\boldsymbol{P}(X) \rightarrow \forall x \forall y((A(x) \wedge A(y)) \rightarrow ((X(q,x,y) \wedge \neg X(q,y,x)) \rightarrow (X(s,x,y) \wedge \neg X(s,y,x)))))$ 2258, $(\forall I)$
2260 | | | | | $\bot$ 1743, 2259, $(\neg E)$
2261 | | | | $\neg \forall X(\boldsymbol{P}(X) \rightarrow \forall x \forall y((A(x) \wedge A(y)) \rightarrow ((X(q,x,y) \wedge \neg X(q,y,x)) \rightarrow (X(s,x,y) \wedge \neg X(s,y,x))))) \rightarrow \bot$ 1743, 2260, $(\rightarrow I)$
2262 | | | | | $\neg \forall u(H(u) \rightarrow (\forall X(\boldsymbol{P}(X) \rightarrow \forall x \forall y((A(x) \wedge A(y)) \rightarrow ((X(u,x,y) \wedge \neg X(u,y,x)) \rightarrow (X(s,x,y) \wedge \neg X(s,y,x))))) \rightarrow u = q))$ prem.
2263 | | | | | | $H(h)$ prem.
2264 | | | | | | | $\forall X(\boldsymbol{P}(X) \rightarrow \forall x \forall y((A(x) \wedge A(y)) \rightarrow ((X(h,x,y) \wedge \neg X(h,y,x)) \rightarrow (X(s,x,y) \wedge \neg X(s,y,x)))))$ prem.
2265 | | | | | | | | $h \neq q$ prem.
2266 | | | | | | | | $\forall x(H(x) \rightarrow (x = p \vee x = q))$ 1, $(\wedge E)$
2267 | | | | | | | | $H(h) \rightarrow (h = p \vee h = q)$ 2266, $(\forall E)$
2268 | | | | | | | | $h = p \vee h = q$ 2263, 2267, $(\rightarrow E)$
2269 | | | | | | | | | $h = p$ prem.
2270 | | | | | | | | | $\forall X(\boldsymbol{P}(X) \rightarrow \forall x \forall y((A(x) \wedge A(y)) \rightarrow ((X(p,x,y) \wedge \neg X(p,y,x)) \rightarrow (X(s,x,y) \wedge \neg X(s,y,x)))))$ 2264, 2269, $(=E)$
2271 | | | | | | | | | $\boldsymbol{P}(R_{1-2}) \rightarrow \forall x \forall y((A(x) \wedge A(y)) \rightarrow ((R_{1-2}(p,x,y) \wedge \neg R_{1-2}(p,y,x)) \rightarrow (R_{1-2}(s,x,y) \wedge \neg R_{1-2}(s,y,x))))$ 2270, $(\forall E)$
2272 | | | | | | | | | $\boldsymbol{P}(R_{1-2})$ 3, $(\wedge E)$
2273 | | | | | | | | | $\forall x \forall y((A(x) \wedge A(y)) \rightarrow ((R_{1-2}(p,x,y) \wedge \neg R_{1-2}(p,y,x)) \rightarrow (R_{1-2}(s,x,y) \wedge \neg R_{1-2}(s,y,x))))$ 2271, 2272, $(\rightarrow E)$
2274 | | | | | | | | | $(A(b) \wedge A(c)) \rightarrow ((R_{1-2}(p,b,c) \wedge \neg R_{1-2}(p,c,b)) \rightarrow (R_{1-2}(s,b,c) \wedge \neg R_{1-2}(s,c,b)))$ 2273, $(\forall E)$
2275 | | | | | | | | | $A(b) \wedge A(c)$ 2, $(\wedge E)$
2276 | | | | | | | | | $(R_{1-2}(p,b,c) \wedge \neg R_{1-2}(p,c,b)) \rightarrow (R_{1-2}(s,b,c) \wedge \neg R_{1-2}(s,c,b))$ 2274, 2275, $(\rightarrow E)$
2277 | | | | | | | | | $R_{1-2}(p,b,c) \wedge \neg R_{1-2}(p,c,b)$ 5, $(\wedge E)$
2278 | | | | | | | | | $R_{1-2}(s,b,c) \wedge \neg R_{1-2}(s,c,b)$ 2276, 2277, $(\rightarrow E)$
2279 | | | | | | | | | $R_{1-2}(s,b,c)$ 2278, $(\wedge E)$
2280 | | | | | | | | | $\bot$ 1494, 2279, $(\neg E)$
2281 | | | | | | | | $(h = p) \rightarrow \bot$ 2269, 2280, $(\rightarrow I)$
2282 | | | | | | | | | $h = q$ prem.
2283 | | | | | | | | | $q \neq q$ 2265, 2282, $(=E)$
2284 | | | | | | | | | $\bot$ 2283, $(\neg E)$
2285 | | | | | | | | $(h = q) \rightarrow \bot$ 2282, 2284, $(\rightarrow I)$
2286 | | | | | | | | $\bot$ 2268, 2281, 2285, $(\vee E)$
2287 | | | | | | | $h = q$ 2265, 2286, (DNE)
2288 | | | | | | $\forall X(\boldsymbol{P}(X) \rightarrow \forall x \forall y((A(x) \wedge A(y)) \rightarrow ((X(h,x,y) \wedge \neg X(h,y,x)) \rightarrow (X(s,x,y) \wedge \neg X(s,y,x))))) \rightarrow h = q$ 2264, 2287, $(\rightarrow I)$
2289 | | | | | $H(h) \rightarrow (\forall X(\boldsymbol{P}(X) \rightarrow \forall x \forall y((A(x) \wedge A(y)) \rightarrow ((X(h,x,y) \wedge \neg X(h,y,x)) \rightarrow (X(s,x,y) \wedge \neg X(s,y,x))))) \rightarrow h = q)$ 2263, 2288, $(\rightarrow I)$
2290 | | | | | $\forall u(H(u) \rightarrow (\forall X(\boldsymbol{P}(X) \rightarrow \forall x \forall y((A(x) \wedge A(y)) \rightarrow ((X(u,x,y) \wedge \neg X(u,y,x)) \rightarrow (X(s,x,y) \wedge \neg X(s,y,x))))) \rightarrow u = q))$ 2289, $(\forall I)$
2291 | | | | | $\bot$ 2262, 2290, $(\neg E)$
2292 | | | | $\neg \forall u(H(u) \rightarrow (\forall X(\boldsymbol{P}(X) \rightarrow \forall x \forall y((A(x) \wedge A(y)) \rightarrow ((X(u,x,y) \wedge \neg X(u,y,x)) \rightarrow (X(s,x,y) \wedge \neg X(s,y,x))))) \rightarrow u = q)) \rightarrow \bot$ 2262, 2291, $(\rightarrow I)$
2293 | | | | $\bot$ 1738, 1742, 2261, 2292, $(\vee E)$
2294 | | | $R_{1-2}(s,c,b) \rightarrow \bot$ 1496, 2293, $(\rightarrow I)$
2295 | | | | $\neg R_{1-2}(s,c,b)$ prem.
2296 | | | | $\boldsymbol{P}(R_{1-2}) \rightarrow \forall x \forall y((A(x) \wedge A(y)) \rightarrow (R_{1-2}(s,x,y) \vee R_{1-2}(s,y,x)))$ 174, $(\forall E)$
2297 | | | | $\boldsymbol{P}(R_{1-2})$ 3, $(\wedge E)$
2298 | | | | $\forall x \forall y((A(x) \wedge A(y)) \rightarrow (R_{1-2}(s,x,y) \vee R_{1-2}(s,y,x)))$ 2296, 2297, $(\rightarrow E)$
2299 | | | | $(A(b) \wedge A(c)) \rightarrow (R_{1-2}(s,b,c) \vee R_{1-2}(s,c,b))$ 2298, $(\forall E)$
2300 | | | | $A(b) \wedge A(c)$ 2, $(\wedge E)$
2301 | | | | $R_{1-2}(s,b,c) \vee R_{1-2}(s,c,b)$ 2299, 2300, $(\rightarrow E)$
2302 | | | | | $R_{1-2}(s,b,c)$ prem.
2303 | | | | | $\bot$ 1494, 2302, $(\neg E)$
2304 | | | | $R_{1-2}(s,b,c) \rightarrow \bot$ 2302, 2303, $(\rightarrow I)$
2305 | | | | | $R_{1-2}(s,c,b)$ prem.
2306 | | | | | $\bot$ 2295, 2305, $(\neg E)$
2307 | | | | $R_{1-2}(s,c,b) \rightarrow \bot$ 2305, 2306, $(\rightarrow I)$
2308 | | | | $\bot$ 2301, 2304, 2307, $(\vee E)$
2309 | | | $\neg R_{1-2}(s,c,b) \rightarrow \bot$ 2295, 2308, $(\rightarrow I)$
2310 | | | $\bot$ 1495, 2294, 2309, $(\vee E)$
2311 | | $\neg R_{1-2}(s,b,c) \rightarrow \bot$ 1494, 2310, $(\rightarrow I)$
2312 | | $\bot$ 361, 1493, 2311, $(\vee E)$
2313 | $\exists w(H(w) \wedge \forall X(\boldsymbol{P}(X) \rightarrow \forall x \forall y((A(x) \wedge A(y)) \rightarrow ((X(w,x,y) \wedge \neg X(w,y,x)) \rightarrow (X(s,x,y) \wedge \neg X(s,y,x))))) \wedge \forall u(H(u) \rightarrow (\forall X(\boldsymbol{P}(X) \rightarrow \forall x \forall y((A(x) \wedge A(y)) \rightarrow ((X(u,x,y) \wedge \neg X(u,y,x)) \rightarrow (X(s,x,y) \wedge \neg X(s,y,x))))) \rightarrow u = w)))$ 178, 2312, (DNE)
