# Supplementary material for: A full formal representation of Arrow’s impossibility theorem
Source: PLoS One. 2026 Feb 24;21(2):e0343069. doi: 10.1371/journal.pone.0343069 (PMC12931778; doi:10.1371/journal.pone.0343069)
Supplement: Attachment — Submitted filename: Dear authors.docx [file pone.0343069.s002.docx]

Dear authors:

This paper is technically sound and has strong, albeit somewhat limited in application, conclusions. It certainly merits publication, although it needs some reworking. Please consider the following:

0 – General:

Although the language you employ is mostly precise, the text is very dry. I understand your approach of conducting a full logical proof with minimal use of verbal remarks, but it can alienate non-specialist readers. This level of abstraction works against the broader dissemination of your work. Please consider adding illustrative examples and descriptions less reliant on pure logical expressions.

1 – Abstract

Your abstract is very short. It fails to properly place your work within the existing literature and does not clearly present your contributions (which are methodological in nature).

2 – Introduction:

Your literature review is comprehensive regarding foundational works, but narrow regarding current literature. I think your work would benefit if you could properly position it within current investigations in the social sciences, especially game theory approaches and the relaxation of some hypotheses, as done in Stomper (2014) or Elahi (2017), or even in opposing perspectives such as Kuhn’s non-uniqueness of algorithms [Okasha (2011)]. Also, your references are rich in logical/mathematical sources, but could be enriched with the epistemological and philosophical significance of Arrow’s impossibility theorem, given its interdisciplinary nature.

In my opinion, the language used to describe past proofs is sometimes quite harsh and should be toned down in favor of properly acknowledging their historical importance.

I think your last two paragraphs, regarding related studies, could be expanded—especially in terms of fully exploring what mechanized reasoning entails.

The main research question and methodological approach are stated in the third-to-last paragraph and should be reworked and expanded. I believe the final statement—that this proof is what Arrow himself would have sought—is quite strong and should be reconsidered. The limitations of a second-order logic proof should also be presented.

Finally, I think you should highlight that your main contribution is methodological, not theoretical, since it lacks any economic reasoning insight.

3 – Stating the impossibility theorem and translation of the premises:

As it stands, Sections 2 and 3 are the strongest parts of the paper. The balance between verbal descriptions and logical expressions is precise, and applying the same approach throughout the paper would be beneficial.

4 – Proof of the theorem

Your proof is technically sound and successfully formalizes a second-order logic proof of Arrow’s impossibility theorem. Thus, it fully addresses your research question, but I believe this section has some major issues that, if resolved, could greatly enhance the paper.

Sections 4.1–4.3, although necessary, are quite repetitive. The diagrams, in their current form, are not very helpful in sketching the proof and could be reworked. Also, the dryness of Section 4.2 hinders the understanding of the proof’s expansion. The text could accommodate more verbose descriptors. While this is a coherent choice given your overall approach, it is ultimately detrimental to the paper. I think these sections could rely more on meta-descriptors, showcasing the overall procedure instead of presenting a step-by-step proof. Since these are nested in nature, the logical pattern could be emphasized.

Additionally, although the Supplementary Material is readily available, the crux of the proofs developed there should be highlighted on pages 14 and 19.

5 – Conclusion

This section is quite succinct. It could accommodate a more thorough discussion, particularly regarding the economic insights and the limitations of the second-order logic approach. Also, it would benefit from properly placing your proof in the historical context of previous efforts and in epistemic/philosophical discussions. Since your paper may attract readers from other fields of the social sciences—due to the importance of Arrow’s impossibility theorem—you should articulate the relevance of your contribution in more general terms.
