# Supplementary material for: A full formal representation of Arrow’s impossibility theorem
Source: PLoS One. 2026 Feb 24;21(2):e0343069. doi: 10.1371/journal.pone.0343069 (PMC12931778; doi:10.1371/journal.pone.0343069)
Supplement: Attachment — Submitted filename: Response to Reviewers.docx [file pone.0343069.s004.docx]

**0 – General:**

**Although the language you employ is mostly precise, the text is very dry. I understand your approach of conducting a full logical proof with minimal use of verbal remarks, but it can alienate non-specialist readers. This level of abstraction works against the broader dissemination of your work. Please consider adding illustrative examples and descriptions less reliant on pure logical expressions.**

**Response:** I have added Section 3.2 to present an example illustrating how a proof unfolds using techniques from logic. Additionally, Section 4 has been revised to include the subsections of non-technical overviews alongside the technical explanations.

**1 – Abstract**

**Your abstract is very short. It fails to properly place your work within the existing literature and does not clearly present your contributions (which are methodological in nature).**

**Response:** I have rewritten the abstract. The present research is firmly situated within the context of previous literature, with particular attention to the respective merits of both the prior studies and the current work. Furthermore, the contributions of the paper are clearly articulated. Please check the revised abstract.

**2 – Introduction:**

**Your literature review is comprehensive regarding foundational works, but narrow regarding current literature. I think your work would benefit if you could properly position it within current investigations in the social sciences, especially game theory approaches and the relaxation of some hypotheses, as done in Stomper (2014) or Elahi (2017), or even in opposing perspectives such as Kuhn’s non-uniqueness of algorithms [Okasha (2011)]. Also, your references are rich in logical/mathematical sources, but could be enriched with the epistemological and philosophical significance of Arrow’s impossibility theorem, given its interdisciplinary nature.**

**Response:** The sixth paragraph of the Introduction, which begins with "The scholarship...", summarizes the broader scientific implications—including those arising from relaxed assumptions, game-theoretic perspectives, and other approaches—as well as the epistemological and philosophical significance of the present study.

I have carefully read the articles suggested by the reviewer, Stomper (2014) and Elahi (2017). However, for my reading, it seems that these works do not directly align with the focus of the present study, and I have chosen not to cite them. Instead, as cited in the sixth paragraph, I have incorporated alternative references that more appropriately support the argument. The third reference suggested by the reviewer, Okasha (2011), has been cited in the revised manuscript.

**In my opinion, the language used to describe past proofs is sometimes quite harsh and should be toned down in favor of properly acknowledging their historical importance.**

**Response:** I have softened the critical tone toward previous studies. Please check the revised text.

**I think your last two paragraphs, regarding related studies, could be expanded—especially in terms of fully exploring what mechanized reasoning entails.**

**Response:** In the fifth paragraph of the Introduction, I have clarified the relationship between the present study and prior work on mechanized reasoning and formal logic. In addition, I have incorporated a newly identified reference—Eastaugh (2025)—which was found after the initial submission.

**The main research question and methodological approach are stated in the third-to-last paragraph and should be reworked and expanded. I believe the final statement—that this proof is what Arrow himself would have sought—is quite strong and should be reconsidered. The limitations of a second-order logic proof should also be presented.**

**Response:** From the third paragraph to the final paragraph of the Introduction, I have restructured the text. In response to the reviewer’s concern, the sentence including “Arrow would have sought to achieve...” has been removed. A discussion of the limitations of second-order logic has been added to the seventh paragraph.

**Finally, I think you should highlight that your main contribution is methodological, not theoretical, since it lacks any economic reasoning insight.**

**Response:** This remark has been added as the final sentence of the fourth paragraph of the Introduction.

**3 – Stating the impossibility theorem and translation of the premises:**

**As it stands, Sections 2 and 3 are the strongest parts of the paper. The balance between verbal descriptions and logical expressions is precise, and applying the same approach throughout the paper would be beneficial.**

**Response:** In accordance with the reviewer’s suggestion, no substantive changes were made in Sections 2 and 3.1 of the revised manuscript, apart from minor adjustments resulting from edits elsewhere and reference renumbering. All numbered display formulas have been reformatted using Mathtype.

As mentioned at the beginning, I have added a simple example of proof theory as Section 3.2.

**4 – Proof of the theorem**

**Your proof is technically sound and successfully formalizes a second-order logic proof of Arrow’s impossibility theorem. Thus, it fully addresses your research question, but I believe this section has some major issues that, if resolved, could greatly enhance the paper.**

**Sections 4.1–4.3, although necessary, are quite repetitive. The diagrams, in their current form, are not very helpful in sketching the proof and could be reworked. Also, the dryness of Section 4.2 hinders the understanding of the proof’s expansion. The text could accommodate more verbose descriptors. While this is a coherent choice given your overall approach, it is ultimately detrimental to the paper. I think these sections could rely more on meta-descriptors, showcasing the overall procedure instead of presenting a step-by-step proof. Since these are nested in nature, the logical pattern could be emphasized.**

**Response:** In accordance with the reviewer’s suggestion, I have added non-technical overviews to Section 4, each followed by a subsection presenting the corresponding technical details. Additionally, I have inserted a sentence at the beginning of Section 4 indicating that the overall flow of the proof can be grasped by reading only the non-technical parts and skipping the technical ones: “The subsections on technical details may be skipped if one is concerned with the broad outline of the proof.”

As part of this revision, I reintegrated a portion of the proof for case *I* = 3 and *J* = 3, which had previously been separated into S2 Appendix B, back into the main text. Consequently, the technical explanation in Section 4.2.2 is now longer than in the originally submitted manuscript. However, I made this change in light of the newly added non-technical overview and the benefit of reducing the number of supplementary materials from two to one.

**Additionally, although the Supplementary Material is readily available, the crux of the proofs developed there should be highlighted on pages 14 and 19.**

**Response:** I appreciate the reviewer’s suggestion. The explanation of the crux of the proofs—such as the case distinctions, the use of proof by contradiction, and the nested structure—has been incorporated into the newly added non-technical overviews. Accordingly, I did not make further additions to the technical sections in this regard.

**5 – Conclusion**

**This section is quite succinct. It could accommodate a more thorough discussion, particularly regarding the economic insights and the limitations of the second-order logic approach. Also, it would benefit from properly placing your proof in the historical context of previous efforts and in epistemic/philosophical discussions. Since your paper may attract readers from other fields of the social sciences—due to the importance of Arrow’s impossibility theorem—you should articulate the relevance of your contribution in more general terms.**

**Response:** The concluding section has been substantially expanded. It now consists of separate paragraphs addressing, in order: the relationship to existing research (emphasizing the complementary contributions of prior studies and the present work), the economic insights, and the epistemological and philosophical implications. As the limitations of second-order logic are discussed in the Introduction, I do not include it in the Conclusion.

**Response:** The references have been rearranged to comply with the journal’s citation style, which requires them to appear in the order of citation.
